# Characteristics of *Staphylococcus haemolyticus* Isolated from Humans and Animals

**DOI:** 10.3390/ijms27198905

**Published:** 2026-10-07

**Authors:** Paulina Prorok, Magdalena Siedlecka, Magdalena Karwańska, Milena Skrok, Marta Miszczak, Maciej Wernecki, Marta Książczyk, Krzysztof Rypuła, Karolina Bierowiec

**Affiliations:** 1 Department of Epizootiology and Clinic of Birds and Exotic Animals, Division of Infectious Diseases and Veterinary Sciences, 50-365 Wrocław, Poland; magdalena.siedlecka@upwr.edu.pl (M.S.); magdalena.karwanska@upwr.edu.pl (M.K.); milena.skrok@upwr.edu.pl (M.S.); marta.miszczak@upwr.edu.pl (M.M.); krzysztof.rypula@upwr.edu.pl (K.R.); 2 Department of Microbiology, Faculty of Biological Sciences, University of Wroclaw, 51-148 Wrocław, Poland; maciej.wernecki@uwr.edu.pl (M.W.); marta.ksiazczyk@uwr.edu.pl (M.K.)

**Keywords:** One Health, public health, epidemiology, CoNS, *Staphylococcus haemolyticus*

## Abstract

*Staphylococcus haemolyticus* is an opportunistic coagulase-negative staphylococcus increasingly recognized as an important reservoir of antimicrobial resistance within the One Health framework. However, comparative studies simultaneously evaluating isolates from humans and companion animals remain limited. This study characterized 113 *S. haemolyticus* isolates recovered from clinically healthy and affected humans and cats and from clinically healthy dogs using phenotypic, molecular, proteomic, and genomic approaches. Multidrug resistance was identified in 45.13% (51/113) of isolates. Whole-genome sequencing of 35 selected isolates revealed substantial genomic diversity, with no clear clustering according to host species or clinical status within the analysed subset. The VFDB-associated loci *tufA*, *clpC*, and *clpP* were detected in all sequenced isolates; however, their presence does not imply expression or a direct contribution to virulence-related phenotypes. Acquired antimicrobial resistance determinants showed variable distributions across the analysed genomes. Within the WGS subset, *mecA* showed a host-associated difference that remained significant after false discovery rate correction in a one-isolate-per-host sensitivity analysis, whereas the initially observed association for *blaZ*/PC1-family determinants was not retained after this sensitivity analysis. No acquired resistance determinant was significantly associated with clinical status. Among the full collection of 113 isolates, *blaZ* (53.98%) and *tetL* (33.63%) were the most frequently detected resistance genes by PCR. Genotype–phenotype concordance varied according to the antimicrobial agent and phenotypic testing method, indicating that resistance-gene detection alone was not uniformly predictive of phenotypic susceptibility. Biofilm formation, assessed in all 113 isolates, increased markedly at 39 °C despite heterogeneous, strain-dependent changes in planktonic growth. Eight representative isolates evaluated in the *Galleria mellonella* model showed clear inoculum-dependent and strain-variable larval mortality; however, the assay was not designed to formally assess host-species- or clinical status-associated differences. Overall, the findings demonstrate substantial genomic and phenotypic overlap among human and companion-animal *S. haemolyticus* isolates, together with selected host-associated differences and marked strain-level variability, supporting the value of integrated One Health surveillance.

## 1. Introduction

*Staphylococcus haemolyticus* (*S. haemolyticus*) is a coagulase-negative staphylococcus (CoNS) that was historically considered a clinically insignificant component of the skin microbiota of humans and, to some extent, animals [1]. In recent years, increasing evidence has indicated that this microorganism represents an important opportunistic pathogen, particularly in healthcare-associated settings [2,3]. Infections caused by *S. haemolyticus* occur predominantly in immunocompromised patients and in individuals with implanted medical devices and are often chronic and difficult to treat [1,4]. The species *S. haemolyticus* was formally described as a distinct taxonomic entity in 1975 after previously being grouped with other coagulase-negative staphylococci because of phenotypic similarities [5]. The species name refers to the observed haemolytic activity. However, subsequent studies have shown that the pathogenicity of *S. haemolyticus* is primarily associated with its ability to adapt, persist, and efficiently colonize the host environment rather than with high toxin production [6]. Numerous epidemiological studies have shown that *S. haemolyticus*, following *Staphylococcus epidermidis* (*S. epidermidis*), is among the most frequently isolated CoNS from clinical specimens, particularly blood cultures, and represents one of the leading causes of healthcare-associated infections [2,7]. The high frequency of isolation of *S. haemolyticus* in hospital settings is largely attributable to the presence of multidrug-resistant strains and its ability to persist in the medical environment [3,6]. A key virulence factor of *S. haemolyticus*, similar to other CoNS, is its ability to form biofilm, enabling colonization of abiotic surfaces such as intravascular catheters, implants, and prosthetic devices. Growth in the biofilm form promotes chronicity of infections, limits the effectiveness of host immune mechanisms, and significantly reduces the efficacy of antimicrobial therapy [8,9]. Additionally, *S. haemolyticus* is characterized by remarkable genome plasticity, which facilitates the acquisition and accumulation of antimicrobial resistance genes, including methicillin resistance [6]. The significance of *S. haemolyticus* is not limited to human medicine. This species has also been isolated from companion and livestock animals, both from colonization sites and from clinical infections [10,11,12]. Studies conducted in dog and cat populations have confirmed the presence of *S. haemolyticus*, including methicillin-resistant isolates, indicating that companion animals may serve as reservoirs of antimicrobial resistance determinants and highlighting the relevance of this microorganism within the One Health concept [11,12,13]. Despite increasing reports on the presence of *S. haemolyticus* in humans and companion animals, comparative studies simultaneously characterizing isolates from different host species remain scarce [1,2]. Therefore, this study aimed to characterize *S. haemolyticus* isolates recovered from humans, dogs, and cats using phenotypic, molecular, proteomic, and whole-genome approaches and to determine whether antimicrobial resistance, virulence-associated traits, genomic diversity, and pathogenic potential differed according to host species or clinical status.

## 2. Results

### 2.1. Species Identification and Phylogenetic Analysis

A total of 113 archived *Staphylococcus haemolyticus* isolates were re-identified using matrix-assisted laser desorption/ionization time-of-flight mass spectrometry (MALDI-TOF MS). Identification scores of ≥2.000 indicated high-confidence species-level identification. Species identity was further confirmed by detection of the *mvaA* gene, a species-specific marker for *S. haemolyticus* [14].

**Figure 1:**
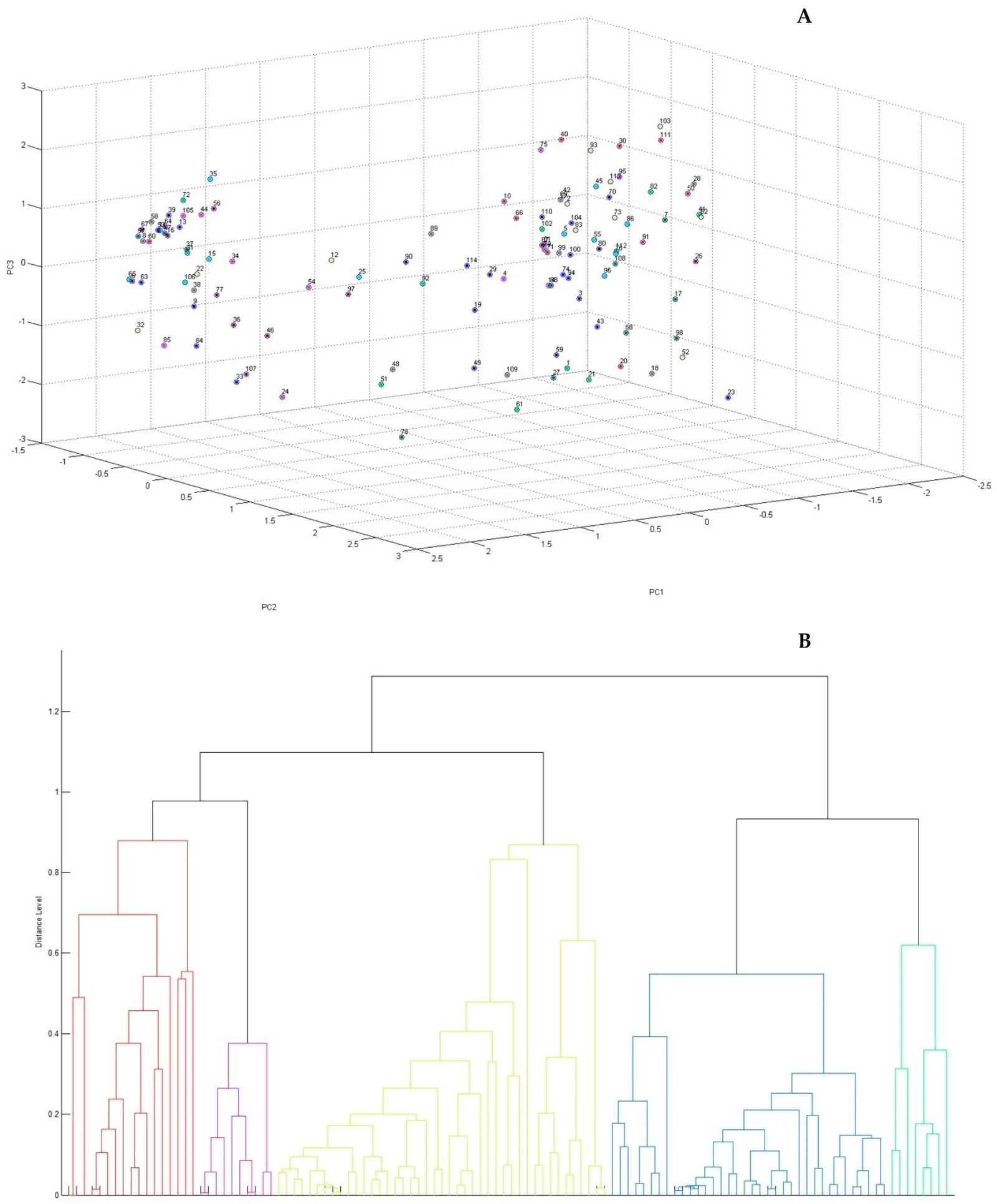
(**A**) Principal component analysis (PCA) of MALDI Biotyper spectra of *S. haemolyticus* isolates and the reference strain. Point colors were assigned automatically by the software and do not represent host species or clinical status. 1. hFe22, 2. hFe1, 3. hCa5, 4. hFe2, 5. hFe11, 6. hFe12, 7. hFe13, 8. sFe23, 9. sFe24, 10. sFe25, 11. sFe26, 12. sFe27, 13. sFe28, 14. hFe3, 15. sFe29, 16. sFe30, 17. sFe31, 18. sFe32, 19. hFe14, 20. hFe15, 21. hFe16, 22. hFe17, 23. hFe18, 24. hFe19, 25. hFe4, 26. hFe20, 27. hFe21, 28. sFe9, 29. sFe10, 30. sFe11, 31. sFe12, 32. sFe13, 33. sFe14, 34. sFe15, 35. sFe16, 36. hFe5, 37. sFe17, 38. sFe18, 39. sFe19, 40. sFe20, 41. sFe21, 42. hFe23, 43. hFe24, 44. hFe25, 45. hFe26, 46. hFe6, 47. sFe7, 48. sFe8, 49. hFe27, 50. hFe28, 51. hFe29, 52. hFe30, 53. hFe31, 54. hFe32, 55. hFe7, 56. sFe33, 57. sFe34, 58. sFe35, 59. sFe36, 60. sFe37, 61. sFe38, 62. sFe39, 63. sFe40, 64. sFe41, 65. sFe42, 66. hFe8, 67. sFe43, 68. sFe44, 69. hFe33, 70. hFe34, 71. hFe35, 72. hFe36, 73. hFe9, 74. hFe10, 75. hHo1, 76. hHo10, 77. hHo11, 78. hHo12, 79. hHo13, 80. hHo14, 81. hHo15, 82. hHo16, 83. hHo17, 84. hHo18, 85. hHo7, 86. hHo8, 87. hHo9, 88. PCM2113 *Staphylococcus haemolyticus*, 89. hHo2, 90. hCa1, 91. hCa2, 92. hCa3, 93. hCa4, 94. hCa6, 95. sFe3, 96. sFe4, 97. hCa7, 98. hCa8, 99. sFe1, 100. sFe2, 101. sHo2, 102. sHo3, 103. sHo4, 104. sHo5, 105. sFe5, 106. sFe6, 107. sFe22, 108. sHo1, 109. hHo3, 110. hHo4, 111. hHo5, 112. hHo6, 113. sHo6, 114. hHo19. (**B**) Hierarchical clustering based on MALDI-TOF MS spectral similarity among *S. haemolyticus* isolates and the reference strain. Colors in the clustering visualization correspond to the clusters identified by the analysis and do not represent host species or clinical status.

MALDI-TOF MS spectra were analysed to assess proteomic similarities among the isolates. Principal component analysis (PCA) followed by hierarchical clustering enabled visualization of relationships based on protein spectral profiles. The resulting dendrogram therefore reflects proteomic similarity and should not be interpreted as a phylogenetic reconstruction. Five major proteomic clusters of different sizes were identified. Three clusters consisted exclusively of feline isolates, whereas the remaining two included isolates from humans, cats, and dogs. However, feline isolates were also present in the mixed clusters, indicating that the observed host-associated proteomic grouping was only partial. Isolates from healthy and clinically affected hosts were intermingled within the clusters, and no clear proteomic segregation according to clinical status was observed. Human and canine isolates did not form distinct host-specific proteomic clusters. Overall, MALDI-TOF MS profiling revealed substantial isolate-level proteomic heterogeneity, with only limited host-associated structuring within the analysed collection. PCA and hierarchical clustering results are shown in Figure 1A and Figure 1B, respectively.

**Figure 2:**
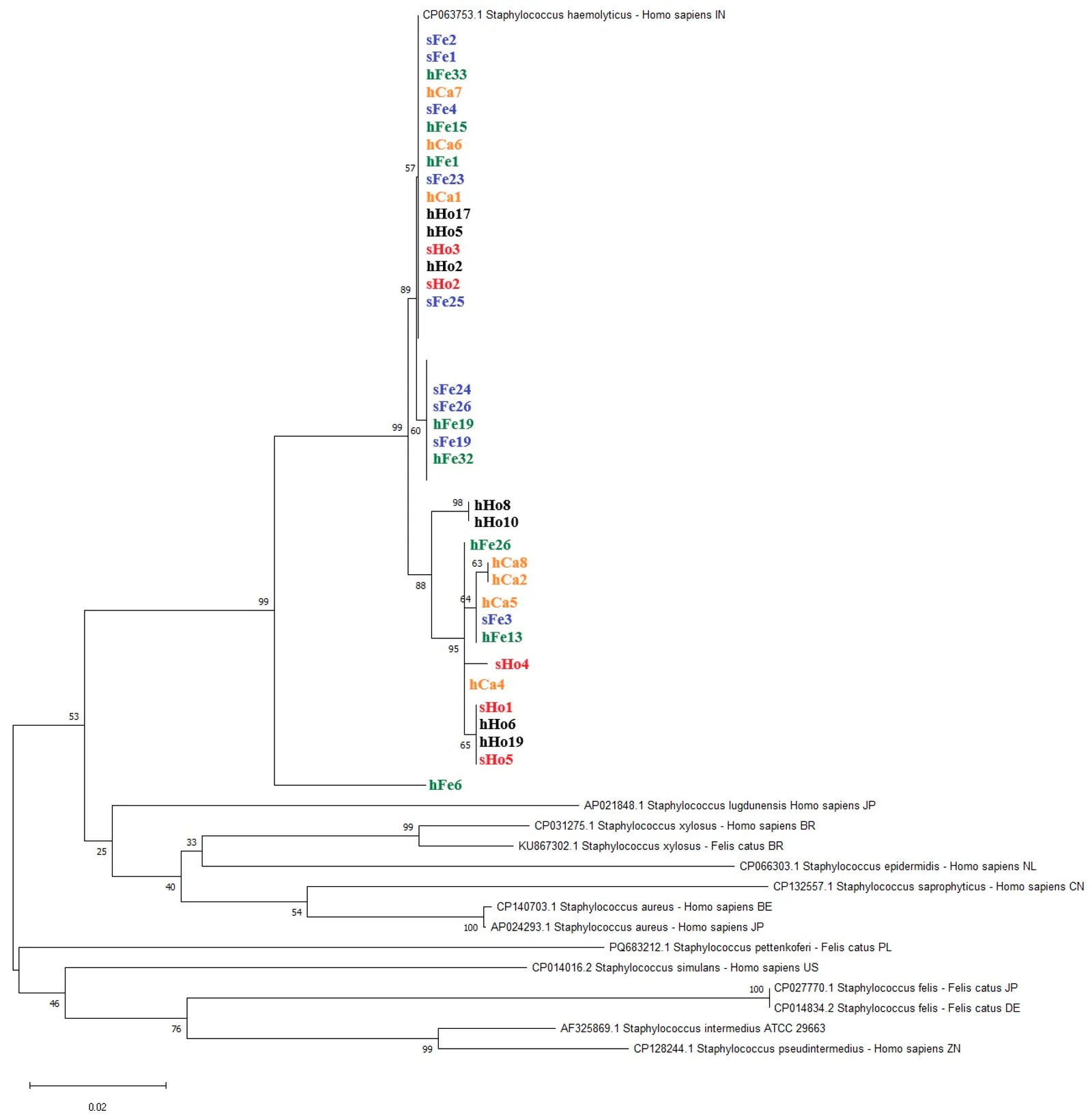
Phylogenetic tree of representative S. haemolyticus isolates based on partial rpoB gene sequences. The tree was inferred using the Neighbor-Joining method with evolutionary distances calculated according to the Tamura–Nei model. The robustness of the tree topology was evaluated by bootstrap analysis with 1000 replicates, and bootstrap values (%) are indicated at the corresponding nodes. Representative isolates were selected to reflect the diversity of the study collection with respect to host species and clinical status. Reference sequences of selected Staphylococcus species retrieved from GenBank were included for comparison. Isolate labels were color-coded according to the study group as follows: black, hHo (clinically healthy humans); red, sHo (clinically affected humans); green, hFe (clinically healthy cats); blue, sFe (clinically affected cats); and orange, hCa (clinically healthy dogs). No isolates from clinically affected dogs were available in the analysed collection.

Phylogenetic analysis based on partial *rpoB* gene sequences further confirmed the species assignment of the examined isolates. All representative isolates clustered with reference *S. haemolyticus* strain (CP063753.1) and were clearly separated from the other *Staphylococcus* species included in the analysis (Figure 2). The corresponding GenBank accession numbers for the *rpoB* sequences analysed in this study are provided in Supplementary Table S1. Several subclusters were observed, with bootstrap support values reaching 99%. Isolates originating from humans, dogs, and cats, as well as from healthy and clinically affected hosts, were distributed throughout the tree, and no clear or consistent segregation according to host species or clinical status was detected within the analysed subset.

### 2.2. Comparative Whole-Genome Analysis

#### 2.2.1. Whole-Genome Sequencing Quality Assessment

Quality assessment was performed for all *Staphylococcus haemolyticus* genome assemblies included in the final WGS dataset. Assemblies were evaluated based on total genome length, number of contigs, N50, GC content, estimated completeness, and contamination. Genome assemblies were considered suitable for downstream comparative genomic analyses when their total length was between 2.2 and 2.8 Mb, estimated completeness was ≥90%, contamination was ≤5%, and the number of contigs was <200. All 35 genome assemblies retained in the final dataset fulfilled these quality criteria. Estimated completeness ranged from 91.66% to 100%, while contamination remained low across the analysed dataset.

The 35 retained genome assemblies were subsequently used for pangenome reconstruction, core-genome phylogenetic analysis, antimicrobial resistance determinant screening, virulence-associated locus analysis, and genotype–phenotype concordance analyses. Detailed genome assembly quality metrics, including genome length, number of contigs, N50, GC content, completeness, and contamination, are provided in Supplementary Table S2.

#### 2.2.2. Multilocus Sequence Typing

Multilocus sequence typing (MLST) was performed using WGS data from the 35 *Staphylococcus haemolyticus* isolates included in the final WGS dataset. Complete seven-locus allelic profiles were obtained for 25/35 isolates. Of these, 24 isolates were assigned to known sequence types represented in the PubMLST database, comprising 12 distinct sequence types (STs). One isolate, hHo10, had a complete seven-locus allelic profile but did not correspond to any known ST and was therefore retained as an unassigned profile.

Among the isolates assigned to known STs, ST52 was the most frequent sequence type, occurring in six isolates. ST48 and ST69 were each identified in four isolates, whereas ST8 was detected in two isolates. The remaining sequence types, including ST1, ST3, ST42, ST46, ST49, ST50, ST65, and ST115, were each represented by a single isolate.

Several sequence types occurred in isolates originating from more than one host species. ST48 was identified among human-, feline-, and canine-derived isolates, whereas ST52 occurred among both human and feline isolates. Other sequence types were restricted to a single host species within the analysed subset. The occurrence of identical STs in isolates originating from different host species was interpreted as evidence of shared clonal backgrounds rather than direct evidence of interspecies transmission. Detailed MLST results, including sequence-type assignment, host origin, clinical status, and sampling site, are provided in Supplementary Table S3.

#### 2.2.3. Pangenome Structure and Core-Genome Phylogeny

Pangenome reconstruction was performed using 35 *Staphylococcus haemolyticus* genome assemblies included in the final comparative genomic dataset. A total of 4581 gene clusters were identified across the analysed genomes. Using a threshold of presence in ≥95% of isolates, 1949 gene clusters were assigned to the core-genome component, comprising 1842 core genes present in 99–100% of genomes and 107 soft-core genes present in 95 – <99% of genomes. The remaining 2632 gene clusters constituted the accessory genome, including 815 shell genes and 1817 cloud genes.

**Figure 3:**
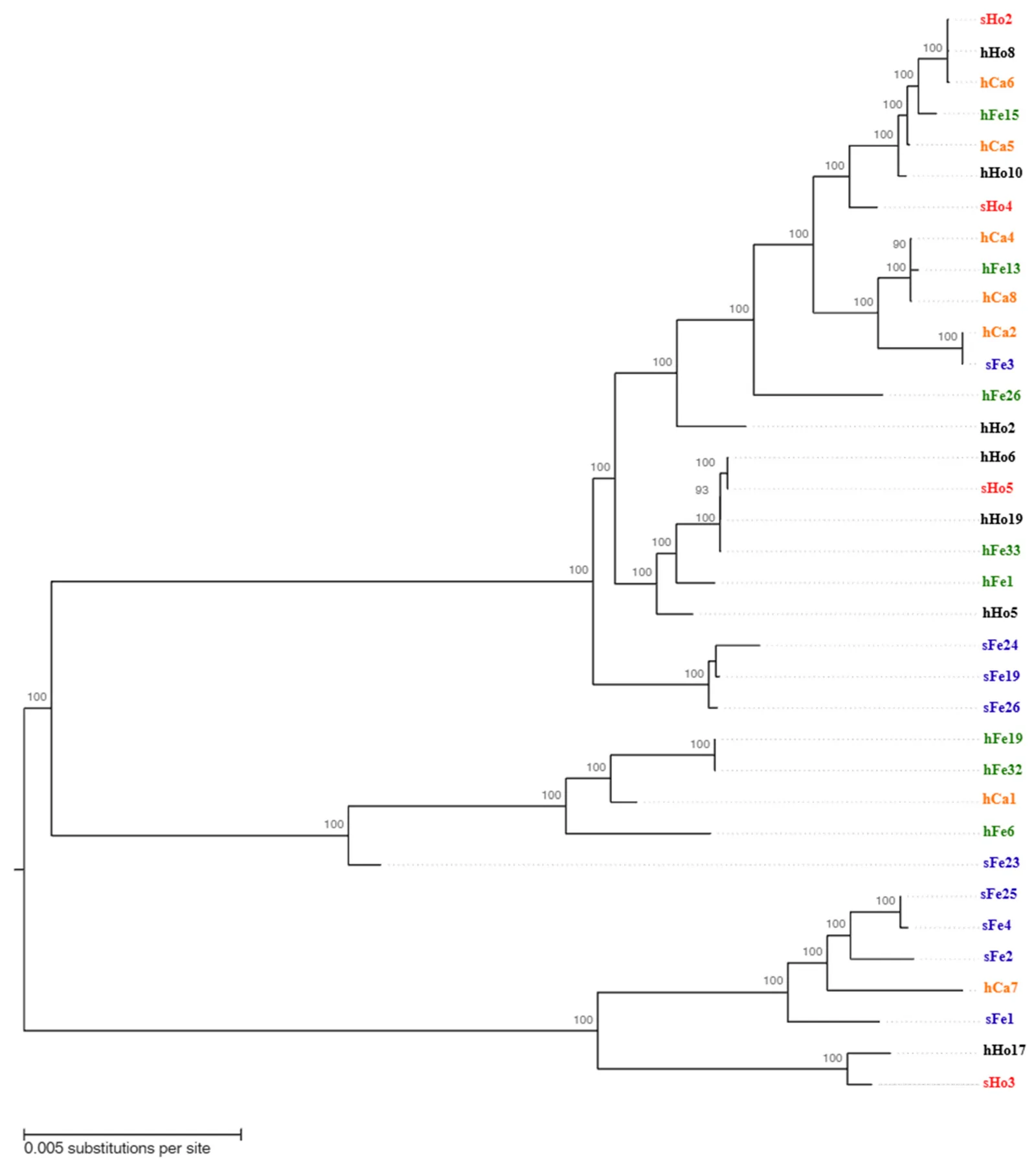
Core-genome maximum-likelihood phylogenetic tree of 35 *Staphylococcus haemolyticus* isolates included in the final WGS dataset. The tree was reconstructed from the concatenated alignment of core genes using IQ-TREE v2.4.0 under the GTR+F+I+R3 substitution model selected by ModelFinder Plus. Branch support was assessed using 1000 SH-aLRT replicates. The scale bar represents 0.005 nucleotide substitutions per site. Isolate labels were color-coded according to host species and clinical status as follows: black, clinically healthy human isolates; red, clinically affected human isolates; green, clinically healthy feline isolates; blue, clinically affected feline isolates; and orange, clinically healthy canine isolates. No clinically affected canine isolates were available in the analysed collection.

Maximum-likelihood phylogenetic analysis based on the concatenated core-gene alignment demonstrated substantial genomic diversity within the analysed *S. haemolyticus* collection (Figure 3). Human-, feline-, and canine-derived isolates were distributed throughout the phylogeny, and no clear segregation according to host species or clinical status was observed within the analysed collection. Several closely related groups contained isolates originating from different host species and, in some cases, from both clinically healthy and clinically affected hosts, suggesting that the major genomic lineages identified within this dataset were not clearly restricted to a particular host species or clinical-status category.

Core-genome relationships were broadly concordant with MLST assignments for several recurrent sequence types. Isolates assigned to ST52 formed a closely related phylogenetic group comprising isolates of both human and feline origin. Similarly, three ST48 isolates of feline and canine origin clustered closely within the phylogeny, while the remaining ST48 isolate was positioned within the same broader phylogenetic lineage but showed a somewhat greater genomic distance. ST69 isolates also showed close phylogenetic relationships, with several members forming a compact cluster, whereas the two ST8 isolates were positioned near one another. These findings indicate that recurrent MLST types generally corresponded to closely related core-genome lineages, although seven-locus MLST did not completely reproduce the relationships resolved by genome-wide sequence analysis.

Pairwise maximum-likelihood genetic distances further supported the presence of closely related isolates originating from different host species. Among the 35 isolates included in the distance analysis, the smallest interspecies distance was observed between feline isolate hFe15 and canine isolate hCa6, with a genetic distance of 0.000001103 substitutions per site. Several closely related human–feline pairs were also identified, including hHo2 and hFe33 (0.000638007 substitutions per site), sHo5 and hFe33 (0.000890360 substitutions per site), and hHo6 and hFe33 (0.000892351 substitutions per site). The closest human–canine pair identified was sHo4 and hCa4, with a distance of 0.001499244 substitutions per site. These findings indicate that closely related genomic backgrounds occurred across host species within the analysed collection; however, in the absence of epidemiological linkage, they should not be interpreted as evidence of direct interspecies transmission. The closest interspecies pairwise genetic distances are summarized in Supplementary Table S4.

#### 2.2.4. Antimicrobial Resistance-Associated Determinants Identified by WGS

Analysis of the 35 genome assemblies included in the final WGS dataset revealed a diverse repertoire of antimicrobial resistance-associated determinants. Among acquired antimicrobial resistance genes, the most frequently detected were *msrA* (20/35; 57.14%), *mphC* (17/35; 48.57%), and β-lactamase determinants of the *blaZ*/PC1 family (17/35; 48.57%). The *ermC* gene was detected in 13/35 isolates (37.14%), whereas *mecA* was present in 12/35 (34.29%). The aminoglycoside resistance determinant *ANT(4′)-Ia* was identified in 11/35 isolates (31.43%), while *aac(6′)-Ie-aph(2″)-Ia* and *tetK* were detected in 9/35 (25.71%) and 8/35 (22.86%) isolates, respectively.

Additional acquired resistance determinants occurred at lower frequencies. The aminoglycoside resistance genes *aad(6)* and *APH(3′)-IIIa*, together with *SAT-4*, were each detected in 7/35 isolates (20.00%). *fusB* and *vgaALC* were each present in 6/35 isolates (17.14%), whereas *tetL* was identified in 5/35 isolates (14.29%). Less frequent determinants included *dfrG* and *fusC* (2/35; 5.71% each), while *mupA* and *vgaA* were each detected in a single isolate (1/35; 2.86%). *tetM* was not detected in any of the analysed genomes.

In addition to acquired resistance genes, several broadly distributed chromosomal genes associated with intrinsic resistance, multidrug efflux, or regulation of antimicrobial susceptibility were highly prevalent within the analysed collection. *sdrM*, *norA*, *mgrA*, *dfrC*, *lmrS*, and *arlR* were each detected in 34/35 genomes (97.14%), whereas *sepA* was present in 33/35 (94.29%). Other frequently identified resistance-associated genes included *arlS* (29/35; 82.86%), *norC* (28/35; 80.00%), and *mepA* (24/35; 68.57%). These genes were considered separately from acquired antimicrobial resistance determinants because they represent broadly distributed intrinsic or resistance-associated functions.

Genes associated with reduced susceptibility to antiseptic or disinfectant compounds were also identified. *qacA* was detected in 23/35 isolates (65.71%), whereas *qacH* was present in 8/35 isolates (22.86%). Detailed isolate-level results of the WGS-based antimicrobial resistance analysis are provided in Supplementary Table S5.

#### 2.2.5. Distribution of WGS-Detected Acquired Antimicrobial Resistance Determinants According to Host Origin and Clinical Status

The distribution of acquired antimicrobial resistance determinants was compared according to host species and clinical status among the 35 *Staphylococcus haemolyticus* genomes included in the final WGS dataset. The analysed subset comprised 11 human, 7 canine, and 17 feline isolates, whereas 22 isolates originated from clinically healthy hosts and 13 from clinically affected hosts. Because of the relatively small and unequal group sizes, exact tests were used. Gene-specific associations with host origin were assessed using the Fisher–Freeman–Halton exact test, whereas comparisons according to clinical status were performed using Fisher’s exact test. Benjamini–Hochberg false discovery rate correction was applied separately to each set of comparisons. Detailed results of the host- and clinical-status-associated comparisons of acquired antimicrobial resistance determinants are provided in Supplementary Table S6.

After correction for multiple comparisons, statistically significant host-associated differences were detected for *mecA* and β-lactamase determinants of the *blaZ*/PC1 family in the primary analysis of 35 genomes. *mecA* was detected in 0/11 human, 2/7 canine, and 10/17 feline isolates (*p* = 0.0031; *q* = 0.0324), whereas *blaZ*/PC1-family determinants were detected in 1/11 human, 4/7 canine, and 12/17 feline isolates (*p* = 0.0036; *q* = 0.0324). Within the analysed subset, both determinants were therefore more frequently detected among animal-derived isolates, particularly those of feline origin.

To assess the potential effect of non-independence arising from multiple isolates obtained from the same individual host, the host- and clinical-status-associated analyses were repeated in a sensitivity-analysis subset comprising one isolate per host (n = 33; 11 human, 6 canine, and 16 feline isolates; 21 from clinically healthy and 12 from clinically affected hosts). In this analysis, the host-associated difference for *mecA* remained statistically significant after FDR correction: *mecA* was detected in 0/11 human, 1/6 canine, and 10/16 feline isolates (*p* = 0.0015; *q* = 0.0269). In contrast, the association observed for *blaZ*/PC1-family determinants in the primary analysis was attenuated after restriction to one isolate per host. These determinants were detected in 1/11 human, 3/6 canine, and 11/16 feline isolates (*p* = 0.0064), but the association no longer reached statistical significance after FDR correction (*q* = 0.0577).

A nominal association with host origin was observed for *dfrG* in the primary analysis (*p* = 0.0353), which was detected in two canine isolates and was absent from the human and feline isolates; however, this association did not remain significant after FDR correction (*q* = 0.2118). No other acquired antimicrobial resistance determinant showed a statistically significant association with host species after multiple-testing correction.

No acquired antimicrobial resistance determinant was significantly associated with clinical status after FDR correction in the primary analysis. Although *ermC* was more frequently detected among isolates from clinically affected hosts (8/13) than among those from clinically healthy hosts (5/22), the nominal association (*p* = 0.0328) did not remain significant after FDR adjustment (*q* = 0.2358). This conclusion was unchanged in the one-isolate-per-host sensitivity analysis, in which no acquired resistance determinant remained significantly associated with clinical status after FDR correction.

Overall, the sensitivity analysis confirmed that the host-associated signal for *mecA* was robust to restriction to one isolate per individual host, whereas the association involving *blaZ*/PC1-family determinants was sensitive to the treatment of non-independent isolates. Given the relatively small WGS subset, unequal host-group sizes, and the absence of clinically affected canine isolates, these findings should be regarded as exploratory and interpreted with appropriate caution.

#### 2.2.6. Genotype–Phenotype Concordance Based on WGS

Genotype–phenotype concordance was assessed among the 35 *Staphylococcus haemolyticus* isolates included in the final WGS dataset by comparing WGS-detected acquired antimicrobial resistance determinants with phenotypic susceptibility classifications obtained by disk diffusion and MIC testing. Disk-diffusion and MIC-based phenotypes were analysed independently where both methods were available. For oxacillin specifically, MIC-based classification was considered the primary susceptibility phenotype, whereas disk-diffusion classification was retained as an operational comparative phenotype.

Overall concordance varied considerably according to the antimicrobial class and phenotypic testing method. For oxacillin, using MIC-based classification as the primary susceptibility phenotype, the presence of *mecA* showed only slight agreement (Cohen’s κ = 0.095; 23/35 concordant isolates, 65.71% agreement). Discordance was predominantly attributable to *mecA*-positive isolates classified as phenotypically susceptible (10 isolates), compared with two *mecA*-negative isolates showing phenotypic resistance (McNemar p = 0.039). In a separate secondary analysis, *mecA* showed substantial agreement with the operational disk-diffusion classification (κ = 0.689; 30/35 concordant isolates, 85.71% agreement), with no significant directional discordance (McNemar *p* = 1.000). The higher concordance observed for the operational disk-diffusion classification was interpreted solely as a method-dependent difference in genotype–phenotype agreement and not as evidence that disk diffusion is preferable to, equivalent to, or interchangeable with MIC for oxacillin susceptibility assessment in *S. haemolyticus*.

For erythromycin, the combined presence of *ermC*, *msrA*, or *mphC* showed fair agreement with disk-diffusion results (κ = 0.250; 65.71% agreement), but almost perfect agreement with MIC-based classification (κ = 0.821; 94.29% agreement). Disk-diffusion discordance was predominantly directed towards genotype-positive/phenotype-susceptible isolates (10 vs. 2 discordant isolates; McNemar *p* = 0.039), whereas the MIC-based comparison showed balanced discordance (one isolate in each direction; McNemar *p* = 1.000).

For clindamycin, the combined presence of *ermC*, *vgaA*, or *vgaALC* showed substantial agreement with both disk-diffusion (κ = 0.659; 82.86% agreement) and MIC-based classifications (κ = 0.604; 80.00% agreement). The MIC-based comparison nevertheless showed significant directional discordance, with seven genotype-positive isolates remaining phenotypically susceptible and no phenotype-resistant/genotype-negative isolates (McNemar *p* = 0.016).

Tetracycline resistance showed high genotype–phenotype concordance. The combined presence of *tetK* or *tetL* showed almost perfect agreement with disk-diffusion classification (κ = 0.813; 91.43% agreement) and substantial agreement with MIC-based classification (κ = 0.655; 82.86% agreement). No significant directional discordance was detected for either phenotypic method.

Among additional disk-diffusion comparisons, *mupA* showed complete concordance with mupirocin resistance (κ = 1.000; 35/35 concordant isolates), although this finding was based on a single *mupA*-positive isolate. The aminoglycoside resistance determinant *aac(6′)-Ie-aph(2″)-Ia* showed substantial agreement with gentamicin resistance (κ = 0.650; 88.57% agreement). The combined presence of *fusB* or *fusC* showed only fair agreement with fusidic acid resistance (κ = 0.340; 82.86% overall agreement), with discordance resulting exclusively from genotype-positive/phenotype-susceptible isolates (McNemar *p* = 0.031).

Detailed genotype–phenotype concordance results, including concordant and discordant categories, Cohen’s κ values, McNemar test results, and the direction of discordance, are provided in Supplementary Table S7.

#### 2.2.7. Concordance Between PCR- and WGS-Based Detection of Antimicrobial Resistance Genes

The concordance between PCR- and WGS-based detection of antimicrobial resistance genes was assessed for the 35 *Staphylococcus haemolyticus* isolates included in the final WGS antimicrobial resistance dataset. Agreement varied among the analysed resistance determinants.

High concordance was observed for several genes. For *blaZ*, PCR detected the gene in 19/35 isolates (54.3%), whereas WGS identified a *blaZ*/PC1-family β-lactamase determinant in 17/35 isolates (48.6%). Concordant positive results were obtained for 17 isolates, whereas two isolates were PCR-positive/WGS-negative and no isolate was PCR-negative/WGS-positive. Overall agreement was 94.3%, with almost perfect agreement beyond chance (Cohen’s κ = 0.886). The distribution of discordant results was not significantly asymmetric (McNemar *p* = 0.500).

For *mecA*, both PCR and WGS detected the gene in 12/35 isolates (34.3%). Eleven isolates were positive by both methods, one was PCR-positive/WGS-negative, and one was PCR-negative/WGS-positive. Overall agreement was 94.3%, with almost perfect agreement (κ = 0.873; McNemar *p* = 1.000).

Similarly high concordance was observed for *aac(6′)-Ie-aph(2″)-Ia*, which was detected by PCR in 8/35 isolates (22.9%) and by WGS in 9/35 isolates (25.7%). Eight isolates were positive by both methods, with only one PCR-negative/WGS-positive result. Overall agreement reached 97.1%, with almost perfect agreement (κ = 0.922).

The most pronounced discrepancy was observed for *ermC*. PCR detected *ermC* in 3/35 isolates (8.6%), whereas WGS identified the gene in 13/35 isolates (37.1%). All three PCR-positive isolates were also WGS-positive, while 10 additional isolates were PCR-negative/WGS-positive. Overall agreement was 71.4%, with only fair agreement beyond chance (κ = 0.274). The discordance was significantly asymmetric (McNemar *p* = 0.002), indicating more frequent detection of *ermC* by WGS than by PCR.

For tetracycline resistance determinants, agreement was also high. *tetK* was detected by PCR in 7/35 isolates and by WGS in 8/35 isolates, with seven concordant positive results and one PCR-negative/WGS-positive isolate, resulting in 97.1% overall agreement and almost perfect agreement (κ = 0.915). For *tetL*, PCR detected the gene in 7/35 isolates compared with 5/35 by WGS. Five isolates were concordantly positive, whereas two were PCR-positive/WGS-negative, resulting in 94.3% agreement and substantial agreement beyond chance (κ = 0.800).

Complete concordance was observed for *fusB* and *mupA*. *fusB* was detected by both methods in 6/35 isolates, yielding 100% agreement and κ = 1.000. *mupA* was detected by both PCR and WGS in a single isolate, also resulting in 100% agreement and κ = 1.000; however, this result should be interpreted cautiously because of the very low prevalence of the determinant. *tetM* was absent by both methods in all 35 isolates, resulting in complete negative concordance; consequently, Cohen’s κ could not be meaningfully estimated because of the absence of variation.

Overall, PCR and WGS showed high concordance for most analysed antimicrobial resistance genes, whereas *ermC* represented the principal source of disagreement, being considerably more frequently detected by WGS. Detailed gene-specific PCR–WGS concordance data, including concordant and discordant categories, Cohen’s κ values, and McNemar test results, are presented in Supplementary Table S8.

#### 2.2.8. VFDB-Associated Loci Identified by WGS

VFDB-associated loci were evaluated among the 35 *Staphylococcus haemolyticus* genomes included in the final WGS dataset using the Virulence Factor Database (VFDB). Of the 34 loci included in the resulting VFDB-based presence/absence matrix, 23 were detected in at least one genome. Detailed isolate-level distributions of VFDB-associated loci are provided in Supplementary Table S9. Three broadly distributed loci, *tufA*, *clpC*, and *clpP*, were detected in all 35 genomes (100%). These loci were treated as VFDB-associated stress- and adaptation-related determinants rather than as experimentally confirmed virulence factors.

Capsule-associated loci represented the most variably distributed group. *cap8D* was detected in 19/35 isolates (54.29%), whereas *cap8E*, *cap8F*, and *cap8G* were each present in 11/35 isolates (31.43%). *cap8B* was identified in 8/35 isolates (22.86%), while *cap8L*, *cap8M*, *cap9O*, and *cap8P* were each detected in 7/35 isolates (20.00%). The *capC* locus was present in 6/35 isolates (17.14%), whereas *cap8J* and *cap8K* were each detected in a single genome (2.86%).

Loci associated in VFDB with the type VII secretion system were less frequent. *esaG* was identified in 8/35 genomes (22.86%), whereas *esaB*, *esaE*, *essA*, *essB*, *essC*, *esxA*, and *esxB* were each detected in 2/35 genomes (5.71%). Their detection indicates the presence of sequences annotated in VFDB as components of the type VII secretion system but does not by itself demonstrate functional activity or contribution to virulence in the analysed isolates.

The distribution of variable VFDB-associated loci was subsequently compared to host origin and clinical status. Twenty loci showing variation within the analysed collection were included in the statistical analysis. Host-related differences were evaluated using the Fisher–Freeman–Halton exact test, whereas clinical-status comparisons were performed using two-sided Fisher’s exact tests. Benjamini–Hochberg false discovery rate correction was applied separately to both sets of comparisons.

Nominal host-related differences were observed for several loci. *cap8B* was detected in 1/11 human, 5/7 canine, and 2/17 feline isolates (unadjusted *p* = 0.0060), whereas *cap8D* was detected in 4/11 human, 7/7 canine, and 8/17 feline isolates (unadjusted *p* = 0.0220). Several VFDB-associated type VII secretion system loci also showed nominal host-related differences (unadjusted *p* = 0.0353). However, none of these associations remained statistically significant after FDR correction in the primary analysis of 35 genomes (lowest *q* = 0.0784).

To assess the potential effect of non-independence resulting from multiple isolates obtained from the same individual host, the host- and clinical-status-associated analyses were repeated in the sensitivity-analysis subset restricted to one isolate per host (n = 33; 11 human, 6 canine, and 16 feline isolates). In this analysis, the host-associated difference for *cap8B* became statistically significant after FDR correction (*p* = 0.00058; *q* = 0.0115). This result indicates that the host-associated signal for *cap8B* was sensitive to the analytical treatment of repeated host sampling. Detailed results of the one-isolate-per-host sensitivity analysis of VFDB-associated loci are provided in Supplementary Table S9a.

No statistically significant association between the distribution of VFDB-associated loci and clinical status was identified after correction for multiple comparisons. This conclusion remained unchanged in the one-isolate-per-host sensitivity analysis. Overall, the VFDB-associated locus repertoire showed substantial isolate-level variation, and the sensitivity analysis indicated that some host-associated signals may depend on dataset structure and the treatment of non-independent isolates. These findings should therefore be interpreted cautiously because of the relatively small and unequal host groups and the absence of clinically affected canine isolates. Moreover, the presence of VFDB-associated loci alone does not demonstrate their expression, functional activity, or mechanistic contribution to virulence-related phenotypes.

### 2.3. Antimicrobial Susceptibility Phenotyping

#### 2.3.1. Antimicrobial Susceptibility Testing by Disk Diffusion

Antimicrobial susceptibility testing of the 113 *S. haemolyticus* isolates revealed that 27/113 (23.89%) were susceptible to all antimicrobial agents tested by disk diffusion. A total of 40 distinct disk-diffusion-based phenotypic resistance profiles were identified. For the purpose of descriptive resistance-profile analysis, oxacillin disk-diffusion categories were retained as operational phenotypic categories, allowing the original disk-diffusion patterns to be presented consistently across the full antimicrobial panel. These oxacillin categories should not be interpreted as CLSI-validated clinical susceptibility classifications. Of these, 20 were unique profiles represented by a single isolate, whereas the remaining 20 were recurrent. The most common resistance profile comprised resistance to ampicillin, penicillin, clindamycin, erythromycin, and tetracycline (AMP–P–DA–E–TET) and was identified in 9/113 (7.96%) isolates. The second most frequent profile additionally included resistance to amoxicillin/clavulanic acid (AMP–AMC–P–DA–E–TET) and occurred in 8/113 (7.08%) isolates. Detailed information on all phenotypic resistance profiles is provided in Supplementary Table S10.

When MIC-based oxacillin classification was used as the primary oxacillin susceptibility phenotype, 51/113 (45.13%) isolates fulfilled the definition of multidrug resistance (MDR), defined as resistance to at least one antimicrobial agent in three or more antimicrobial classes [15]. Isolates that were *mecA*-positive but classified as oxacillin-susceptible by MIC were retained as phenotypically susceptible for the purposes of MDR classification. Because resistance genes were assessed solely on a presence/absence basis and gene expression was not evaluated, *mecA* carriage alone was not used to override the MIC-based phenotypic classification. Accordingly, the reported MDR frequency remained 51/113 (45.13%).

The highest resistance rate was observed for erythromycin (71/113; 62.83%), followed by ampicillin (65/113; 57.52%), penicillin (63/113; 55.75%), clindamycin (51/113; 45.13%), tetracycline (50/113; 44.25%), and amoxicillin/clavulanic acid (32/113; 28.32%). For oxacillin, 23/113 (20.35%) isolates were assigned to the resistant category according to the operational disk-diffusion criterion applied in this study. Because oxacillin disk diffusion is not a CLSI-supported primary susceptibility method for *S. haemolyticus*, this classification was retained for descriptive and comparative purposes and should not be interpreted as a definitive CLSI-validated susceptibility classification. Resistance rates for ciprofloxacin, marbofloxacin, and gentamicin were 14/113 (12.39%), 12/113 (10.62%), and 12/113 (10.62%), respectively. Lower resistance rates were recorded for fusidic acid (6/113; 5.31%), trimethoprim/sulfamethoxazole (4/113; 3.54%), mupirocin (3/113; 2.65%), tobramycin (3/113; 2.65%), and chloramphenicol (2/113; 1.77%). No resistance was detected to linezolid, rifampicin, or tigecycline.

Differences in antimicrobial resistance frequencies were observed among the analysed host groups. The distribution of resistant isolates varied depending on both the antimicrobial agent and host group. Oxacillin disk-diffusion categories were interpreted as operational phenotypic categories only, whereas MIC-based oxacillin classification was evaluated separately as the primary susceptibility phenotype. Detailed resistance frequencies for each antimicrobial agent and host group are presented in Table 1.

#### 2.3.2. Minimal Inhibitory Concentration and Minimal Bactericidal Concentration for Selected Antimicrobials

Among the 113 analysed *S. haemolyticus* isolates, no resistance to teicoplanin or vancomycin was observed, indicating complete susceptibility to both glycopeptide antimicrobial agents. In contrast, the highest overall resistance rate was recorded for erythromycin (85/113; 75.22%), followed by tetracycline (58/113; 51.33%), clindamycin (29/113; 25.66%), and MIC-based oxacillin resistance (12/113; 10.62%).

Due to the unequal distribution of isolates among the analysed host groups, comparisons between specific groups should be interpreted with caution. Nevertheless, resistance frequencies varied descriptively among host groups depending on the antimicrobial agent tested. The highest erythromycin resistance was observed among clinically affected feline isolates (39/44; 88.64%), whereas tetracycline resistance was also most frequent in this group (31/44; 70.45%). Clindamycin resistance was most prevalent among canine isolates (4/8; 50.00%), while MIC-based oxacillin resistance remained relatively uncommon in all groups, ranging from 0.00% to 16.67%. No resistance to teicoplanin or vancomycin was detected in any host group.

The relationship between the minimum inhibitory concentration (MIC) and the experimentally determined bactericidal endpoint was further evaluated using the log_2_(MBC/MIC) ratio. Considerable variability was observed across antimicrobial agents and individual isolates. In some cases, the bactericidal endpoint occurred at the same or only slightly higher antimicrobial concentration than the MIC, whereas in others substantially higher concentrations were required. This variability was observed independently of the MIC susceptibility category.

A comprehensive heatmap illustrating the log_2_(MBC/MIC) ratios for all analysed isolates, together with the corresponding MIC values and susceptibility classifications, is presented in Supplementary Table S11A–C.

The analysis of antimicrobial resistance genes revealed that *blaZ* was the most frequently detected determinant, being present in 61/113 isolates (53.98%). The second most prevalent gene was *tetL*, detected in 38/113 isolates (33.63%), followed by *mecA* (19/113; 16.81%), *aac(6′)-Ie-aph(2″)-Ia* (15/113; 13.27%), *tetK* (13/113; 11.50%), and *fusB* (12/113; 10.62%). The *ermC* and *mupA* genes were each detected in only three isolates (2.65%), whereas *tetM* was identified in a single isolate (0.88%). The *tetO*, *mecC*, *ermA*, *ermB*, *vanA*, and *vanB* genes were not detected in any isolate. The distribution of resistance genes differed among the analysed host groups. The *blaZ* gene predominated in feline isolates, being detected in 34/44 isolates from clinically affected cats (77.27%) and 19/36 isolates from healthy cats (52.78%), whereas it was less common in isolates from healthy dogs (4/8; 50.00%) and healthy humans (4/19; 21.05%) and was absent from isolates obtained from clinically affected humans. Similarly, *mecA* was most frequently detected in isolates from clinically affected cats (12/44; 27.27%), followed by healthy dogs (2/8; 25.00%), healthy cats (4/36; 11.11%), and healthy humans (1/19; 5.26%). The *tetL* gene was predominantly detected in feline isolates, occurring in 24/44 isolates from clinically affected cats (54.55%) and 12/36 isolates from healthy cats (33.33%), whereas it was detected in only 2/19 isolates from healthy humans (10.53%) and was absent from isolates obtained from dogs and clinically affected humans. In contrast, *fusB* was most frequently detected in isolates from healthy dogs (4/8; 50.00%) and clinically affected humans (2/6; 33.33%), while the aminoglycoside resistance gene *aac(6′)-Ie-aph(2″)-Ia* was detected most frequently in healthy dogs (3/8; 37.50%) and was also present in 8/44 isolates from clinically affected cats (18.18%). These descriptive differences should be interpreted cautiously because of the unequal sample sizes among the analysed groups. Detailed results are presented in Table 2. Among the analysed isolates, 33/113 (29.20%) did not harbor any of the antimicrobial resistance genes investigated. The most common genotypic resistance profile was *blaZ + tetL*, identified in 19/113 isolates (16.81%), followed by *blaZ* alone in 13/113 isolates (11.50%). The profiles comprising *tetL* alone and *blaZ + mecA + tetL* were each identified in 8/113 isolates (7.08%). All remaining genotypic resistance profiles occurred in fewer than 5% of isolates and are presented in Supplementary Table S12.

### 2.4. Analysis of Bacterial Growth Kinetics and Biofilm Formation

Growth kinetics were analysed for 17 representative *S. haemolyticus* isolates (hHo2, hHo6, hHo19, sHo5, hCa1, hCa4, hCa5, hCa6, hCa8, hFe1, hFe13, hFe18, hFe32, hFe33, sFe1, sFe2, and sFe4), together with three reference strains (*S. haemolyticus* PCM 2113, *S. epidermidis* PCM 1869, and *Staphylococcus aureus* ATCC 43300), following incubation at 37 °C, 38 °C, and 39 °C. Growth curves obtained at the three incubation temperatures for the analysed isolates and reference strains are presented in Supplementary Figure S1A–R. The specific growth rate (μ, h^−1^) and corresponding doubling time (tD, h), calculated for each isolate and reference strain at each temperature, are provided in Supplementary Table S13, whereas temperature-dependent differences in doubling time are visualized in Supplementary Figure S2.

The analysed strains exhibited substantial heterogeneity in their growth response to increasing temperature. For descriptive purposes, the observed responses were grouped post hoc into five temperature-dependent growth patterns based on the direction and shape of changes in specific growth rate across 37 °C, 38 °C, and 39 °C. These categories were not predefined before data analysis and were not derived from formal statistical clustering or threshold criteria. The five descriptive patterns comprised a gradual decrease in growth rate with increasing temperature, a gradual increase, a maximum growth rate at 38 °C, a decrease at 38 °C followed by recovery at 39 °C, and a temperature-independent pattern characterized by only limited variation across the three temperatures. The most frequently observed pattern was a gradual decrease in growth rate with increasing temperature. The distribution of strains among these descriptive growth patterns and their relationship with biofilm formation are presented in Table 3.

Comparison of temperature-dependent growth kinetics and biofilm formation showed that changes in planktonic growth were not consistently associated with changes in biofilm-forming capacity. Despite considerable variation in growth responses to increasing temperature, the strongest biofilm phenotype was most frequently observed at 39 °C. Overall, the temperature supporting maximal planktonic growth was strain-dependent and did not necessarily correspond to the temperature associated with the highest biofilm-forming capacity.

Biofilm-forming capacity was strongly temperature-dependent. At 37 °C, 50/113 isolates (44.25%) were classified as weak, 50/113 (44.25%) as moderate, and 13/113 (11.50%) as strong biofilm producers. At 38 °C, 53/113 isolates (46.90%) were classified as weak, 52/113 (46.02%) as moderate, and 8/113 (7.08%) as strong producers. In contrast, at 39 °C, 81/113 isolates (71.68%) were classified as strong biofilm producers, whereas the remaining 32/113 (28.32%) were classified as moderate producers. No isolate was classified as a non-biofilm producer at any of the three incubation temperatures.

Quantitative analysis showed a marked increase in biofilm production at 39 °C. Mean OD_570_ values were 0.515 ± 0.399 at 37 °C, 0.454 ± 0.317 at 38 °C, and 1.118 ± 0.561 at 39 °C. Incubation temperature had a significant overall effect on quantitative biofilm production (likelihood-ratio χ^2^ = 234.42, df = 2, *p* < 0.001). Holm-adjusted pairwise comparisons showed significantly greater biofilm production at 39 °C than at 37 °C (estimated mean difference = 0.603 OD_570_, adjusted *p* < 0.001) and 38 °C (estimated mean difference = 0.664 OD_570_, adjusted *p* < 0.001), whereas the difference between 37 °C and 38 °C was not statistically significant (estimated mean difference = −0.061 OD_570_, adjusted *p* = 0.094).

No significant overall effect of host species (likelihood-ratio χ^2^ = 0.071, df = 2, *p* = 0.965) or clinical status (likelihood-ratio χ^2^ = 0.200, df = 1, *p* = 0.655) was detected. Furthermore, neither the temperature × host species interaction (likelihood-ratio χ^2^ = 1.03, df = 4, *p* = 0.905) nor the temperature × clinical status interaction (likelihood-ratio χ^2^ = 0.105, df = 2, *p* = 0.949) was statistically significant. Thus, within the analysed collection, there was no statistically supported evidence that the temperature-dependent biofilm response differed among host species or clinical-status groups.

To assess the potential influence of repeated sampling from the same individual host, the analysis was repeated in a sensitivity-analysis subset comprising one isolate per host (84 isolates representing 84 unique hosts). The significant overall effect of incubation temperature was retained (likelihood-ratio χ^2^ = 106.26, df = 2, *p* < 0.001), whereas no significant effects of host species (χ^2^ = 0.034, df = 2, *p* = 0.983) or clinical status (χ^2^ = 0.398, df = 1, *p* = 0.528) were detected. Neither the temperature × host species interaction (χ^2^ = 4.527, df = 4, *p* = 0.339) nor the temperature × clinical status interaction (χ^2^ = 0.166, df = 2, *p* = 0.920) was statistically significant. Holm-adjusted pairwise comparisons again showed significantly greater biofilm production at 39 °C than at 37 °C and 38 °C (adjusted *p* < 0.001 for both comparisons). In the one-isolate-per-host subset, the difference between 37 °C and 38 °C reached statistical significance (estimated mean difference = −0.083 OD_570_, adjusted *p* = 0.040). Thus, the principal findings of a pronounced increase in biofilm production at 39 °C and the absence of host-species- or clinical-status-associated effects remained unchanged after accounting for repeated sampling from individual hosts.

**Figure 4:**
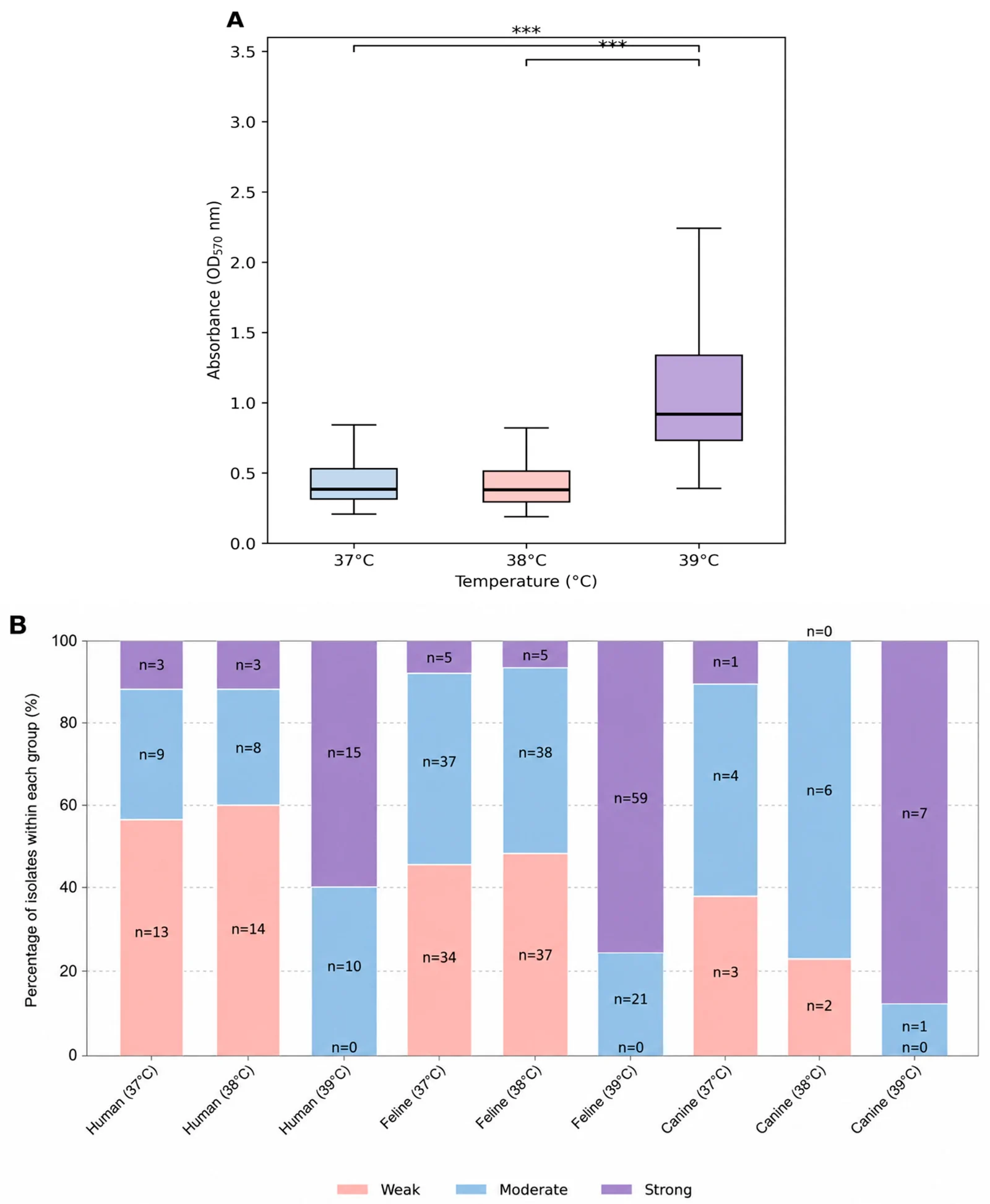
(**A**) Quantitative analysis of biofilm production by *S. haemolyticus* isolates after 24 h incubation at 37 °C, 38 °C, and 39 °C. Biofilm biomass was measured using the crystal violet assay and expressed as OD_570_ values. Box plots represent the distribution of OD_570_ values among 113 isolates; horizontal lines indicate medians, boxes indicate interquartile ranges, and whiskers indicate data variability. Statistical significance was assessed using a linear mixed-effects model with Holm-adjusted pairwise comparisons (*** *p* < 0.001). (**B**) Distribution of biofilm-forming capacity categories (weak, moderate, and strong) among *S. haemolyticus* isolates according to host origin (human, feline, and canine) and incubation temperature (37 °C, 38 °C, and 39 °C). Stacked bars indicate the percentage of isolates within each group, with the number of isolates (*n*) shown within each category.

Quantitative biofilm production and the distribution of biofilm-forming capacity categories according to incubation temperature and host origin are shown in Figure 4A and Figure 4B, respectively, while detailed quantitative data for individual isolates at each incubation temperature are provided in Supplementary Table S14. Results of the one-isolate-per-host sensitivity analysis are summarized in Supplementary Table S14a.

### 2.5. Concordance Between Phenotypic and Genotypic Resistance

Overall, genotype–phenotype concordance varied substantially depending on the resistance determinant and the antimicrobial agent tested. The strongest agreement was observed for selected gene–antimicrobial combinations, whereas several comparisons showed only slight or fair agreement, indicating that the presence of a resistance gene did not consistently predict phenotypic resistance.

Among β-lactam resistance determinants, *blaZ* showed substantial agreement with phenotypic resistance to penicillin and ampicillin (κ = 0.607 and κ = 0.606, respectively). In contrast, agreement with amoxicillin/clavulanic acid was only fair (κ = 0.297), and concordance with oxacillin was limited, particularly when assessed by MIC (κ = 0.017). For the operational disk-diffusion oxacillin classification, agreement was also only fair (κ = 0.256). The *mecA* gene showed variable agreement with β-lactam resistance. Concordance with oxacillin was slight when MIC-based classification was used (κ = 0.073) and moderate when the operational disk-diffusion classification was used (κ = 0.475). Agreement with penicillin, ampicillin, and amoxicillin/clavulanic acid remained slight to fair. For macrolide and lincosamide resistance, *ermC* showed only slight agreement with both erythromycin and clindamycin phenotypes. Sensitivity was very low, whereas specificity remained high, indicating that many phenotypically resistant isolates did not carry *ermC*. Among tetracycline resistance determinants, *tetL* showed the highest concordance with phenotypic resistance, with fair agreement based on MIC (κ = 0.374) and moderate agreement based on disk diffusion (κ = 0.451). In contrast, *tetM* and *tetK* showed only slight agreement with tetracycline resistance. Complete concordance was observed between *mupA* and phenotypic mupirocin resistance, with no discordant isolates identified. However, this finding should be interpreted cautiously because only three isolates were *mupA*-positive. The *aac(6′)-Ie-aph(2″)-Ia* gene showed substantial agreement with gentamicin resistance (κ = 0.622), whereas *fusB* showed only slight agreement with fusidic acid resistance (κ = 0.163). Concordance analysis was not applicable for *mecC*, *ermA*, *ermB*, *vanA*, *vanB*, and *tetO*, as these genes were not detected in the analysed isolates. Detailed genotype–phenotype concordance results, including true-positive, true-negative, false-positive and false-negative counts, sensitivity, specificity, Cohen’s κ with 95% confidence intervals, and McNemar test results, are provided in Supplementary Table S15A and S15B.

### 2.6. Pathogenic Potential Assessment Using the Galleria mellonella Infection Model

**Figure 5:**
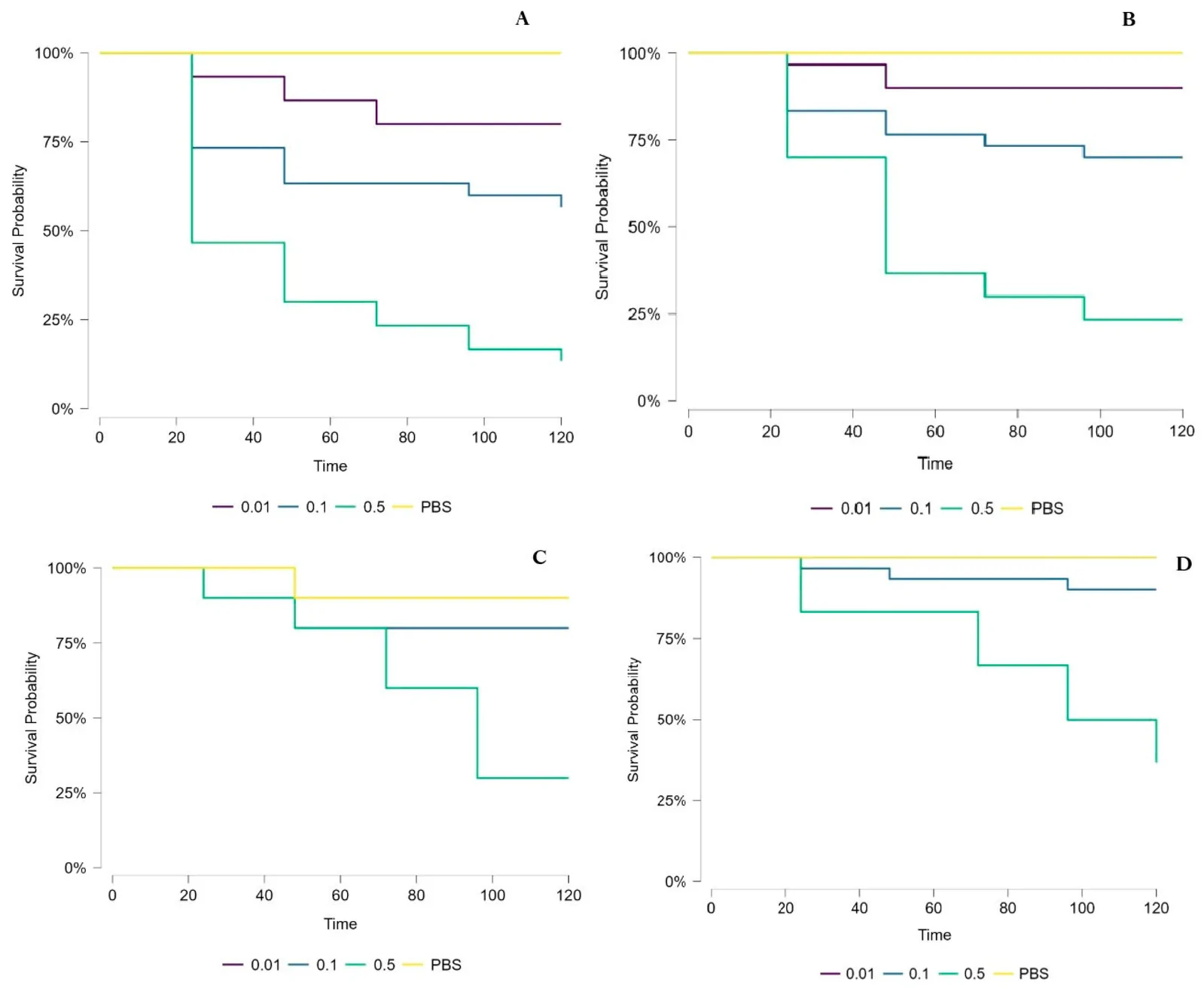

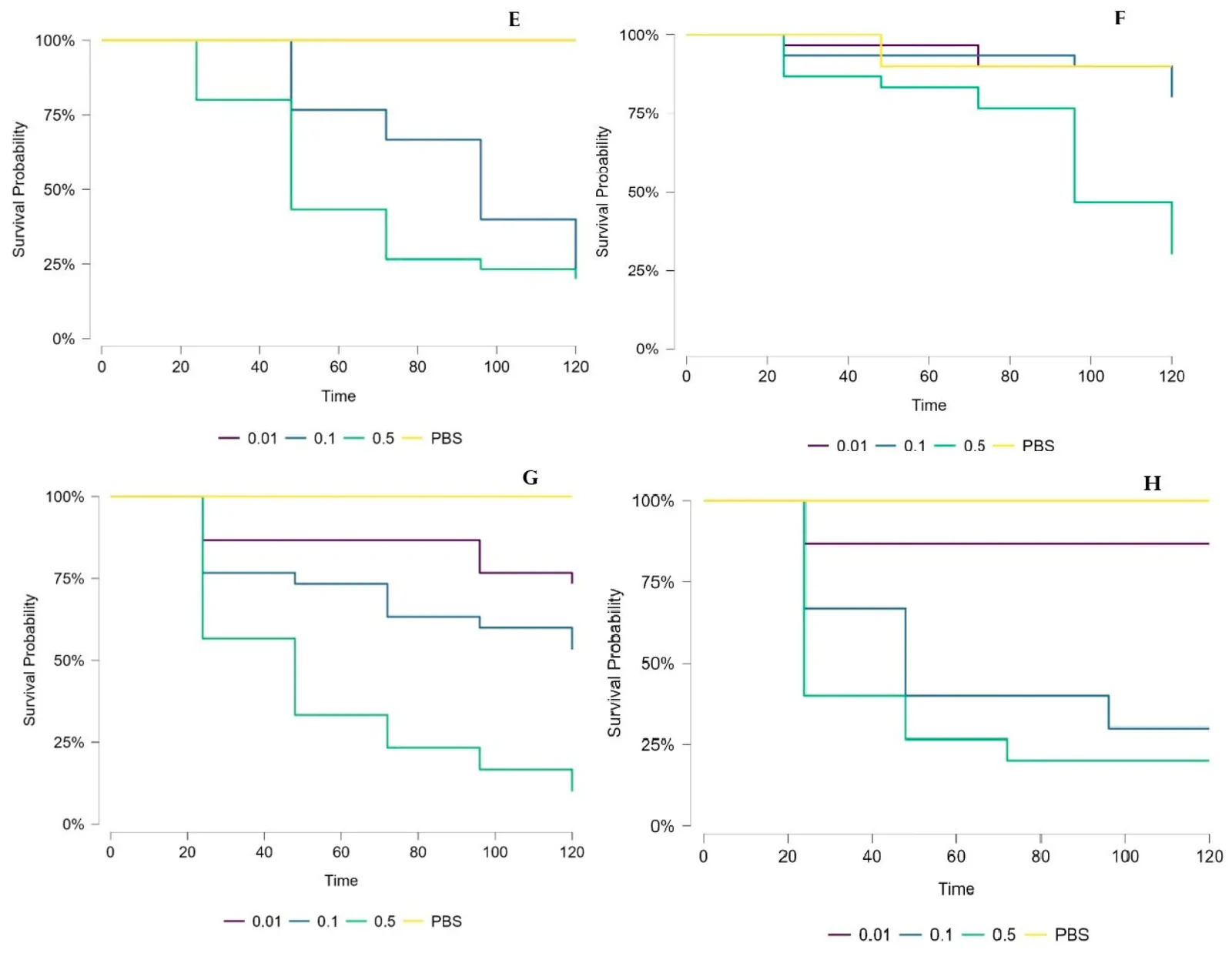
(**A**–**H**). Kaplan–Meier survival curves of *Galleria mellonella* larvae following infection with representative *Staphylococcus haemolyticus* isolates. Larval survival was monitored for 120 h after inoculation. Panels represent: (**A**) hHo2, (**B**) hHo19, (**C**) sHo5, (**D**) hCa1, (**E**) hCa5, (**F**) sFe4, (**G**) hFe1, and (**H**) hFe13. For each isolate, the purple, blue, and green curves correspond to bacterial inoculum with OD_600_ values of 0.01, 0.1, and 0.5, respectively, whereas the yellow curve represents the corresponding run-specific PBS control. Isolates tested within the same experimental run shared the same PBS control group: hHo2/hHo19, hCa1/hCa5, hFe1/hFe13, and sHo5/sFe4. In the hCa1 and hCa5 panels, the OD_600_ = 0.01 curve overlaps the PBS control because no mortality occurred in either group.

The pathogenic potential of eight representative *Staphylococcus haemolyticus* isolates was evaluated using the *Galleria mellonella* infection model at three inoculum levels (OD_600_ = 0.01, 0.1, and 0.5). Kaplan–Meier survival analysis demonstrated significant differences among the inoculum groups and the corresponding PBS control for each of the eight isolates (log-rank χ^2^ = 27.91–61.80, df = 3, all *p* < 0.001; Table 4). In general, larval mortality increased with increasing inoculum, although the magnitude of this effect varied between isolates (Figure 5).

At the highest inoculum (OD_600_ = 0.5), mortality at 120 h ranged from 63.3% to 90.0%. The highest mortality was observed for hFe1 (90.0%), followed by hHo2 (86.7%). Mortality reached 80.0% for hCa5 and hFe13, 76.7% for hHo19, 70.0% for sHo5 and sFe4, and 63.3% for hCa1. Median larval survival times (LT_50_) at OD_600_ = 0.5 ranged from 24 to 108 h. The shortest LT_50_ values were observed for hHo2 and hFe13 (24 h), followed by hHo19, hCa5, and hFe1 (48 h), sHo5 and sFe4 (96 h), and hCa1 (108 h).

At OD_600_ = 0.1, mortality at 120 h ranged from 10.0% to 76.7%. LT_50_ was reached only for hFe13 (48 h) and hCa5 (96 h), whereas median survival was not reached within the 120-h observation period for the remaining isolates. At OD_600_ = 0.01, mortality remained lower, ranging from 0% to 26.7%, and LT_50_ was not reached for any isolate. Four independent PBS control groups were included across the experimental runs. No mortality was observed in three PBS control groups, whereas one larval death occurred in the PBS control group corresponding to the sHo5/sFe4 experimental run.

CFU enumeration was available for two representative isolates and confirmed that increasing OD_600_ values were associated with increasing viable bacterial concentrations. However, because CFU measurements were not available for all eight isolates, direct quantitative comparisons of bacterial load between strains should be interpreted cautiously. Overall, the assay demonstrated clear inoculum-dependent larval mortality together with substantial strain-level variation in survival phenotype. Because the experimental design and statistical analyses compared inoculum levels within individual isolates rather than host species or clinical-status groups, the observed differences between isolates were interpreted descriptively and should not be considered evidence of host- or clinical-status-associated pathogenicity.

## 3. Discussion

Accurate species identification is essential in epidemiological studies of coagulase-negative staphylococci (CoNS), particularly for species such as *Staphylococcus haemolyticus*, which may display similar phenotypic characteristics despite substantial differences in antimicrobial resistance, genomic background, and pathogenic potential. In the present study, species identity was established using MALDI-TOF MS and confirmed by detection of the species-specific *mvaA* gene, providing a robust basis for subsequent phenotypic and genomic comparisons. The high accuracy of MALDI-TOF MS for the identification of *S. haemolyticus*, particularly when combined with molecular confirmation, has been demonstrated previously [13,14].

Proteomic profiling, partial *rpoB* sequencing, and core-genome phylogenetic analysis consistently indicated substantial diversity within the analysed *S. haemolyticus* population. MALDI-TOF MS revealed partial host-associated structuring, including several feline-dominated clusters, but feline isolates were also present in mixed clusters together with human and canine isolates. Likewise, neither partial *rpoB* phylogeny nor core-genome analysis revealed clearly separated host- or clinical-status-specific lineages. These observations support the view that *S. haemolyticus* populations may show considerable within-species diversity and only limited host-associated structuring [16,17,18,19]. Importantly, the absence of clearly separated host-specific lineages should not be interpreted as evidence of direct interspecies transmission, as no epidemiologically linked human–animal isolate pairs were available in the present study.

Whole-genome analysis also demonstrated substantial overlap among isolates from different host species, although selected host-associated differences were identified. In the primary analysis of the 35 genomes, *mecA* and *blaZ*/PC1-family determinants were significantly associated with host species after false discovery rate correction. However, in the sensitivity analysis restricted to one isolate per individual host, only the association involving *mecA* remained statistically significant after FDR correction, whereas the *blaZ*/PC1-family association no longer reached the significance threshold. This indicates that the *mecA* signal was comparatively robust to the non-independence of isolates originating from the same host, whereas the *blaZ*/PC1-family result was sensitive to the analytical treatment of repeated host sampling. No acquired antimicrobial resistance determinant was significantly associated with clinical status after FDR correction in either analysis. These findings indicate that broad genomic overlap can coexist with differences in the frequency of individual resistance determinants, but also demonstrate that some host-associated signals may be sensitive to dataset structure. Therefore, because the final WGS dataset was relatively small and unbalanced across host and clinical-status groups, these associations should be regarded as exploratory and should not be interpreted as evidence of stable host-specific resistance profiles.

Phenotypic antimicrobial resistance was common in the analysed collection. Resistance was most frequently observed to erythromycin, ampicillin, penicillin, clindamycin, and tetracycline, and 45.13% of isolates fulfilled the definition of multidrug resistance. These findings are consistent with previous studies describing *S. haemolyticus* as one of the most antimicrobial-resistant CoNS species and as an important reservoir of resistance determinants in both human and veterinary settings [1,2,20,21,22]. Resistance phenotypes were highly heterogeneous, with 40 distinct susceptibility profiles identified among 113 isolates. Although descriptive differences were observed among host groups, the unequal group sizes, particularly the relatively small canine group and the absence of clinically affected canine isolates, limit the strength of conclusions regarding host-specific resistance patterns.

At the molecular level, *blaZ* was the most frequently detected resistance determinant in the full collection, followed by *tetL*, whereas *mecA*, *aac(6′)-Ie-aph(2″)-Ia*, *tetK*, and *fusB* occurred less frequently. Other genes, including *ermC* and *mupA*, were uncommon. Whole-genome sequencing broadened this picture by identifying additional acquired resistance determinants as well as widely distributed intrinsic resistance-associated genes, multidrug efflux systems, and regulatory components. The predominance of β-lactam resistance determinants, together with frequent macrolide-, lincosamide-, and tetracycline-associated genes, is consistent with the broad resistance potential previously described for *S. haemolyticus* [16,20,23]. At the same time, the host-associated differences observed for *mecA* and *blaZ*/PC1 in the WGS subset suggest that the frequency of individual resistance mechanisms may vary among host populations even when the overall genomic background does not form clearly separated host-specific groups.

Genotype–phenotype concordance varied considerably depending on both the antimicrobial agent and the phenotypic susceptibility method. Agreement was relatively high for some gene–phenotype combinations and substantially lower for others, demonstrating that the presence of a resistance determinant was not uniformly predictive of phenotypic susceptibility. In particular, concordance for *mecA* depended strongly on whether oxacillin susceptibility was assessed by disk diffusion or MIC, while agreement for other determinants also varied according to the antimicrobial examined. Importantly, the higher concordance observed between *mecA* and the operational oxacillin disk-diffusion classification should not be interpreted as evidence that disk diffusion is preferable to MIC for susceptibility assessment in *S. haemolyticus*. Rather, this difference illustrates the method-dependent nature of genotype–phenotype concordance, while MIC-based classification was considered the primary oxacillin susceptibility phenotype. Conversely, phenotypic resistance was observed in some isolates in the absence of the corresponding determinants included in the targeted PCR panel. Such discordance may reflect additional resistance mechanisms, differences in gene expression, methodological factors, or limitations of the applied interpretive criteria. These findings support the complementary use of phenotypic susceptibility testing and molecular resistance profiling rather than reliance on either approach alone [24,25,26,27]. They also indicate that antimicrobial treatment decisions should not be based solely on resistance-gene detection and that isolate-specific susceptibility testing remains important.

Temperature had a pronounced effect on biofilm formation but a much less uniform effect on planktonic growth. Growth kinetics varied among the representative isolates, whereas quantitative biofilm production increased markedly at 39 °C across the collection. No significant effects of host origin or clinical status on biofilm formation were detected, and no significant interactions between these factors and temperature were observed. These findings were retained in the one-isolate-per-host sensitivity analysis, indicating that the pronounced temperature effect and the absence of host- or clinical-status-associated effects were not driven by repeated sampling of individual hosts. Although the 37 °C versus 38 °C comparison reached statistical significance in the sensitivity-analysis subset, the principal finding of markedly enhanced biofilm production at 39 °C remained unchanged. Thus, enhanced biofilm formation at 39 °C appeared to be broadly distributed among the analysed isolates rather than restricted to a particular host or clinical-status group. Similar temperature- and stress-associated changes in adhesion and biofilm formation have been reported for other *Staphylococcus* species [28,29,30,31].

The divergence between planktonic growth and biofilm production is particularly noteworthy. The temperature associated with maximal planktonic proliferation did not consistently correspond to that promoting the strongest biofilm formation, indicating that the observed biofilm response cannot be explained solely by changes in growth kinetics. Accordingly, the increased biofilm formation at 39 °C should be interpreted as a temperature-associated phenotype rather than as a specific fever-related response. In the context of the present study, 39 °C may represent a physiologically relevant host temperature for dogs and cats and a febrile temperature in humans, but it may also impose direct thermal or incubation-related physiological stress on bacterial cells. These different effects cannot be distinguished experimentally on the basis of the present data. Although *clpC* and *clpP* were detected in all sequenced genomes as VFDB-associated loci, their presence alone cannot establish a functional role in the temperature-dependent biofilm phenotype. Gene expression was not assessed, and any mechanistic link between these loci and the response to elevated temperature therefore remains speculative. Transcriptomic or targeted functional studies would be required to investigate these mechanisms further.

The *Galleria mellonella* infection model provided an additional, complementary assessment of pathogenic potential. All eight tested isolates produced inoculum-dependent reductions in larval survival, while the magnitude of mortality varied among strains. These findings confirm that *S. haemolyticus* can produce measurable pathogenic effects in this model and demonstrate substantial between-isolate variability. However, the experiment was designed to compare inoculum-dependent responses within individual isolates rather than to formally test differences according to host species or clinical status. Consequently, the absence of an obvious host- or clinical-status-associated pattern among the eight tested isolates should not be interpreted as evidence of equivalent virulence across host groups. The limited number of isolates and the use of OD_600_-based inoculum standardization, with CFU enumeration available for only two strains, further constrain quantitative between-strain comparisons. Nevertheless, the dose-dependent response observed across the tested isolates supports the usefulness of *G. mellonella* as a comparative screening model for *S. haemolyticus* pathogenicity [32,33,34].

Taken together, the results demonstrate extensive phenotypic and genomic overlap among human, feline, and canine *S. haemolyticus* isolates, accompanied by substantial between-isolate heterogeneity and selected host-associated differences in individual resistance determinants. The present study does not provide evidence of direct transmission between humans and companion animals. Rather, it shows that related resistance, biofilm, genomic, and pathogenicity-associated characteristics may occur among isolates from different host populations. This pattern supports the relevance of *S. haemolyticus* within a One Health surveillance framework, particularly because antimicrobial resistance phenotypes and resistance determinants were identified among isolates from both human and animal hosts [16,19,20].

This study has several limitations that should be considered when interpreting the findings. The isolate collection was archival and originated from a single centre, with unequal representation of host and clinical-status groups, including a relatively small canine group, a limited number of isolates from clinically affected humans, and no isolates from clinically affected dogs. Because multiple isolates originated from some individual hosts, host-level non-independence was a potential source of bias in analyses involving host species and clinical status; this was addressed by additional sensitivity analyses restricted to one isolate per host for both biofilm and WGS host-associated comparisons. Forty isolates were selected for whole-genome sequencing, of which 35 met the predefined genome-quality criteria and were retained for comparative genomic analyses. The resulting WGS dataset was unbalanced across host and clinical-status groups; consequently, host-associated genomic comparisons should be regarded as exploratory. The *Galleria mellonella* experiments included eight representative isolates, and bacterial inocula were standardized primarily by OD_600_, with CFU enumeration available for only two isolates. Therefore, direct quantitative comparisons of viable bacterial loads among strains were limited. In addition, no epidemiologically linked human–animal isolate pairs were available, precluding conclusions regarding direct interspecies transmission. For antimicrobial susceptibility testing, validated *S. haemolyticus*-specific breakpoints were not available for all agents, and some interpretive criteria were therefore extrapolated from other staphylococci or historical standards. In particular, oxacillin disk-diffusion results were treated only as an operational comparative phenotype because this method is not a CLSI-supported primary susceptibility approach for *S. haemolyticus*; MIC-based classification was therefore considered the primary oxacillin susceptibility phenotype. Finally, although several VFDB-associated loci were detected, their expression and functional activity were not investigated. These limitations should be considered when extrapolating the observed host-associated, genomic, resistance, biofilm, and pathogenicity patterns beyond the analysed collection.

## 4. Materials and Methods

### 4.1. S. haemolyticus Strains Collection

The strain collection used in this study consisted of 113 *Staphylococcus haemolyticus* isolates obtained from 84 individual hosts, including 21 humans, 57 cats, and 6 dogs. The isolates originated from samples collected in Wrocław, Poland, between 2013 and 2022 and were obtained from the archive of the Department of Epizootiology and the Clinic of Birds and Exotic Animals, Faculty of Veterinary Medicine, Wrocław University of Environmental and Life Sciences.

The collection included isolates recovered from both healthy and clinically affected individuals. For the purposes of this study, healthy individuals were defined as those showing no apparent clinical signs of disease at the time of sampling, whereas clinically affected individuals were defined as those presenting with clinical signs or conditions at the time of sampling, including cough, fever, conjunctivitis, wound infection, skin lesions, or other clinically apparent abnormalities. Inclusion in the present study required confirmed identification of the isolate as *S. haemolyticus* and the availability of basic metadata concerning host species, clinical status, and sampling site.

More than one isolate could originate from the same individual when samples were obtained from different anatomical sites. Consequently, the 113 isolates represented 84 individual hosts. Specifically, 25 human isolates were obtained from 21 individuals, 80 feline isolates from 57 individuals, and 8 canine isolates from 6 individuals. The detailed distribution of isolates according to host species, clinical status, number of isolates, number of individuals, and sampling site is presented in Table 5.

No known epidemiologically linked human–animal isolate pairs were present in the collection. Two within-species household links were identified: one involving two human individuals and one involving two dogs originating from the same household. No human and animal isolates were known to be linked by household, clinic, sampling location, or common sampling event. Therefore, the human–animal comparisons performed in this study should be interpreted as population-level comparisons rather than analyses of directly epidemiologically linked host pairs.

Human sampling was conducted within a research project approved by the Bioethics Committee of the Medical University of Wrocław, Poland (approval No. KB-814/2019), and written informed consent was obtained from all human participants. Animal sampling involved non-invasive procedures and did not require separate ethical approval under the applicable regulations. The present study was conducted exclusively using archived bacterial isolates.

Following prior cultivation, all bacterial strains were stored at −80 °C in Brain Heart Infusion (BHI) broth (OXOID Ltd., Basingstoke, UK) supplemented with 40% (*v*/*v*) glycerol (Thermo Fisher Scientific, Kandel, Germany) in Eppendorf tubes.

### 4.2. Species Identification and Phylogenetic Analysis

A total of 113 archived bacterial isolates were included in the study. All isolates were cultured on Mannitol Salt Agar (OXOID Ltd., Basingstoke, UK) and Columbia Agar supplemented with 5% sheep blood (OXOID Ltd., Basingstoke, UK), followed by aerobic incubation at 37 °C for 24 h. Preliminary identification as members of the genus *Staphylococcus* was based on colony morphology and the tube coagulase test (Immunolab, Gdańsk, Poland). Species identification was performed using matrix-assisted laser desorption/ionization time-of-flight mass spectrometry (MALDI-TOF MS) with a Bruker ultrafleXtreme MALDI-TOF mass spectrometer (Bruker Daltonics, Bremen, Germany). To ensure reproducibility, each isolate was analyzed in duplicate, with two technical replicates prepared from each protein extract. Protein extraction was carried out using the standard ethanol-formic acid protocol. Briefly, single colonies were suspended in sterile water, mixed with absolute ethanol (Sigma-Aldrich, St. Louis, MO, USA), and centrifuged. The pellet was subsequently treated with 70% formic acid (Sigma-Aldrich, St. Louis, MO, USA) and acetonitrile (Honeywell, Charlotte, NC, USA), followed by a second centrifugation. One microliter of the resulting supernatant was spotted onto a polished steel MALDI target plate, air-dried, and overlaid with 1 µL of a saturated α-cyano-4-hydroxycinnamic acid (HCCA) matrix solution (10 mg/mL in acetonitrile-water-trifluoroacetic acid, 50:47.5:2.5, *v*/*v*/*v*) (Honeywell, Charlotte, NC, USA). Mass spectra were acquired and processed according to the manufacturer’s recommendations. To evaluate proteomic relatedness among the isolates, hierarchical cluster analysis and principal component analysis (PCA) were performed based on MALDI-TOF MS spectral peak profiles. Species identity was further confirmed by PCR amplification of the species-specific *mvaA* gene [13]. In addition, selected representative isolates were subjected to partial sequencing of the *rpoB* gene using previously published primers and amplification conditions [35]. 

Consensus sequences were assembled and compared with reference sequences deposited in the NCBI GenBank database using the NCBI BLASTn web service (https://blast.ncbi.nlm.nih.gov/Blast.cgi, accessed on 1 October 2026). Phylogenetic analysis was performed in MEGA11, version 11.0.11, using the Neighbor-Joining method, with evolutionary distances calculated according to the Tamura–Nei model. The partial rpoB gene sequences generated in this study were deposited in the NCBI GenBank database under accession numbers PZ747271–PZ747283, PZ747285–PZ747301, and PZ747303–PZ747307. The corresponding isolate identifiers and accession numbers are provided in Supplementary Table S1. The robustness of the inferred phylogeny was evaluated by bootstrap analysis with 1000 replicates.

### 4.3. Whole-Genome Sequencing and Comparative Genomic Analysis

A total of 40 isolates were selected for whole-genome sequencing from the available strain collection to provide representation of the investigated host species and clinical-status groups. Although phenotypic antimicrobial susceptibility results and resistance-gene profiles were available at the time of selection, these characteristics were not used as formal inclusion criteria. Isolates were selected primarily to ensure representation of the investigated host species and clinical-status groups. Sequence type and other genomic characteristics were not known before sequencing and therefore could not influence isolate selection. The number of isolates available within individual host and clinical-status categories was limited, resulting in an unbalanced WGS subset.

Bacterial genomes were sequenced on the Oxford Nanopore Technologies platform using the SQK-RBK114-24 rapid barcoding kit. Raw signal data were base called and demultiplexed with Dorado v1.0.2 using the SUP model (dna_r10.4.1_e8.2_400bps_sup@v5.2.0). Reads were filtered with Chopper, retaining reads at least 500 bp long and with a mean Q-score of at least 10 [36]. Filtered reads were assembled de novo with Flye v2.9 in nano-hq mode [37]. Assembly completeness and contamination were estimated using CheckM2 v2.0.1 [38]. Genome assemblies were considered suitable for downstream comparative genomic analyses when their total length was between 2.2 and 2.8 Mb, estimated completeness was ≥90%, contamination was ≤5%, and the number of contigs was <200. Following quality assessment, five assemblies were excluded because their estimated completeness was below the 90% threshold, resulting in a final WGS dataset of 35 isolates. The final dataset comprised 11 human, 7 canine, and 17 feline isolates; 22 originated from clinically healthy hosts and 13 from clinically affected hosts. No isolates from clinically affected dogs were available. The 35 isolates included in the final WGS dataset represented 33 unique individual hosts. Two hosts were represented by two isolates each. To assess the potential effect of non-independence resulting from multiple isolates obtained from the same host, an additional sensitivity analysis was performed using one isolate per individual host. When more than one isolate originated from the same host, the isolate with the lowest numerical isolate identifier was retained, resulting in a sensitivity-analysis subset of 33 isolates. Detailed isolate-level metadata, including host species, clinical status, sampling site, and genome assembly quality metrics, are provided in Supplementary Table S2. Assemblies retained for downstream analyses were annotated using Bakta v1.12.0 with database version 6.0 (light) [39]. 

#### 4.3.1. Multilocus Sequence Typing

Multilocus sequence typing was performed in silico for all 35 *Staphylococcus haemolyticus* genome assemblies included in the final WGS dataset using the seven-locus MLST scheme comprising *arcC*, *SH_1200*, *hemH*, *leuB*, *SH1431*, *cfxE*, and *Ribose_ABC*. Allelic profiles were compared with the *S. haemolyticus* profiles available in the PubMLST database [40].

Complete and unambiguous seven-locus allelic profiles matching an existing database profile were assigned to the corresponding sequence type (ST). Profiles containing ambiguous allele calls were retained as unresolved and were not assigned to an ST, whereas profiles lacking one or more loci were classified as incomplete. Complete seven-locus profiles without an initial ST assignment were manually rechecked against the current PubMLST database before final classification.

#### 4.3.2. Pangenome Reconstruction and Core-Genome Phylogenetic Analysis

Pangenome reconstruction was performed using the 35 high-quality *Staphylococcus haemolyticus* genome assemblies retained for comparative genomic analysis. Annotated assemblies were analysed using Panaroo v1.6.1 in strict clean mode [41]. Gene clusters present in at least 95% of the analysed genomes were defined as belonging to the core genome.

Individual core-gene clusters were aligned using MAFFT v7.5 [42], and the resulting alignments were concatenated to generate the core-genome alignment used for phylogenetic reconstruction. Maximum-likelihood phylogenetic analysis was performed using IQ-TREE v2.4.0 [43]. The optimal nucleotide substitution model was selected automatically using ModelFinder Plus, resulting in the GTR+F+I+R3 model [44]. Branch support was evaluated using 1000 SH-aLRT replicates [45].

Pairwise maximum-likelihood genetic distances were obtained from the core-genome alignment using IQ-TREE and are expressed as substitutions per site. Pairwise distance data were available for 35 isolates included in the final WGS dataset. The resulting core-genome phylogeny comprised the 35 study isolates retained after genome quality assessment.

#### 4.3.3. Antimicrobial Resistance Determinant Analysis

Antimicrobial resistance determinants were screened against the Comprehensive Antibiotic Resistance Database (CARD) [46] using ABRicate v1.4.0 [47]. The CARD used for the analysis was dated 3 April 2026. For the analyses reported in the present study, antimicrobial resistance determinants were evaluated in the 35 genome assemblies included in the final WGS dataset. Hits were retained using minimum thresholds of 70% nucleotide identity and 60% sequence coverage.

Acquired antimicrobial resistance determinants were analysed separately from broadly distributed intrinsic resistance-associated genes, multidrug efflux systems, and regulatory components. Genes associated with reduced susceptibility to antiseptic and disinfectant compounds were also recorded and considered separately from acquired antibiotic resistance determinants.

The distribution of acquired antimicrobial resistance determinants according to host species and clinical status was evaluated within the final WGS subset. Associations with host species were assessed using the Fisher–Freeman–Halton exact test for 3 × 2 contingency tables, whereas associations with clinical status were evaluated using two-sided Fisher’s exact tests. Benjamini–Hochberg false discovery rate correction was applied separately to the host-origin and clinical-status comparisons. An FDR-adjusted *q* < 0.05 was considered statistically significant. To assess the potential influence of non-independence resulting from multiple isolates obtained from the same individual host, the host- and clinical-status-associated analyses were additionally repeated in a sensitivity-analysis subset restricted to one isolate per host (n = 33). The same Fisher–Freeman–Halton exact tests, two-sided Fisher’s exact tests, and Benjamini–Hochberg false discovery rate correction procedures were applied to this reduced dataset.

#### 4.3.4. WGS-Based Genotype–Phenotype and PCR–WGS Concordance Analyses

Genotype–phenotype concordance was assessed among the 35 isolates included in the final WGS dataset by comparing WGS-detected acquired antimicrobial resistance determinants with phenotypic susceptibility classifications obtained by disk diffusion and MIC testing. Disk-diffusion and MIC-based classifications were analysed independently where both were available. For antimicrobial classes associated with multiple resistance determinants, an isolate was considered genotype-positive when at least one of the corresponding determinants was detected. Agreement was quantified using Cohen’s κ, and directional discordance between genotype and phenotype was evaluated using McNemar’s exact test.

Concordance between PCR- and WGS-based detection of antimicrobial resistance genes was assessed gene by gene for the same 35 isolates. Overall agreement and Cohen’s κ were calculated, and asymmetry between PCR-positive/WGS-negative and PCR-negative/WGS-positive discordant results was assessed using McNemar’s exact test. Cohen’s κ was not estimated for genes uniformly negative by both methods because of the absence of variation.

#### 4.3.5. Analysis of VFDB-Associated Loci

Genome assemblies included in the final WGS dataset were screened against the Virulence Factor Database (VFDB) [48] using ABRicate v1.4.0 [47]. Hits were retained using minimum thresholds of 70% nucleotide identity and 60% sequence coverage. The resulting VFDB-associated hits were converted into a binary presence/absence matrix for the 35 study genomes. Loci detected in at least one genome were summarized according to their VFDB functional category and virulence-factor family.

The distribution of variable VFDB-associated loci was compared according to host origin and clinical status. Loci that were uniformly present or uniformly absent across all 35 genomes were excluded from inferential statistical testing because they contained no between-isolate variation. Associations with host species were assessed using the Fisher–Freeman–Halton exact test for 3 × 2 contingency tables, whereas associations with clinical status were evaluated using two-sided Fisher’s exact tests. Benjamini–Hochberg false discovery rate correction was applied separately to the host-origin and clinical-status analyses. FDR-adjusted *q* < 0.05 was considered statistically significant. To evaluate the potential effect of non-independence resulting from multiple isolates originating from the same individual host, the host- and clinical-status-associated analyses of variable VFDB-associated loci were additionally repeated in a sensitivity-analysis subset restricted to one isolate per host (n = 33). The same Fisher–Freeman–Halton exact tests, two-sided Fisher’s exact tests, and Benjamini–Hochberg false discovery rate correction procedures were applied to this reduced dataset.

### 4.4. Antimicrobial Susceptibility Testing

Phenotypic antimicrobial susceptibility was assessed by the disk diffusion method on Mueller–Hinton agar using standardized antimicrobial susceptibility testing procedures. The antimicrobial panel comprised amoxicillin/clavulanic acid (AMC, 20/10 µg), ampicillin (AMP, 10 µg), ciprofloxacin (CIP, 5 µg), chloramphenicol (C, 30 µg), clindamycin (DA, 2 µg), erythromycin (E, 15 µg), fusidic acid (FD, 10 µg), gentamicin (CN, 10 µg), linezolid (LZD, 30 µg), oxacillin (OX, 1 µg), mupirocin (MUP, 200 µg), penicillin G (P, 10 U), rifampin (RD, 5 µg), tetracycline (TET, 30 µg), tigecycline (TGC, 15 µg), tobramycin (TOB, 10 µg), and trimethoprim/sulfamethoxazole (SXT, 1.25/23.75 µg) (Liofilchem, Roseto degli Abruzzi, Italy), as well as marbofloxacin (MAR, 5 µg) (MASTDISCS^®^, Mast Group Ltd., Liverpool, UK). *Staphylococcus aureus* ATCC 25923 was used as the quality control strain.

Inhibition zone diameters were interpreted using antimicrobial-specific criteria selected from the most appropriate available standards. CLSI M100 criteria were applied where active interpretive breakpoints for staphylococci were available [49]. Veterinary-specific criteria from CLSI VET01S were applied where appropriate [50]. Where current criteria were unavailable, previously established CLSI interpretive criteria were retained, including historical breakpoints for selected antimicrobial agents [51,52,53]. Fusidic acid and mupirocin were interpreted according to EUCAST criteria [54], with the *S. aureus* criterion used as a surrogate for mupirocin. Tigecycline was interpreted using a published disk-diffusion criterion for *Staphylococcus* spp. [55].

Because validated species-specific breakpoints are not available for all antimicrobial agents in *S. haemolyticus*, some classifications were based on criteria established for *Staphylococcus* spp. or, where indicated, another staphylococcal species. These classifications were therefore treated as operational interpretive categories and were considered cautiously when comparing isolates of different host origin. The disk contents, interpretive thresholds, breakpoint sources, and species applicability for each antimicrobial agent are provided in Supplementary Table S16.

For oxacillin, MIC-based classification was considered the primary susceptibility phenotype because oxacillin disk diffusion is not a CLSI-supported primary susceptibility method for *S. haemolyticus*. Disk-diffusion results were nevertheless retained as an operational phenotypic classification for comparative purposes and were analysed independently from MIC-based susceptibility. Accordingly, oxacillin disk-diffusion categories were used only for descriptive and comparative analyses, including genotype–phenotype concordance and descriptive resistance-profile analysis, and were not interpreted as definitive CLSI-validated susceptibility classifications for *S. haemolyticus*. For MDR classification, MIC-based oxacillin susceptibility was used in place of the operational disk-diffusion category. Oxacillin disk-diffusion classification was therefore not used to determine MDR status. Isolates that were *mecA*-positive but classified as oxacillin-susceptible by MIC were retained as phenotypically susceptible for the purposes of MDR classification. Because resistance genes were assessed solely on a presence/absence basis and gene expression was not evaluated, *mecA* carriage was treated as a genotypic resistance determinant and did not override the MIC-based phenotypic classification.

Multidrug resistance (MDR) was defined as resistance to at least one antimicrobial agent in three or more antimicrobial classes, according to Magiorakos et al. [15]. Antimicrobial agents were grouped into antimicrobial classes according to the same classification.

Minimum inhibitory concentrations (MICs) of oxacillin, erythromycin, clindamycin, tetracycline, vancomycin, and teicoplanin were determined by the broth microdilution method in Mueller–Hinton broth according to CLSI recommendations [49,50]. Two-fold serial dilutions of oxacillin, clindamycin, and tetracycline ranged from 0.03125 to 16 µg/mL; erythromycin from 0.0625 to 32 µg/mL; and vancomycin and teicoplanin from 0.5 to 128 µg/mL. *Staphylococcus aureus* ATCC 29213 was included as the quality control strain. The bacterial inoculum was prepared to a turbidity equivalent to 0.5 McFarland and subsequently diluted according to the broth microdilution procedure. The MIC was defined as the lowest antimicrobial concentration that completely inhibited visible bacterial growth.

The bactericidal endpoint was assessed by subculturing 10 µL aliquots from wells showing no visible growth onto Brain Heart Infusion agar (Oxoid, Basingstoke, UK), followed by incubation at 37 °C for 24 h. The bactericidal endpoint was defined as the lowest antimicrobial concentration at which no colonies were recovered after subculture. Because viable counts of the initial inoculum were not quantitatively determined for each assay, this endpoint was not interpreted as a directly verified ≥99.9% reduction in viable-cell count. For comparative purposes, the concentration corresponding to this endpoint was used in the MBC/MIC ratio calculation and expressed as log_2_(MBC/MIC), where a value of 0 indicated identical inhibitory and bactericidal endpoint concentrations, while increasing values reflected progressively larger differences between them.

### 4.5. Detection of mvaA and Antimicrobial Resistance Genes by PCR

Species identification was further confirmed by PCR amplification of the species-specific *mvaA* gene according to Schuenck et al. [13]. Genomic DNA from all isolates was extracted using the Genomic Mini AX Staphylococcus Spin Kit (A&A Biotechnology, Gdańsk, Poland) according to the manufacturer’s instructions. Genes associated with resistance to the major antimicrobial classes were detected using PCR-based approaches, including conventional PCR and probe-based real-time PCR (qPCR) assays. Probe-based qPCR assays were performed using fluorescently labelled hydrolysis probes with FAM or HEX reporter dyes and a BHQ-1 quencher.

Probe-based qPCR assays were applied for the detection of *blaZ* [56], *mecA* [57], *ermA*, *ermB*, and *ermC* [58], *tetK* [59], and *tetL*, *tetM*, and *tetO* [60]. Conventional PCR assays were used for the detection of *mecC* [61], *aac(6′)-Ie-aph(2″)-Ia* [62], *fusB* [63], *vanA* [64], *vanB* [65], and *mupA* [66]. Previously published primer and probe sequences were used as indicated in Supplementary Table S17, while the amplification conditions applied in the present study are reported therein. For *mecA*, the primer and probe sequences were adopted from Black et al. [57]; in the present study, these oligonucleotides were used for detection of *mecA* in genomic DNA rather than for assessment of gene expression. All antimicrobial resistance genes investigated in the present study were assessed solely on a presence/absence basis. Gene expression was not evaluated for any of the analysed resistance determinants. The specific primer and probe sequences, reaction mixtures, amplification conditions, expected amplicon sizes, and corresponding literature sources for each assay are provided in Supplementary Table S17.

Positive controls carrying the respective resistance determinants were included in all PCR assays to verify reaction performance. A no-template negative control consisting of nuclease-free water was included in each PCR/qPCR run to monitor for contamination. Conventional PCR results were considered positive when an amplicon of the expected size was detected by agarose gel electrophoresis. Probe-based qPCR results were considered positive when a specific amplification signal was obtained in the corresponding fluorescence channel, with no amplification observed in the negative control.

### 4.6. Bacterial Growth Kinetics and Biofilm Formation

Bacterial growth kinetics were evaluated using 17 representative *Staphylococcus haemolyticus* isolates (hHo2, hHo6, hHo19, sHo5, hCa1, hCa4, hCa5, hCa6, hCa8, hFe1, hFe13, hFe18, hFe32, hFe33, sFe1, sFe2, and sFe4), selected to represent different host species (humans, dogs, and cats), clinical-status groups (healthy and clinically affected), and diverse antimicrobial resistance profiles. Reference strains *S. haemolyticus* PCM 2113, *Staphylococcus epidermidis* PCM 1869, and *Staphylococcus aureus* ATCC 43300 were included for comparison. Overnight cultures were adjusted to an optical density at 600 nm (OD600) of approximately 0.1 in Brain Heart Infusion (BHI) broth (Oxoid, Basingstoke, UK). For each isolate and incubation temperature, three independently prepared bacterial inoculum were analysed as biological replicates and incubated at 37 °C, 38 °C, and 39 °C for 24 h under continuous shaking. Bacterial growth was monitored hourly by measuring OD_600_ using a Tecan Infinite 200 Pro M Nano microplate reader (Tecan, Männedorf, Switzerland).

Because the onset and duration of the exponential growth phase varied among strains and incubation temperatures, the exponential phase was identified individually for each growth curve as the contiguous interval showing an approximately linear increase in the natural logarithm of OD_600_ over time. The specific growth rate (μ, h^−1^) was estimated as the slope of a linear regression fitted to ln(OD_600_) values plotted against time within this interval. No predefined numerical R^2^ cutoff or other fixed threshold was applied for exponential-phase selection. The corresponding doubling time (tD, h) was calculated according to the equation tD = ln(2)/μ.

For descriptive purposes, temperature-dependent growth responses were grouped post hoc according to the direction and shape of changes in the specific growth rate across 37 °C, 38 °C, and 39 °C. The categories were not predefined before data analysis and were not derived from formal statistical clustering or threshold criteria. Five descriptive patterns were distinguished: (i) gradual decrease in growth rate with increasing temperature, (ii) gradual increase, (iii) maximum growth rate at 38 °C, (iv) decrease at 38 °C followed by recovery at 39 °C, and (v) temperature-independent growth characterized by only limited variation across the three temperatures.

Biofilm formation was assessed for all 113 *S. haemolyticus* isolates using the crystal violet microtiter plate assay (MTP) as previously described [67]. *Staphylococcus aureus* ATCC 29213 served as the positive control, whereas sterile Tryptone Soya Broth (TSB; Oxoid, Basingstoke, UK) was used as the negative control. Biofilm formation was evaluated after incubation at 37 °C, 38 °C, and 39 °C.

The biofilm assay was performed in two independent biological experiments conducted on separate days. In each experiment, two independently prepared bacterial cultures were analysed for each isolate, and each culture was tested in three technical replicate wells at each incubation temperature. Thus, six technical measurements were obtained per isolate and temperature in each biological experiment, giving a total of twelve technical measurements across the two independent experiments. Following crystal violet staining, biofilm biomass was quantified by measuring absorbance at 570 nm (A_570_).

Negative controls were included separately for each incubation temperature and experimental run. Because the optical-density values of the negative controls obtained at 37 °C, 38 °C, and 39 °C were highly comparable, the control measurements were pooled to establish a common cut-off optical density (ODc). ODc was calculated as the mean absorbance of the negative controls plus two standard deviations, according to the method described by Płoneczka-Janeczko et al. [67]. The resulting ODc was 0.1856. Isolates were classified as non-biofilm producers when A_570_ ≤ ODc, weak producers when ODc < A_570_ ≤ 2 × ODc, moderate producers when 2 × ODc < A_570_ ≤ 4 × ODc, and strong producers when A_570_ > 4 × ODc. Accordingly, the thresholds used were 0.1856, 0.3712, and 0.7424, respectively.

For quantitative analysis, the final A_570_ value for each isolate at each incubation temperature was calculated as the mean of the replicate measurements after subtraction of the corresponding background signal. Incubation temperatures of 37 °C, 38 °C, and 39 °C were selected to encompass physiologically relevant thermal conditions for the investigated host species. Approximately 37 °C corresponds to normal human body temperature, whereas temperatures approaching 38–39 °C are within or close to the physiological range of dogs and cats; in humans, 39 °C represents a febrile condition.

### 4.7. Pathogenic Potential Assessment Using the Galleria mellonella Infection Model

The pathogenic potential of *Staphylococcus haemolyticus* was evaluated using the *Galleria mellonella* infection model, based on the protocol described by Tsai et al. [33], with modifications adapted from our previous study [68]. Eight representative isolates (hHo2, hHo19, sHo5, hCa1, hCa5, hFe1, hFe13, and sFe4) were selected to represent different host species (humans, dogs, and cats), clinical-status categories, and diverse antimicrobial resistance profiles.

Overnight bacterial cultures were harvested by centrifugation at 3500 rpm, washed three times with sterile phosphate-buffered saline (PBS; Argenta, Poznań, Poland), and adjusted to OD_600_ values of 0.5, 0.1, and 0.01. For each isolate and inoculum level, 30 larvae weighing 300 ± 30 mg were used and divided into three parallel groups of 10 larvae. Each larva was inoculated with 10 µL of the corresponding bacterial suspension into the hemocoel using a Hamilton 100-µL microsyringe (Hamilton Bonaduz AG, Bonaduz, Switzerland).

The eight isolates were evaluated in four experimental runs, with two isolates tested in parallel within each run: hHo2 with hHo19, hCa1 with hCa5, hFe1 with hFe13, and sHo5 with sFe4. Each experimental run included one concurrent PBS-injected negative-control group consisting of 10 larvae. Thus, the two isolates tested within the same experimental run shared the same PBS control group.

Bacterial inocula were standardized primarily on the basis of OD_600_. CFU enumeration was additionally available for two representative isolates to provide an approximate relationship between optical density and viable bacterial concentration. For sHo5, viable-cell concentrations were approximately 6.17 × 10^6^ CFU/mL at OD_600_ = 0.5 and 1.97 × 10^6^ CFU/mL at OD_600_ = 0.1. For sFe4, the corresponding concentrations were approximately 5.33 × 10^6^ CFU/mL at OD_600_ = 0.5, 9.00 × 10^5^ CFU/mL at OD_600_ = 0.1, and 7.47 × 10^4^ CFU/mL at OD_600_ = 0.01. Because CFU measurements were not available for all eight isolates, identical OD_600_ values were not assumed to represent identical viable-cell concentrations across strains.

Following inoculation, larvae were incubated at 37 °C and monitored for 120 h, with survival recorded every 24 h. Larvae were considered dead when they exhibited complete melanization and failed to respond to tactile stimulation.

Survival data were analysed using the Kaplan–Meier method. For each isolate, survival distributions obtained for OD_600_ = 0.01, 0.1, and 0.5 were compared with the corresponding run-specific PBS control using the log-rank (Mantel–Haenszel) test implemented in JASP version 0.19.7.1 (JASP Team, University of Amsterdam, Amsterdam, The Netherlands). Median larval survival time (LT_50_) and its 95% confidence interval were determined from the Kaplan–Meier analysis when the median survival was reached during the 120-h observation period. Statistical comparisons were performed within individual isolates across inoculum levels and were not designed to test differences according to host species or clinical status.

### 4.8. Statistical Analysis

Statistical analyses were performed using Statistica 13 (TIBCO Software Inc., Tulsa, OK, USA) and JASP version 0.19.7.1 (JASP Team, University of Amsterdam, Amsterdam, The Netherlands). Quantitative biofilm production data (A_570_) obtained for each isolate under three incubation temperatures (37 °C, 38 °C, and 39 °C) were analysed using a linear mixed-effects model to account for repeated measurements of the same isolates. Incubation temperature, host origin, and clinical status were included as fixed effects, while isolate identity was included as a random effect. The model also included temperature × host origin and temperature × clinical status interaction terms. Pairwise comparisons between incubation temperatures were performed using estimated marginal means derived from the linear mixed-effects model, with Holm adjustment for multiple comparisons. A *p*-value < 0.05 was considered statistically significant.

To assess the potential impact of non-independence arising from multiple isolates obtained from the same individual host, an additional sensitivity analysis was performed using one isolate per host. When more than one isolate originated from the same individual, the isolate with the lowest numerical isolate identifier was retained, resulting in a subset of 84 isolates representing 84 unique hosts. The same linear mixed-effects model, including the same fixed effects, interaction terms, random effect, and Holm-adjusted pairwise comparisons, was applied to this one-isolate-per-host subset.

Agreement between phenotypic antimicrobial resistance and the presence of corresponding antimicrobial resistance genes was assessed using McNemar’s test and Cohen’s kappa coefficient. Survival data obtained from the *Galleria mellonella* infection model were analysed using the Kaplan–Meier method. Differences between survival curves were assessed using the log-rank (Mantel–Haenszel) test. Statistical analyses of whole-genome sequencing data and phylogenetic analyses were performed using the bioinformatic tools described in Section 4.3.

## Figures and Tables

**Table 1:** Resistance (%) to selected antibiotics in *S. haemolyticus* strains from humans and animals determined by the disk diffusion method.

Group	AMP	AMC	MUP	P	FD	MAR	CIP	OX *	LZD	DA	E	C	RD	CN	SXT	TGC	TET	TOB
Healthy humans (n = 19)	7/19 36.84%	1/19 5.26%	0/19 0.00%	7/19 36.84%	0/19 0.00%	1/19 5.26%	1/19 5.26%	1/19 5.26%	0/19 0.00%	7/19 36.84%	9/19 47.37%	0/19 0.00%	0/19 0.00%	1/19 5.26%	1/19 5.26%	0/19 0.00%	5/19 26.32%	1/19 5.26%
Clinically affected humans (n = 6)	0/6 0.00%	0/6 0.00%	0/6 0.00%	0/6 0.00%	0/6 0.00%	0/6 0.00%	0/6 0.00%	0/6 0.00%	0/6 0.00%	2/6 33.33%	5/6 83.33%	0/6 0.00%	0/6 0.00%	0/6 0.00%	0/6 0.00%	0/6 0.00%	3/6 50.00%	0/6 (0.00%)
Healthy dogs (n = 8)	4/8 50.00%	2/8 25.00%	0/8 0.00%	4/8 50.00%	1/8 12.50%	2/8 25.00%	2/8 25.00%	3/8 37.50%	0/8 0.00%	3/8 37.50%	3/8 37.50%	0/8 0.00%	0/8 0.00%	3/8 37.50%	1/8 12.50%	0/8 0.00%	3/8 37.50%	0/8 0.00%
Healthy cats (n = 36)	17/36 47.22%	7/36 19.44%	1/36 2.78%	18/36 50.00%	5/36 13.89%	4/36 11.11%	6/36 16.67%	8/36 22.22%	0/36 0.00%	14/36 38.89%	18/36 50.00%	2/36 5.56%	0/36 0.00%	4/36 11.11%	1/36 2.78%	0/36 0.00%	11/36 30.56%	1/36 2.78%
Clinically affected cats (n = 44)	37/44 84.09%	22/44 50.00%	2/44 4.55%	34/44 77.27%	0/44 0.00%	5/44 11.36%	5/44 11.36%	11/44 25.00%	0/44 0.00%	25/44 56.82%	36/44 81.82%	0/44 0.00%	0/44 0.00%	4/44 9.09%	1/44 2.27%	0/44 0.00%	28/44 63.64%	1/44 2.27%
All (n = 113)	65/113 57.52%	32/113 28.32%	3/113 2.65%	63/113 55.75%	6/113 5.31%	12/113 10.62%	14/113 12.39%	23/113 20.35%	0/113 0.00%	51/113 45.13%	71/113 62.83%	2/113 1.77%	0/113 0.00%	12/113 10.62%	4/113 3.54%	0/113 0.00%	50/113 44.25%	3/113 2.65%

AMP = ampicillin, AMC = amoxicillin/clavulanic acid, MUP = mupirocin, P = penicillin, FD = fusidic acid, MAR = marbofloxacin, CIP = ciprofloxacin, OX = oxacillin, LZD = linezolid, DA = clindamycin, E = erythromycin, C = chloramphenicol, RD = rifampicin, CN = gentamicin, SXT = trimethoprim/sulfamethoxazole, TGC = tigecycline, TET = tetracycline. * For oxacillin, disk-diffusion categories were treated as operational phenotypic classifications for descriptive and comparative purposes only. Oxacillin disk diffusion is not a CLSI-supported primary susceptibility method for *S. haemolyticus*; MIC-based classification was evaluated separately as the primary susceptibility phenotype.

**Table 2:** Occurrence of selected antibiotic resistance genes among *S. haemolyticus* strains from humans and animals (%).

Group	*blaZ*	*mecA*	*mecC*	*Aac* *	*ermA*	*ermB*	*ermC*	*tetK*	*tetL*	*tetO*	*tetM*	*fusB*	*vanA*	*vanB*	*mupA*
Healthy humans (n = 19)	4/19 21.05%	1/19 5.26%	0/19 0.00%	1/19 5.26%	0/19 0.00%	0/19 0.00%	1/19 5.26%	2/19 10.53%	2/19 10.53%	0/19 0.00%	0/19 0.00%	0/19 0.00%	0/19 0.00%	0/19 0.00%	0/19 0.00%
Clinically affected humans (n = 6)	0/6 0.00%	0/6 0.00%	0/6 0.00%	0/6 0.00%	0/6 0.00%	0/6 0.00%	2/6 33.33%	2/6 33.33%	0/6 0.00%	0/6 0.00%	1/6 16.67%	2/6 33.33%	0/6 0.00%	0/6 0.00%	0/6 0.00%
Healthy dogs (n = 8)	4/8 50.00%	2/8 25.00%	0/8 0.00%	3/8 37.50%	0/8 0.00%	0/8 0.00%	0/8 0.00%	2/8 25.00%	0/8 0.00%	0/8 0.00%	0/8 0.00%	4/8 50.00%	0/8 0.00%	0/8 0.00%	0/8 0.00%
Healthy cats (n = 36)	19/36 52.78%	4/36 11.11%	0/36 0.00%	3/36 8.33%	0/36 0.00%	0/36 0.00%	0/36 0.00%	2/36 5.56%	12/36 33.33%	0/36 0.00%	0/36 0.00%	3/36 8.33%	0/36 0.00%	0/36 0.00%	1/36 2.78%
Clinically affected cats (n = 44)	34/44 77.27%	12/44 27.27%	0/44 0.00%	8/44 18.18%	0/44 0.00%	0/44 0.00%	0/44 0.00%	5/44 11.36%	24/44 54.55%	0/44 0.00%	0/44 0.00%	3/44 6.82%	0/44 0.00%	0/44 0.00%	2/44 4.55%
All (n = 113)	61/113 53.98%	19/113 16.81%	0/113 0.00%	15/113 13.27%	0/113 0.00%	0/113 0.00%	3/113 2.65%	13/113 11.50%	38/113 33.63%	0/113 0.00%	1/113 0.88%	12/113 10.62%	0/113 0.00%	0/113 0.00%	3/113 2.65%

* *aac(6′)-Ie-aph(2″)-Ia2.4*. Detection of Resistance Genes Using PCR-Based Genotypic Analysis.

**Table 3:** Temperature-dependent growth patterns of representative *Staphylococcus haemolyticus* isolates and reference strains, with corresponding biofilm phenotypes for the study isolates.

Growth Pattern	No. of Strains	Representative Isolates	Predominant Biofilm Phenotype at 39 °C *
Gradual decrease	7	hHo2, hHo6, hHo19, hCa1, hCa4, hFe18, ATCC *43300*	Strong (6/6)
Gradual increase	2	hFe1, hFe32	Strong (1/2), Moderate (1/2)
Peak at 38 °C	6	hCa5, hFe13, sFe1, sFe2, *PCM 2113*, PCM *1869*	Strong (3/4), Moderate (1/4)
Decrease followed by recovery	4	sHo5, hCa6, hCa8, hFe33	Strong (4/4)
Temperature-independent	1	sFe4	Strong (1/1)

* Biofilm phenotype at 39 °C refers only to the 17 analysed *S. haemolyticus* study isolates. Reference strains (*S. aureus* ATCC 43300, *S. haemolyticus* PCM 2113, and *S. epidermidis* PCM 1869) were included exclusively in the growth-kinetics analysis and were therefore excluded from the biofilm-phenotype denominators. Biofilm formation was classified as weak, moderate, or strong according to the crystal violet assay.

**Table 4:** Kaplan–Meier survival analysis of *Galleria mellonella* larvae infected with selected *Staphylococcus haemolyticus* isolates.

Isolate	Host	Clinical Status	Mortality at 120 h, OD_600_ = 0.01 (%)	Mortality at 120 h, OD_600_ = 0.1 (%)	Mortality at 120 h, OD_600_ = 0.5 (%)	LT_50_ at OD_600_ = 0.5 (95% CI), h	χ^2^	df	*p*-Value
hHo2	Human	Healthy	20.0	43.3	86.7	24 (24–72)	41.97	3	<0.001
hHo19	Human	Healthy	10.0	30.0	76.7	48 (48–96)	39.23	3	<0.001
sHo5	Human	Clinically affected	10.0	20.0	70.0	96 (72–NE)	27.91	3	<0.001
hCa1	Dog	Healthy	0.0	10.0	63.3	108 (96–NE)	47.51	3	<0.001
hCa5	Dog	Healthy	0.0	76.7	80.0	48 (48–72)	61.80	3	<0.001
hFe1	Cat	Healthy	26.7	46.7	90.0	48 (24–72)	43.08	3	<0.001
hFe13	Cat	Healthy	13.3	70.0	80.0	24 (24–48)	41.10	3	<0.001
sFe4	Cat	Clinically affected	10.0	20.0	70.0	96 (96–NE)	32.71	3	<0.001

LT_50_, median larval survival time; NE, upper confidence limit not estimable. Log-rank tests compared survival distributions among the three inoculum levels and the corresponding run-specific PBS control within each isolate.

**Table 5:** Distribution of the analysed isolates and individual hosts according to host species, clinical status, and anatomical sampling site.

Host Species	Clinical Status	Isolates, n	Individuals, n	Sampling Sites, n of Isolates
Human	Healthy *	19	16	Skin (11), nostrils (5), ear (2), oral cavity (1)
Human	Clinically affected *	6	5	Skin (3), oral cavity (2), ear (1)
**Human total**		**25**	**21**	**Skin (14), nostrils (5), ear (3), oral cavity (3)**
Dog	Healthy	8	6	Anus (2), ear (2), conjunctival sac (2), oral cavity (1), nostrils (1)
Dog	Clinically affected	0	0	-
**Dog total**		**8**	**6**	**Anus (2), ear (2), conjunctival sac (2), oral cavity (1), nostrils (1)**
Cat	Healthy	36	34	Nostrils (11), conjunctival sac (10), skin (7), anus (3), ear (3), oral cavity (2)
Cat	Clinically affected	44	23	Conjunctival sac (15), skin (13), nostrils (11), anus (4), ear (1)
**Cat total**		**80**	**57**	**Conjunctival sac (25), nostrils (22), skin (20), anus (7), ear (4), oral cavity (2)**
**Total**		**113**	**84**	**Skin (34), nostrils (28), conjunctival sac (27), ear (9), anus (9), oral cavity (6)**

* Clinically healthy = no apparent clinical signs at the time of sampling; clinically affected = presenting with clinical signs or conditions at the time of sampling. Sampling-site counts refer to isolates rather than unique individual hosts, as more than one isolate obtained from different anatomical sites could originate from the same individual.

## Data Availability

Data is contained within the article and Supplementary Materials.

## Supplementary Materials

The following supporting information can be downloaded at: https://www.mdpi.com/article/10.3390/ijms27198905/s1.
