# Supplementary material for: Characteristics of Staphylococcus haemolyticus Isolated from Humans and Animals
Source: Int J Mol Sci. 2026 Oct 7;27(19):8905. doi: 10.3390/ijms27198905 (PMC13664951; doi:10.3390/ijms27198905)
Supplement: Supplementary file 1 [file ijms-27-08905-s001.zip › ijms-4525030-supplementary.pdf]

Supplementary material – ijms

Characteristics of *Staphylococcus haemolyticus* isolated from humans and animals

Paulina Prorok [1*], Magdalena Siedlecka [1], Magdalena Karwańska [1], Milena Skrok [1], Marta Miszczak [1], Maciej Wernecki [2], Marta Książczyk [2], Krzysztof Rypuła [1], Karolina Bierowiec [1*]

[1] Department of Epizootiology and Clinic of Birds and Exotic Animals, Division of Infectious Diseases and Veterinary Sciences, 50-365 Wrocław, Poland; magdalena.karwanska@upwr.edu.pl (M.K.); magdalena.siedlecka@upwr.edu.pl (M.S.); milena.skrok@upwr.edu.pl (M.S.); marta.miszczak@upwr.edu.pl (M.M.); krzysztof.rypula@upwr.edu.pl (K.R.)

[2] Department of Microbiology, Faculty of Biological Sciences, University of Wroclaw, 51-148 Wrocław, Poland; maciej.wernecki@uwr.edu.pl (M.W.); marta.ksiazczyk@uwr.edu.pl (M.K.),

* Correspondence: paulina.prorok@upwr.edu.pl (P.P.); karolina.bierowiec@upwr.edu.pl (K.B.)

Table S1. Characteristics of *Staphylococcus haemolyticus* isolates included in the *rpoB*-based phylogenetic analysis and corresponding GenBank accession numbers.

No.	Isolate ID	Host species	Clinical status	*rpoB* GenBank accession number
1	sHo2	Human	Clinically affected	PZ747271
2	hHo1	Human	Healthy	PZ747272
3	hHo2	Human	Healthy	PZ747273
4	sHo4	Human	Clinically affected	PZ747274
5	hHo5	Human	Healthy	PZ747275
6	hHo6	Human	Healthy	PZ747276
7	hHo19	Human	Healthy	PZ747277
8	sHo5	Human	Clinically affected	PZ747278
9	hHo10	Human	Healthy	PZ747279
10	hHo17	Human	Healthy	PZ747280
11	hCa1	Dog	Healthy	PZ747281
12	hFe6	Cat	Healthy	PZ747282
13	hFe1	Cat	Healthy	PZ747283
14	hFe13	Cat	Healthy	PZ747285
15	sFe23	Cat	Clinically affected	PZ747286
16	sFe24	Cat	Clinically affected	PZ747287
17	sFe25	Cat	Clinically affected	PZ747288
18	sFe26	Cat	Clinically affected	PZ747289
19	hCa6	Dog	Healthy	PZ747290
20	hFe15	Cat	Healthy	PZ747291
21	hFe19	Cat	Healthy	PZ747292
22	sFe19	Cat	Clinically affected	PZ747293
23	sFe3	Cat	Clinically affected	PZ747294
24	sFe4	Cat	Clinically affected	PZ747295
25	hCa7	Dog	Healthy	PZ747296
26	hCa8	Dog	Healthy	PZ747297
27	hFe26	Cat	Healthy	PZ747298
28	hCa2	Dog	Healthy	PZ747299
29	hFe32	Cat	Healthy	PZ747300
30	sFe1	Cat	Clinically affected	PZ747301
31	hFe33	Cat	Healthy	PZ747303
32	sFe2	Cat	Clinically affected	PZ747304
33	hCa5	Dog	Healthy	PZ747305
34	hCa4	Dog	Healthy	PZ747306
35	sHo3	Human	Clinically affected	PZ747307

Table S2. Genome assembly quality metrics for the 35 *Staphylococcus haemolyticus* isolates included in the final WGS dataset.

No.	ID	Host	Clinical Status	Assembly length (Mb)	No. of contigs	N50 (bp)	GC content (%)	Completeness (%)	Contamination (%)
1	**sHo2**	Human	Clinically affected	2.606	4	2,552,009	32.82	99.99	0.21
2	**hHo1**	Human	Healthy	2.558	6	2,460,016	32.99	100.00	0.25
3	**hHo2**	Human	Healthy	2.468	1	2,468,362	32.85	99.97	0.08
4	**sHo3**	Human	Clinically affected	2.460	66	77,842	32.91	91.66	3.13
5	**sHo4**	Human	Clinically affected	2.437	3	2,405,304	32.87	99.99	0.01
6	**hHo5**	Human	Healthy	2.646	4	2,488,446	32.92	100.00	0.14
7	**hHo6**	Human	Healthy	2.508	4	2,457,646	32.91	99.99	0.11
8	**hHo19**	Human	Healthy	2.518	2	2,491,410	32.91	99.98	0.17
9	**sHo5**	Human	Clinically affected	2.592	5	2,496,920	32.78	99.99	0.15
10	**hHo10**	Human	Healthy	2.508	2	2,456,400	32.89	99.99	0.16
11	**hHo17**	Human	Healthy	2.582	4	2,538,368	32.78	99.99	0.14
12	**hCa1**	Dog	Healthy	2.530	2	2,492,907	32.81	99.98	0.39
13	**hFe6**	Cat	Healthy	2.529	2	2,508,197	32.85	99.99	0.08
14	**hFe1**	Cat	Healthy	2.537	2	2,514,829	32.85	99.99	0.11
15	**hFe13**	Cat	Healthy	2.489	4	2,442,638	32.81	99.99	1.16
16	**sFe23**	Cat	Clinically affected	2.633	4	2,555,500	32.83	99.99	0.49
17	**sFe24**	Cat	Clinically affected	2.545	1	2,544,777	32.85	99.99	0.19
18	**sFe25**	Cat	Clinically affected	2.704	6	2,557,755	32.76	99.99	0.18
19	**sFe26**	Cat	Clinically affected	2.541	1	2,541,295	32.86	99.99	0.22
20	**hCa6**	Dog	Healthy	2.642	3	2,604,001	32.87	100.00	0.58
21	**hFe15**	Cat	Healthy	2.640	3	2,602,501	32.87	100.00	0.58
22	**hFe19**	Cat	Healthy	2.549	1	2,549,024	32.85	99.98	0.32
23	**sFe19**	Cat	Clinically affected	2.661	1	2,660,629	32.73	100.00	0.30
24	**sFe3**	Cat	Clinically affected	2.446	5	2,244,744	32.78	99.98	0.57
25	**sFe4**	Cat	Clinically affected	2.594	3	2,559,939	32.81	99.99	0.21
26	**hCa7**	Dog	Healthy	2.598	14	903,236	32.78	99.96	0.77
27	**hCa8**	Dog	Healthy	2.414	5	2,356,055	32.75	99.98	0.13
28	**hFe26**	Cat	Healthy	2.430	3	2,405,915	32.77	99.99	0.15
29	**hCa2**	Dog	Healthy	2.413	4	2,356,055	32.75	99.97	0.13
30	**hFe32**	Cat	Healthy	2.548	1	2,547,676	32.85	99.98	0.32
31	**sFe1**	Cat	Clinically affected	2.584	5	2,533,310	32.82	99.99	0.64
32	**hFe33**	Cat	Healthy	2.618	4	2,556,841	32.90	99.99	0.47
33	**sFe2**	Cat	Clinically affected	2.686	5	2,557,754	32.73	99.99	0.19
34	**hCa5**	Dog	Healthy	2.617	6	2,512,679	32.82	100.00	0.34
35	**hCa4**	Dog	Healthy	2.769	40	1,545,517	32.69	100.00	1.72

Note: Assembly quality was assessed using genome length, number of contigs, N50, GC content, estimated completeness, and contamination. All 35 genome assemblies included in the final WGS dataset met the predefined quality criteria of a genome length of 2.2–2.8 Mb, estimated completeness ≥90%, contamination ≤5%, and <200 contigs.

Table S3. Multilocus sequence typing results for 25 *Staphylococcus haemolyticus* isolates, including sequence type assignment, host origin, clinical status, and sampling site.

No.	ID	Host	Clinical status	Sampling site	sT	MLST assignment
1	**sHo2**	Human	Clinically affected	Oral cavity	46	Known ST assigned
2	**hHo1**	Human	Healthy	Oral cavity	48	Known ST assigned
3	**hHo2**	Human	Healthy	Skin	52	Known ST assigned
4	**sHo4**	Human	Clinically affected	Skin	115	Known ST assigned
5	**hHo5**	Human	Healthy	Skin	3	Known ST assigned
6	**hHo6**	Human	Healthy	Skin	52	Known ST assigned
7	**hHo19**	Human	Healthy	Skin	52	Known ST assigned
8	**sHo5**	Human	Clinically affected	Skin	52	Known ST assigned
9	**hHo10**	Human	Healthy	Nostrils	-	**Complete allelic profile, no known ST assigned**
10	**hCa1**	Dog	Healthy	Oral cavity	50	Known ST assigned
11	**sFe23**	Cat	Clinically affected	Nostrils	69	Known ST assigned
12	**sFe24**	Cat	Clinically affected	Nostrils	8	Known ST assigned
13	**sFe25**	Cat	Clinically affected	Nostrils	69	Known ST assigned
14	**hFe6**	Cat	Healthy	Nostrils	49	Known ST assigned
15	**hFe1**	Cat	Healthy	Nostrils	52	Known ST assigned
16	**hCa6**	Dog	Healthy	Nostrils	48	Known ST assigned
17	**hFe15**	Cat	Healthy	Conjunctival sac	48	Known ST assigned
18	**sFe19**	Cat	Clinically affected	Conjunctival sac	8	Known ST assigned
19	**sFe4**	Cat	Clinically affected	Conjunctival sac	69	Known ST assigned
20	**hCa7**	Dog	Healthy	Conjunctival sac	42	Known ST assigned
21	**hFe33**	Cat	Healthy	Skin	52	Known ST assigned
22	**hFe32**	Cat	Healthy	Skin	1	Known ST assigned
23	**sFe1**	Cat	Clinically affected	Skin	65	Known ST assigned
24	**sFe2**	Cat	Clinically affected	Ear canal	69	Known ST assigned
25	**hCa5**	Dog	Healthy	Ear canal	48	Known ST assigned

Note: hHo10 showed a complete seven-locus allelic profile but could not be assigned to a known sequence type and was therefore retained as an unassigned profile.

Table S4. Closest interspecies pairwise maximum-likelihood genetic distances among *Staphylococcus haemolyticus* isolates based on the core-genome alignment.

Isolates	Hosts	ML genetic distance
**hFe15 – hCa6**	Cat–Dog	0.000001103
**hHo2 – hFe33**	Human–Cat	0.000638007
**sHo5 – hFe33**	Human–Cat	0.000890360
**hHo6 – hFe33**	Human–Cat	0.000892351
**hHo19 – hFe33**	Human–Cat	0.000908911
**hHo2 – hFe1**	Human–Cat	0.000968259
**sHo5 – hFe1**	Human–Cat	0.001319716
**hHo6 – hFe1**	Human–Cat	0.001321589
**hHo19 – hFe1**	Human–Cat	0.001322105
**sHo4 – hCa4**	Human–Dog	0.001499244

Table S5. Distribution of antimicrobial resistance-associated genes among 35 *S. haemolyticus* isolates analyzed by whole-genome sequencing (WGS). "+" indicates the presence of a gene.

Isolate	tetM	emeA	efrB	lreK	dfrE	ermC	PC1	mecA	ANT(4')-Ia	tetL	sepA	sdrM	norA	mgrA	dfrC	lmrS	arlR	mepA	arlS	norC
hHo1	–	–	–	–	–	+	–	–	+	+	+	+	+	+	+	+	+	+	+	+
hHo2	–	–	–	–	–	–	–	–	–	–	+	+	+	+	+	+	+	+	+	+
hHo5	–	–	–	–	–	–	–	–	–	–	+	+	+	+	+	+	+	+	+	+
hHo6	–	–	–	–	–	–	–	–	+	–	+	+	+	+	+	+	+	+	+	+
hHo10	–	–	–	–	–	–	–	–	–	–	+	+	+	+	+	+	+	+	+	+
hHo17	–	–	–	–	–	–	–	–	–	–	+	+	+	+	+	+	+	+	+	+
hHo19	–	–	–	–	–	–	–	–	–	–	+	+	+	+	+	+	+	+	+	+
sHo2	–	–	–	–	–	+	–	–	+	–	+	+	+	+	+	+	+	+	+	+
sHo3	–	–	–	–	–	+	–	–	+	–	+	+	+	+	+	+	+	+	+	+
sHo4	–	–	–	–	–	–	–	–	–	–	+	+	+	+	+	+	+	–	–	–
sHo5	–	–	–	–	–	–	–	–	+	–	+	+	+	+	+	+	+	+	+	+
hCa1	–	–	–	–	–	–	–	–	–	–	+	+	+	+	+	+	+	+	+	+
hCa2	–	–	–	–	–	–	–	–	–	–	+	+	+	+	+	+	+	–	+	–
hCa4	–	–	–	–	–	+	+	+	+	–	+	+	+	+	+	+	+	–	–	+
hCa5	–	–	–	–	–	–	+	–	–	–	+	+	+	+	+	+	+	–	+	+
hCa6	–	–	–	–	–	–	+	–	–	–	+	+	+	+	+	+	+	–	+	+
hCa7	–	–	–	–	–	+	+	+	–	–	+	+	+	+	+	+	+	+	+	+
hCa8	–	–	–	–	–	–	–	–	–	–	+	+	+	+	+	+	+	–	+	–
hFe1	–	–	–	–	–	–	–	–	–	–	+	+	+	+	+	+	+	+	+	+
hFe6	–	–	–	–	–	–	–	+	–	–	+	+	+	+	+	+	+	+	+	+
hFe13	–	–	–	–	–	–	–	–	–	–	+	+	+	+	+	+	+	–	–	–
hFe15	–	–	–	–	–	–	+	–	–	–	+	+	+	+	+	+	+	–	+	+
hFe19	–	–	–	–	–	+	+	+	+	+	+	+	+	+	+	+	+	+	+	+
hFe26	–	–	–	–	–	–	–	–	–	–	–	+	+	+	+	+	+	–	–	–
hFe32	–	–	–	–	–	+	+	+	+	+	+	+	+	+	+	+	+	+	+	+
hFe33	–	–	–	–	–	–	–	–	+	–	+	+	+	+	+	+	+	+	+	+
sFe1	–	–	–	–	–	–	+	+	–	–	+	+	+	+	+	+	+	+	+	+
sFe2	–	–	–	–	–	+	+	+	–	–	+	+	+	+	+	+	+	+	+	+
sFe3	–	–	–	–	–	–	+	–	–	–	+	+	+	+	+	+	+	–	–	–
sFe4	–	–	–	–	–	+	+	+	–	–	+	+	+	+	+	+	+	+	+	+
sFe10	–	–	–	–	–	+	+	+	–	–	+	+	+	+	+	+	+	+	+	+
sFe19	–	–	–	–	–	+	+	+	+	+	+	+	+	+	+	+	+	+	+	+
sFe24	–	–	–	–	–	–	–	–	–	–	–	–	–	–	–	–	–	–	–	–
sFe25	–	–	–	–	–	+	+	+	–	–	+	+	+	+	+	+	+	+	+	+
sFe26	–	–	–	–	–	+	+	+	+	+	+	+	+	+	+	+	+	+	+	+

Isolate	qacA	mphC	msrA	tetK	aac*	qacH	aad(6)	APH(3')-III	SAT-4	dfrG	fusB	fusC	vgaA	fosB1	blaZ	mupA	vgaALC
hHo1	+	–	–	–	–	–	–	–	–	–	–	–	–	–	+	–	+
hHo2	–	–	–	–	–	–	–	–	–	–	–	–	–	–	–	–	–
hHo5	+	+	+	–	–	–	–	–	–	–	–	–	–	–	–	–	–
hHo6	+	+	+	+	–	–	–	–	–	–	–	–	–	–	–	–	–
hHo10	–	–	+	–	–	–	–	–	–	–	–	–	–	–	–	–	–
hHo17	+	+	+	–	+	–	–	–	–	–	–	–	–	–	–	–	–
hHo19	+	+	+	–	–	–	–	–	–	–	–	–	–	–	–	–	–
sHo2	–	–	–	–	–	–	–	–	–	–	+	–	–	–	–	–	+
sHo3	–	–	–	–	–	–	–	–	–	–	–	–	–	–	–	–	–
sHo4	–	+	+	–	–	–	–	–	–	–	–	–	–	–	–	–	–
sHo5	+	+	+	+	–	–	–	–	–	–	+	–	–	–	–	–	–
hCa1	–	–	–	–	–	–	–	–	–	–	–	–	–	–	–	–	–
hCa2	+	+	+	–	–	–	–	–	–	–	–	–	–	–	–	–	–
hCa4	+	+	+	+	+	+	+	+	+	+	+	–	–	–	–	–	–
hCa5	+	–	+	–	–	–	–	–	–	–	–	–	–	–	–	–	+
hCa6	+	–	–	–	–	–	–	–	–	–	+	–	–	–	–	–	–
hCa7	+	+	+	+	+	+	+	+	+	+	+	–	–	–	–	–	–
hCa8	+	+	+	–	–	–	–	–	–	–	–	–	–	–	–	–	–
hFe1	+	+	–	–	–	–	–	–	–	–	–	+	–	–	–	–	–
hFe6	–	–	+	–	–	–	–	–	–	–	–	–	–	–	+	–	–
hFe13	+	–	–	–	–	+	–	–	–	–	–	–	–	–	–	–	+
hFe15	+	–	–	–	–	–	–	–	–	–	+	–	–	–	–	–	–
hFe19	–	–	–	–	–	–	–	–	–	–	–	–	–	–	–	–	–
hFe26	+	–	–	–	–	–	–	–	–	–	–	–	–	–	–	–	–
hFe32	–	–	–	–	–	–	–	–	–	–	–	–	–	–	–	–	–
hFe33	+	+	+	–	+	–	–	–	–	–	–	–	–	–	–	–	+
sFe1	+	+	+	–	+	+	+	+	+	–	–	–	–	–	–	+	+
sFe2	+	+	+	+	+	+	+	+	+	–	–	–	–	–	–	–	–
sFe3	+	–	+	–	+	–	–	–	–	–	–	+	+	–	–	–	–
sFe4	+	+	+	+	+	+	+	+	+	–	–	–	–	–	–	–	–
sFe10	+	+	+	+	+	+	+	+	+	–	–	–	–	–	–	–	–
sFe19	–	–	–	–	–	–	–	–	–	–	–	–	–	–	–	–	–
sFe24	–	–	–	–	–	–	–	–	–	–	–	–	–	–	–	–	–
sFe25	+	+	+	+	+	+	+	+	+	–	–	–	–	–	–	–	–
sFe26	–	–	–	–	–	–	–	–	–	–	–	–	–	–	–	–	–

Table S6. Distribution of acquired antimicrobial resistance determinants according to host origin and clinical status among 35 *Staphylococcus haemolyticus* genomes, with a sensitivity analysis restricted to one isolate per individual host (n = 33).

**Primary analysis:** 35 genomes									
**Determinant**	**Human (n = 11) n/N**	**Canine (n = 7) n/N**	**Feline (n = 17) n/N**	**Host p-value**	**Host FDR q-value**	**Healthy (n = 22) n/N**	**Affected (n = 13) n/N**	**Clinical status p-value**	**Clinical status FDR q-value**
mecA	0/11	2/7	10/17	0.0031	0.0324	5/22	7/13	0.0786	0.2358
blaZ/PC1 family	1/11	4/7	12/17	0.0036	0.0324	9/22	8/13	0.3053	0.6686
dfrG	0/11	2/7	0/17	0.0353	0.2118	2/22	0/13	0.5193	0.8498
fusB	2/11	3/7	1/17	0.0659	0.2966	4/22	2/13	1.0000	1.0000
Aad(6)	0/11	2/7	5/17	0.1202	0.3092	2/22	5/13	0.0754	0.2358
APH(3′)-IIIa	0/11	2/7	5/17	0.1202	0.3092	2/22	5/13	0.0754	0.2358
SAT-4	0/11	2/7	5/17	0.1202	0.3092	2/22	5/13	0.0754	0.2358
aac(6′)-Ie-aph(2″)-Ia	1/11	2/7	6/17	0.3482	0.7085	3/22	6/13	0.0505	0.2358
ANT(4′)-Ia	5/11	1/7	5/17	0.3543	0.7085	6/22	5/13	0.7077	0.9478
tetL	1/11	0/7	4/17	0.4654	0.8078	3/22	2/13	1.0000	1.0000
msrA	7/11	5/7	8/17	0.4936	0.8078	12/22	8/13	0.7372	0.9478
ermC	3/11	2/7	8/17	0.5867	0.8801	5/22	8/13	0.0328	0.2358
fusC	0/11	0/7	2/17	0.6857	0.9495	1/22	1/13	1.0000	1.0000
mphC	6/11	4/7	7/17	0.7401	0.9516	10/22	7/13	0.7332	0.9478
tetK	2/11	2/7	4/17	1.0000	1.0000	3/22	5/13	0.1163	0.2991
vgaALC	2/11	1/7	3/17	1.0000	1.0000	4/22	2/13	1.0000	1.0000
vgaA	0/11	0/7	1/17	1.0000	1.0000	0/22	1/13	0.3714	0.6686
mupA	0/11	0/7	1/17	1.0000	1.0000	0/22	1/13	0.3714	0.6686
**Sensitivity analysis:** one isolate per individual host (n = 33)									
**Determinant**	**Human (n = 11) n/N**	**Canine (n = 6) n/N**	**Feline (n = 16) n/N**	**Host p-value**	**Host FDR q-value**	**Healthy (n = 21) n/N**	**Affected (n = 12) n/N**	**Clinical status p-value**	**Clinical status FDR q-value**
mecA	0/11	1/6	10/16	0.0015	0.0269	4/21	7/12	0.0518	0.1823
blaZ/PC1 family	1/11	3/6	11/16	0.0064	0.0577	8/21	7/12	0.3005	0.6760
dfrG	0/11	1/6	0/16	0.1818	0.5455	1/21	0/12	1.0000	1.0000
fusB	2/11	2/6	1/16	0.2297	0.5907	3/21	2/12	1.0000	1.0000
Aad(6)	0/11	1/6	5/16	0.0788	0.2838	1/21	5/12	0.0159	0.0713
APH(3′)-IIIa	0/11	1/6	5/16	0.0788	0.2838	1/21	5/12	0.0159	0.0713
SAT-4	0/11	1/6	5/16	0.0788	0.2838	1/21	5/12	0.0159	0.0713
aac(6′)-Ie-aph(2″)-Ia	1/11	1/6	5/16	0.4114	0.8228	2/21	5/12	0.0709	0.1823
ANT(4′)-Ia	5/11	1/6	5/16	0.5643	0.8736	6/21	5/12	0.4713	0.7875
tetL	1/11	0/6	4/16	0.5476	0.8736	3/21	2/12	1.0000	1.0000
msrA	7/11	4/6	7/16	0.5824	0.8736	11/21	7/12	1.0000	1.0000
ermC	3/11	1/6	8/16	0.3936	0.8228	4/21	8/12	0.0100	0.0713

fusC	0/11	0/6	1/16	1.0000	1.0000	1/21	0/12	1.0000	1.0000
mphC	6/11	3/6	7/16	0.8981	1.0000	9/21	7/12	0.4813	0.7875
tetK	2/11	1/6	4/16	1.0000	1.0000	2/21	5/12	0.0709	0.1823
vgaALC	2/11	1/6	3/16	1.0000	1.0000	4/21	2/12	1.0000	1.0000
vgaA	0/11	0/6	0/16	1.0000	1.0000	0/21	0/12	1.0000	1.0000
mupA	0/11	0/6	1/16	1.0000	1.0000	0/21	1/12	0.3636	0.7273

Note: Host-associated distributions were compared using the Fisher–Freeman–Halton exact test for 3 × 2 contingency tables. Clinical-status comparisons were performed using two-sided Fisher's exact test. Benjamini–Hochberg false discovery rate correction was applied separately to the host-origin and clinical-status comparisons. FDR-adjusted $q < 0.05$ was considered statistically significant. Bold values indicate nominal $p < 0.05$ or FDR-adjusted $q < 0.05$. For the sensitivity analysis, one isolate per individual host was retained. When more than one isolate originated from the same host, the isolate with the lowest numerical isolate identifier was retained, resulting in 33 isolates representing 33 unique hosts.

Table S7. Genotype–phenotype concordance between WGS-detected antimicrobial resistance determinants and phenotypic antimicrobial susceptibility among 35 *Staphylococcus haemolyticus* isolates.

**Antimicrobial**	Phenotypic method	WGS determinants	P+/G+	P-/G-	P+/G-	P-/G+	Agreement %	Cohen's κ	McNemar p-value	Predominant discordance
Oxacillin	MIC	*mecA*	2	21	2	10	65.71	0.095	0.039	Genotype + / phenotype S
Oxacillin*	Disk diffusion	*mecA*	10	20	3	2	85.71	0.689	1.000	Phenotype R / genotype −
Erythromycin	Disk diffusion	*ermC, msrA, mphC*	18	5	2	10	65.71	0.250	0.039	Genotype + / phenotype S
Erythromycin	MIC	*ermC, msrA, mphC*	27	6	1	1	94.29	0.821	1.000	No directional predominance
Clindamycin	Disk diffusion	*ermC, vgaA, vgaALC*	13	16	1	5	82.86	0.659	0.219	Genotype + / phenotype S
Clindamycin	MIC	*ermC, vgaA, vgaALC*	11	17	0	7	80.00	0.604	0.016	Genotype + / phenotype S
Tetracycline	Disk diffusion	*tetK, tetL*	11	21	1	2	91.43	0.813	1.000	Genotype + / phenotype S
Tetracycline	MIC	*tetK, tetL*	12	17	5	1	82.86	0.655	0.219	Phenotype R / genotype −
Mupirocin	Disk diffusion	*mupA*	1	34	0	0	100.00	1.000	1.000	No directional predominance
Gentamicin	Disk diffusion	*aac(6′)-Ie-aph(2″)-Ia*	5	26	0	4	88.57	0.650	0.125	Genotype + / phenotype S
Fusidic acid	Disk diffusion	*fusB, fusC*	2	27	0	6	82.86	0.340	0.031	Genotype + / phenotype S

Note: P+/G+ = phenotypically resistant and genotype positive; P−/G− = phenotypically susceptible and genotype negative; P+/G− = phenotypically resistant and genotype negative; P−/G+ = phenotypically susceptible and genotype positive. Disk-diffusion and MIC classifications were analysed independently. For analyses based on multiple WGS determinants, genotype positivity was defined as detection of at least one of the listed determinants. McNemar's exact test was used to assess directional discordance.

* For oxacillin, MIC-based classification was considered the primary susceptibility phenotype. Disk-diffusion classification was retained as an operational comparative phenotype only and was analysed independently from MIC-based susceptibility. Its inclusion in the concordance analysis does not imply validation of oxacillin disk diffusion as a CLSI-supported primary susceptibility method for *S. haemolyticus*.

Table S8. Concordance between PCR- and WGS-based detection of antimicrobial resistance genes among 35 *Staphylococcus haemolyticus* isolates.

**Gene**	PCR positive n/N %	WGS positive n/N %	PCR+ / WGS+	PCR- / WGS-	PCR+ / WGS-	PCR- / WGS+	Agreement %	Cohen's κ	McNemar p-value	Interpretation
blaZ	19/35 (54.3%)	17/35 (48.6%)	17	16	2	0	94.29	0.886	0.500	Almost perfect agreement
mecA	12/35 (34.3%)	12/35 (34.3%)	11	22	1	1	94.29	0.873	1.000	Almost perfect agreement
tetM	0/35 (0%)	0/35 (0%)	0	35	0	0	100.00	NA	1.000	Complete negative concordance; κ not estimable because both methods were uniformly negative
aac(6′)-Ie-aph(2″)-Ia	8/35 (22.9%)	9/35 (25.7%)	8	26	0	1	97.14	0.922	1.000	Almost perfect agreement
ermC	3/35 (8.6%)	13/35 (37.1%)	3	22	0	10	71.43	0.274	0.002	Fair agreement
tetK	7/35 (20.0%)	8/35 (22.9%)	7	27	0	1	97.14	0.915	1.000	Almost perfect agreement
tetL	7/35 (20.0%)	5/35 (14.3%)	5	28	2	0	94.29	0.800	1.000	Substantial agreement
fusB	6/35 (17.1%)	6/35 (17.1%)	6	29	0	0	100.00	1.000	1.000	Almost perfect agreement
mupA	1/35 (2.9%)	1/35 (2.9%)	1	34	0	0	100.00	1.000	1.000	Almost perfect agreement

Note: WGS *blaZ* positivity was defined as detection of a *blaZ*/*PC1*-family β-lactamase determinant. For all other genes, PCR and WGS results were compared gene by gene. Cohen's κ was not estimated when both methods were uniformly negative. McNemar's exact test was used to assess asymmetry between PCR-positive/WGS-negative and PCR-negative/WGS-positive discordant pairs. Genes uniformly negative by PCR and WGS in this subset (*tetO, mecC, ermA, ermB, vanA, and vanB*) are not shown separately.

Table S9. Distribution of VFDB-associated loci among 35 *Staphylococcus haemolyticus* isolates analysed by whole-genome sequencing (WGS).

Isolate	srtC	prgB/asc1(	hasC	efaA	ecbA/fss3	ebpC	ebpB	ebpA	EF3023	bopD	esxD	esxB	esxA	essC	essB	essA	esaB	esaE	cap8P	cap9O	cap8L	cap8M	cap8D	cap8G	cap8E	cap8F	cap8J	cap8K	tufA	clpC	clpP	capC	cap8B	esaG
hHo1	–	–	–	–	–	–	–	–	–	–	–	–	–	–	–	–	–	–	–	–	–	–	+	–	–	–	–	–	+	+	+	–	+	–
hHo2	–	–	–	–	–	–	–	–	–	–	–	–	–	–	–	–	–	–	–	–	–	–	–	–	–	–	–	–	+	+	+	–	–	+
hHo5	–	–	–	–	–	–	–	–	–	–	–	–	–	–	–	–	–	–	–	–	–	–	–	–	–	–	–	–	+	+	+	+	–	–
hHo6	–	–	–	–	–	–	–	–	–	–	–	–	–	–	–	–	–	–	–	–	–	–	–	–	–	–	–	–	+	+	+	–	–	–
hHo10	–	–	–	–	–	–	–	–	–	–	–	–	–	–	–	–	–	–	–	–	–	–	–	–	–	–	–	–	+	+	+	–	–	–
hHo17	–	–	–	–	–	–	–	–	–	–	–	–	–	–	–	–	–	–	+	+	+	+	+	+	+	+	–	–	+	+	+	–	–	–
hHo19	–	–	–	–	–	–	–	–	–	–	–	–	–	–	–	–	–	–	–	–	–	–	–	–	–	–	–	–	+	+	+	–	–	+
sHo2	–	–	–	–	–	–	–	–	–	–	–	–	–	–	–	–	–	–	+	+	+	+	+	+	+	+	–	–	+	+	+	–	–	–
sHo3	–	–	–	–	–	–	–	–	–	–	–	–	–	–	–	–	–	–	+	+	+	+	+	+	+	+	–	–	+	+	+	–	–	–
sHo4	–	–	–	–	–	–	–	–	–	–	–	–	–	–	–	–	–	–	–	–	–	–	–	–	–	–	–	–	+	+	+	+	–	–
sHo5	–	–	–	–	–	–	–	–	–	–	–	–	–	–	–	–	–	–	–	–	–	–	–	–	–	–	–	–	+	+	+	–	–	+
hCa1	–	–	–	–	–	–	–	–	–	–	–	–	–	–	–	–	–	–	–	–	–	–	+	–	–	–	–	–	+	+	+	+	+	+
hCa2	–	–	–	–	–	–	–	–	–	–	–	+	+	+	+	+	+	+	–	–	–	–	+	–	–	–	–	–	+	+	+	–	+	+
hCa4	–	–	–	–	–	–	–	–	–	–	–	–	–	–	–	–	–	–	+	+	+	+	+	+	+	+	–	–	+	+	+	+	–	–
hCa5	–	–	–	–	–	–	–	–	–	–	–	–	–	–	–	–	–	–	–	–	–	–	+	–	–	–	–	–	+	+	+	–	+	–
hCa6	–	–	–	–	–	–	–	–	–	–	–	–	–	–	–	–	–	–	–	–	–	–	+	–	–	–	–	–	+	+	+	–	+	–
hCa7	–	–	–	–	–	–	–	–	–	–	–	–	–	–	–	–	–	–	+	+	+	+	+	+	+	+	–	–	+	+	+	–	–	–
hCa8	–	–	–	–	–	–	–	–	–	–	–	+	+	+	+	+	+	+	–	–	–	–	+	–	–	–	–	–	+	+	+	–	+	+
hFe1	–	–	–	–	–	–	–	–	–	–	–	–	–	–	–	–	–	–	–	–	–	–	–	–	–	–	–	–	+	+	+	–	–	+
hFe6	–	–	–	–	–	–	–	–	–	–	–	–	–	–	–	–	–	–	+	+	+	+	+	+	+	+	+	+	+	+	+	+	–	–
hFe13	–	–	–	–	–	–	–	–	–	–	–	–	–	–	–	–	–	–	–	–	–	–	–	–	–	–	–	–	+	+	+	+	–	–
hFe15	–	–	–	–	–	–	–	–	–	–	–	–	–	–	–	–	–	–	–	–	–	–	+	–	–	–	–	–	+	+	+	–	+	–
hFe19	–	–	–	–	–	–	–	–	–	–	–	–	–	–	–	–	–	–	–	–	–	–	–	–	–	–	–	–	+	+	+	–	–	–
hFe26	–	–	–	–	–	–	–	–	–	–	–	–	–	–	–	–	–	–	–	–	–	–	–	–	–	–	–	–	+	+	+	–	–	–
hFe32	–	–	–	–	–	–	–	–	–	–	–	–	–	–	–	–	–	–	–	–	–	–	–	–	–	–	–	–	+	+	+	–	–	–
hFe33	–	–	–	–	–	–	–	–	–	–	–	–	–	–	–	–	–	–	–	–	–	–	–	–	–	–	–	–	+	+	+	–	–	+
sFe1	–	–	–	–	–	–	–	–	–	–	–	–	–	–	–	–	–	–	+	+	+	+	+	+	+	+	–	–	+	+	+	–	–	–
sFe2	–	–	–	–	–	–	–	–	–	–	–	–	–	–	–	–	–	–	–	–	–	–	+	+	+	+	–	–	+	+	+	–	–	–
sFe3	–	–	–	–	–	–	–	–	–	–	–	–	–	–	–	–	–	–	–	–	–	–	+	–	–	–	–	–	+	+	+	–	+	–
sFe4	–	–	–	–	–	–	–	–	–	–	–	–	–	–	–	–	–	–	–	–	–	–	+	+	+	+	–	–	+	+	+	–	–	–
sFe10	–	–	–	–	–	–	–	–	–	–	–	–	–	–	–	–	–	–	–	–	–	–	+	+	+	+	–	–	+	+	+	–	–	–
sFe19	–	–	–	–	–	–	–	–	–	–	–	–	–	–	–	–	–	–	–	–	–	–	–	–	–	–	–	–	+	+	+	–	–	–
sFe24	–	–	–	–	–	–	–	–	–	–	–	–	–	–	–	–	–	–	–	–	–	–	–	–	–	–	–	–	+	+	+	–	–	–
sFe25	–	–	–	–	–	–	–	–	–	–	–	–	–	–	–	–	–	–	–	–	–	–	+	+	+	+	–	–	+	+	+	–	–	–
sFe26	–	–	–	–	–	–	–	–	–	–	–	–	–	–	–	–	–	–	–	–	–	–	–	–	–	–	–	–	+	+	+	–	–	–

Table S9a. Sensitivity analysis of variable VFDB-associated loci according to host origin and clinical status using one isolate per individual host (n = 33).

VFDB-associated locus	Human (n = 11) n/N	Canine (n = 6) n/N	Feline (n = 16) n/N	Host p-value	Host FDR q-value	Healthy (n = 21) n/N	Affected (n = 12) n/N	Clinical status p-value	Clinical status FDR q-value
cap8B	1/11	5/6	1/16	**0.0006**	**0.0115**	7/21	0/12	0.0319	0.1597
esxB	0/11	2/6	0/16	0.0284	0.0710	2/21	0/12	0.5227	0.7569
esxA	0/11	2/6	0/16	0.0284	0.0710	2/21	0/12	0.5227	0.7569
essC	0/11	2/6	0/16	0.0284	0.0710	2/21	0/12	0.5227	0.7569
essB	0/11	2/6	0/16	0.0284	0.0710	2/21	0/12	0.5227	0.7569
essA	0/11	2/6	0/16	0.0284	0.0710	2/21	0/12	0.5227	0.7569
esaB	0/11	2/6	0/16	0.0284	0.0710	2/21	0/12	0.5227	0.7569
esaE	0/11	2/6	0/16	0.0284	0.0710	2/21	0/12	0.5227	0.7569
cap8D	4/11	6/6	7/16	0.0379	0.0842	10/21	7/12	0.7207	0.8008
esaG	3/11	3/6	2/16	0.1457	0.2914	7/21	1/12	0.2062	0.7569
capC	2/11	2/6	2/16	0.5271	0.8052	5/21	1/12	0.3792	0.7569
cap8E	3/11	1/6	6/16	0.6924	0.8052	3/21	7/12	0.0164	0.1091
cap8F	3/11	1/6	6/16	0.6924	0.8052	3/21	7/12	0.0164	0.1091
cap8G	3/11	1/6	6/16	0.6924	0.8052	3/21	7/12	0.0164	0.1091
cap8P	3/11	1/6	2/16	0.7247	0.8052	3/21	3/12	0.6434	0.7569
cap9O	3/11	1/6	2/16	0.7247	0.8052	3/21	3/12	0.6434	0.7569
cap8L	3/11	1/6	2/16	0.7247	0.8052	3/21	3/12	0.6434	0.7569
cap8M	3/11	1/6	2/16	0.7247	0.8052	3/21	3/12	0.6434	0.7569
cap8J	0/11	0/6	1/16	1.0000	1.0000	1/21	0/12	1.0000	1.0000
cap8K	0/11	0/6	1/16	1.0000	1.0000	1/21	0/12	1.0000	1.0000

Note: Sensitivity analysis was restricted to one isolate per individual host (n = 33). Host-associated distributions were compared using the Fisher–Freeman–Halton exact test, whereas clinical-status comparisons were performed using two-sided Fisher's exact tests. Benjamini–Hochberg false discovery rate correction was applied separately to host-origin and clinical-status comparisons. FDR-adjusted $q < 0.05$ was considered statistically significant.

Table S10. Recurrent phenotypic antimicrobial resistance profiles of *S. haemolyticus* isolates determined by the disk diffusion method. Isolates are color-coded according to host origin and clinical status: hHo, clinically healthy humans; sHo, clinically affected humans; hCa, clinically healthy dogs; hFe, clinically healthy cats; sFe, clinically affected cats.

Profile ID	Phenotypic resistance profile	No. of isolates, n (%)	Isolates
**P1**	Fully susceptible	27 (23.89%)	**hHo2, hHo5, hHo10, hHo11, hHo14, hHo16, hHo17, hCa1, hCa2, hCa5, hCa8, hFe2, hFe4, hFe13, hFe16, hFe17, hFe18, hFe28, hFe29, hFe30, hFe33, sHo4, sFe3, sFe13, sFe27, sFe31, sFe38, sFe39**
**P2**	AMP–P–DA–E–TET	9 (7.96%)	**hHo1, hHo3, hHo15, hFe5, hFe22, sFe10, sFe16, sFe22, sFe30**
**P3**	AMP–AMC–P–DA–E–TET	8 (7.08%)	**hFe27, sFe5, sFe6, sFe7, sFe35, sFe37, sFe41, sFe42**
**P4**	AMP–P	6 (5.31%)	**hHo4, hFe26, sFe9, sFe20, sFe21, sFe29**
**P5**	DA–E	5 (4.42%)	**hHo9, hHo13, sHo2, sHo3, hFe20**
**P6**	E	5 (4.42%)	**hHo6, hHo19, sHo4, hFe15, sFe32**
**P7**	AMP–AMC–P–MAR–CIP–OX–DA–E–CN–TET	4 (3.54%)	**hCa7, sFe2, sFe4, sFe25**
**P8**	AMP–AMC–P–OX–DA–E–TET	4 (3.54%)	**sFe15, sFe19, sFe24, sFe26**
**P9**	FD	3 (2.65%)	**hFe7, hFe11, hFe12**
**P10**	AMP–AMC–P–DA–E	2 (1.77%)	**sFe36, sFe44**
**P11**	AMP–AMC–P–MAR–CIP–OX–DA–E–CN–SXT–TET	2 (1.77%)	**hCa4, hFe24**
**P12**	AMP–AMC–P–MAR–CIP–OX–DA–E–CN–TET–TOB	2 (1.77%)	**hFe3, sFe23**
**P13**	AMP–P–DA	2 (1.77%)	**hHo8, hFe9**
**P14**	AMP–P–DA–E	2 (1.77%)	**hFe21, hFe36**
**P15**	AMP–P–E	2 (1.77%)	**hHo18, sFe11**
**P16**	AMP–P–E–TET	2 (1.77%)	**sFe8, sFe28**
**P17**	AMP–P–OX–DA–E–TET	2 (1.77%)	**hFe31, sFe12**
**P18**	E–C	2 (1.77%)	**hFe34, hFe35**
**P19**	E–TET	2 (1.77%)	**sHo1, sHo5**
**P20**	TET	2 (1.77%)	**hHo7, sHo6**
**Legend:** hHo – healthy humans (black); sHo – sick humans (red); hCa – healthy dogs (orange); hFe – healthy cats (dark green); sFe – sick cats (dark blue).			

Note: Resistance profiles shown in this table are based on disk-diffusion results. For oxacillin, disk-diffusion categories were retained solely as operational phenotypic categories for descriptive resistance-profile analysis. MIC-based oxacillin classification was considered the primary susceptibility phenotype and was used for MDR classification.

Table S11. Heatmap showing the $\log_2$(MBC/MIC) ratio for 113 *S. haemolyticus* isolates (A. Human isolates; B and C animal isolates): tested against oxacillin (OX), clindamycin (DA), teicoplanin (TEICO), tetracycline (TET), erythromycin (E), and vancomycin (VAN): **(A)** human isolates, (B-C) animal isolates. Darker shades indicate greater differences between MBC and MIC values. Exact $\log_2$(MBC/MIC) values are shown within the cells, with MIC-based susceptibility interpretations (S, susceptible; I, susceptible, increased exposure; R, resistant) displayed in adjacent columns. White cells marked with "–" indicate strain–antimicrobial combinations for which the $\log_2$(MBC/MIC) ratio could not be determined.

A. Human isolates

	OX		DA		TEIKO		TET		E		VAN	
hHo1	1	S	2	R	2	S	2	R	2	R	2	S
hHo2	2	S	2	S	1	S	5	S	2	S	7	S
hHo3	1	S	1	S	1	S	1	R	-	R	1	S
hHo4	1	S	6	S	1	S	7	S	5	S	1	S
hHo5	1	S	3	S	1	S	1	R	2	R	1	S
hHo6	1	S	6	S	7	S	7	S	1	R	1	S
hHo7	3	S	4	S	1	S	1	I	4	S	1	S
hHo8	2	S	1	R	1	S	3	R	1	R	1	S
hHo9	2	S	1	S	3	S	4	S	2	R	1	S
hHo10	1	S	6	S	1	S	1	R	2	R	1	S
hHo11	1	S	2	S	1	S	3	S	3	S	1	S
hHo12	1	S	-	R	1	S	-	R	1	R	1	S
hHo13	2	S	2	S	1	S	5	S	2	R	1	S
hHo14	1	S	4	S	1	S	7	S	5	S	1	S
hHo15	1	S	3	S	1	S	-	R	1	R	1	S
hHo16	1	S	8	S	2	S	2	S	2	R	1	S
hHo17	1	S	6	S	3	S	4	R	2	R	1	S
hHo18	1	S	5	S	9	S	6	R	1	R	1	S
hHo19	1	S	3	S	1	S	7	S	1	R	2	S
sHo1	8	R	-	R	4	S	1	R	3	R	1	S
sHo2	1	S	4	R	1	S	7	S	1	R	5	S
sHo3	1	S	6	S	2	S	7	S	-	R	1	S
sHo4	1	S	4	S	1	S	7	S	1	R	2	S
sHo5	1	S	5	S	1	S	-	R	1	R	1	S
sHo6	3	S	6	S	1	S	-	R	5	S	1	S

B. Animal isolates — hCa and hFe

	OX		DA		TEIKO		TET		E		VAN	
hCa1	2	S	4	S	2	S	5	S	3	S	1	S
hCa2	1	S	4	S	2	S	7	S	-	R	1	S
hCa3	3	S	-	R	1	S	-	R	-	R	1	S
hCa4	1	S	-	R	2	S	-	R	1	R	6	S
hCa5	1	S	1	R	1	S	5	S	-	R	5	S
hCa6	3	S	5	S	1	S	6	S	1	S	1	S
hCa7	3	S	-	R	2	S	-	R	3	R	1	S
hCa8	1	S	6	S	6	S	7	S	2	R	1	S
hFe1	1	S	5	S	1	S	4	S	4	S	1	S
hFe2	3	S	2	S	1	S	1	I	3	S	6	S
hFe3	1	S	-	R	3	S	-	R	-	R	1	S
hFe4	1	S	5	S	1	S	5	S	1	S	1	S
hFe5	1	S	3	S	1	S	-	R	1	R	3	S
hFe6	1	S	3	S	1	S	3	S	2	R	1	S
hFe7	1	S	4	S	1	S	4	R	3	S	2	S
hFe8	1	S	5	S	1	S	4	R	4	S	1	S
hFe9	1	S	-	R	3	S	1	S	2	S	1	S
hFe10	2	S	-	R	1	S	-	R	-	R	1	S
hFe11	2	S	1	S	1	S	3	S	4	S	2	S
hFe12	3	S	3	S	1	S	6	S	5	S	1	S
hFe13	1	S	2	R	1	S	7	S	1	S	1	S
hFe14	7	R	4	I	3	S	4	S	3	S	1	S
hFe15	1	S	1	S	1	S	6	S	4	S	1	S
hFe16	3	S	3	S	2	S	5	S	2	R	2	S
hFe17	2	S	7	S	1	S	7	S	1	R	6	S
hFe18	2	S	5	R	2	S	5	S	1	R	2	S
hFe19	1	S	3	S	2	S	-	R	-	R	1	S
hFe20	2	S	5	S	3	S	5	R	3	R	3	S
hFe21	1	S	-	R	1	S	15	S	-	R	1	S
hFe22	2	R	3	S	1	S	-	R	-	R	2	S
hFe23	6	R	-	R	1	S	-	R	1	R	1	S
hFe24	6	R	5	R	1	S	-	R	-	R	1	S
hFe25	1	S	1	S	1	S	1	R	1	R	1	S
hFe26	2	S	4	S	1	S	6	S	1	S	1	S
hFe27	1	S	3	S	1	S	1	I	-	R	2	S
hFe28	4	S	2	S	2	S	5	S	1	R	2	S
hFe29	1	S	4	S	1	S	4	S	S	S	1	S
hFe30	2	S	3	S	1	S	3	S	-	R	1	S
hFe31	2	S	-	R	3	S	3	S	S	S	2	S
hFe32	3	S	3	S	3	S	-	R	1	R	1	S
hFe33	2	S	2	R	2	S	5	S	3	R	2	S
hFe34	3	S	4	R	2	S	4	S	3	R	2	S
hFe35	2	S	6	R	2	S	3	S	1	R	2	S
hFe36	3	S	6	R	3	S	3	S	2	S	2	S

log$_2$ value: 2, 4, 6, 8, 10, 12, 14

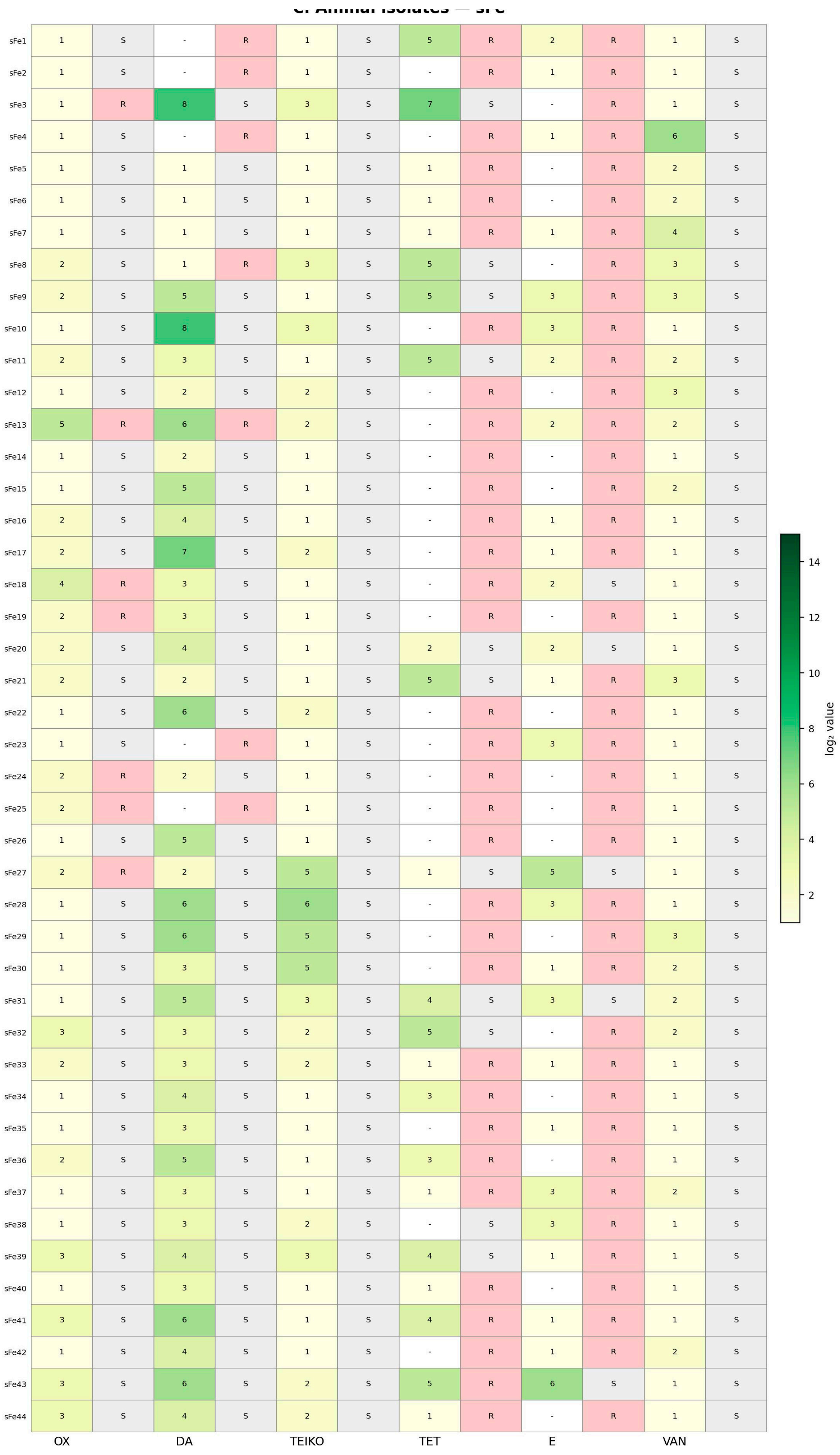
sFe1 1 S - R 1 S 5 R 2 R 1 S
sFe2 1 S - R 1 S - R 1 R 1 S
sFe3 1 R 8 S 3 S 7 S - R 1 S
sFe4 1 S - R 1 S - R 1 R 6 S
sFe5 1 S 1 S 1 S 1 R - R 2 S
sFe6 1 S 1 S 1 S 1 R - R 2 S
sFe7 1 S 1 S 1 S 1 R 1 R 4 S
sFe8 2 S 1 R 3 S 5 S - R 3 S
sFe9 2 S 5 S 1 S 5 S 3 R 3 S
sFe10 1 S 8 S 3 S - R 3 R 1 S
sFe11 2 S 3 S 1 S 5 S 2 R 2 S
sFe12 1 S 2 S 2 S - R - R 3 S
sFe13 5 R 6 R 2 S - R 2 R 2 S
sFe14 1 S 2 S 1 S - R - R 1 S
sFe15 1 S 5 S 1 S - R - R 2 S
sFe16 2 S 4 S 1 S - R 1 R 1 S
sFe17 2 S 7 S 2 S - R 1 R 1 S
sFe18 4 R 3 S 1 S - R 2 S 1 S
sFe19 2 R 3 S 1 S - R - R 1 S
sFe20 2 S 4 S 1 S 2 S 2 S 1 S
sFe21 2 S 2 S 1 S 5 S 1 R 3 S
sFe22 1 S 6 S 2 S - R - R 1 S
sFe23 1 S - R 1 S - R 3 R 1 S
sFe24 2 R 2 S 1 S - R - R 1 S
sFe25 2 R - R 1 S - R - R 1 S
sFe26 1 S 5 S 1 S - R - R 1 S
sFe27 2 R 2 S 5 S 1 S 5 S 1 S
sFe28 1 S 6 S 6 S - R 3 R 1 S
sFe29 1 S 6 S 5 S - R - R 3 S
sFe30 1 S 3 S 5 S - R 1 R 2 S
sFe31 1 S 5 S 3 S 4 S 3 S 2 S
sFe32 3 S 3 S 2 S 5 S - R 2 S
sFe33 2 S 3 S 2 S 1 R 1 R 1 S
sFe34 1 S 4 S 1 S 3 R - R 1 S
sFe35 1 S 3 S 1 S - R 1 R 1 S
sFe36 2 S 5 S 1 S 3 R - R 1 S
sFe37 1 S 3 S 1 S 1 R 3 R 2 S
sFe38 1 S 3 S 2 S - S 3 R 1 S
sFe39 3 S 4 S 3 S 4 S 1 R 1 S
sFe40 1 S 3 S 1 S 1 R - R 1 S
sFe41 3 S 6 S 1 S 4 R 1 R 1 S
sFe42 1 S 4 S 1 S - R 1 R 2 S
sFe43 3 S 6 S 2 S 5 R 6 S 1 S
sFe44 3 S 4 S 2 S 1 R - R 1 S
OX DA TEIKO TET E VAN
14 12 10 8 6 4 2
$\log_2$ value

Table S12. Genotypic antimicrobial resistance profiles of *S. haemolyticus* isolates. Isolates are color-coded according to host origin and clinical status: hHo, clinically healthy humans; sHo, clinically affected humans; hCa, clinically healthy dogs; hFe, clinically healthy cats; sFe, clinically affected cats.

Profile	Genotypic resistance profile	n (%)	Isolates
P1	No resistance genes detected	33 (29.20%)	hCa1, hCa2, hCa8, hFe1, hFe2, hFe4, hFe7, hFe13<br>hFe16, hFe17, hFe18, hFe20, hFe23, hFe33, hHo2, hHo5<br>hHo7, hHo9, hHo10, hHo11, hHo12, hHo13, hHo14, hHo16<br>hHo18, hHo19, sFe13, sFe20, sFe21, sFe27, sFe32<br>sHo4, sHo6
P2	*blaZ* + *tetL*	19 (16.81%)	hFe5, hFe22, hFe25, hFe27, hFe34, sFe5, sFe6, sFe7<br>sFe8, sFe10, sFe12, sFe14, sFe15, sFe22, sFe37<br>sFe40, sFe41, sFe43, sFe44
P3	*blaZ*	13 (11.50%)	hCa5, hFe3, hFe8, hFe9, hFe21, hFe26, hHo4, hHo8<br>sFe9, sFe11, sFe36, sFe38, sFe39
P4	*blaZ* + *mecA* + *tetL*	8 (7.08%)	hFe19, hFe32, sFe19, sFe24, sFe26, sFe28, sFe29<br>sFe30
P5	*tetL*	8 (7.08%)	hFe28, hFe29, hFe30, hHo3, sFe16, sFe17, sFe34<br>sFe35
P6	*blaZ* + *mecA* + *aac* + *tetK*	4 (3.54%)	sFe2, sFe4, sFe23, sFe25
P7	*blaZ* + *aac*	3 (2.65%)	hFe24, sFe3, sFe18
P8	*blaZ* + *mecA* + *aac* + *tetK* + *fusB*	3 (2.65%)	hCa4, hCa7, sFe31
P9	*blaZ* + *fusB*	2 (1.77%)	hCa6, hFe15
P10	*blaZ* + *tetK*	2 (1.77%)	hFe11, hFe31
P11	*blaZ* + *tetL* + *fusB*	2 (1.77%)	hFe36, sFe42
P12	*aac*	1 (0.88%)	hHo17
P13	*aac* + *fusB*	1 (0.88%)	hCa3
P14	*aac* + *tetL*	1 (0.88%)	hFe35
P15	*blaZ* + *ermC* + *tetL*	1 (0.88%)	hHo1
P16	*blaZ* + *mecA*	1 (0.88%)	hFe6
P17	*blaZ* + *mecA* + *aac*	1 (0.88%)	hFe10
P18	*blaZ* + *mecA* + *aac* + *mupA*	1 (0.88%)	sFe1
P19	*blaZ* + *mecA* + *tetK*	1 (0.88%)	hHo15
P20	*ermC*	1 (0.88%)	sHo3
P21	*ermC* + *fusB*	1 (0.88%)	sHo2
P22	*fusB*	1 (0.88%)	hFe12
P23	*mupA*	1 (0.88%)	hFe14
P24	*tetK*	1 (0.88%)	hHo6
P25	*tetK* + *fusB*	1 (0.88%)	sHo5
P26	*tetL* + *fusB* + *mupA*	1 (0.88%)	sFe33
P27	*tetM* + *tetK*	1 (0.88%)	sHo1

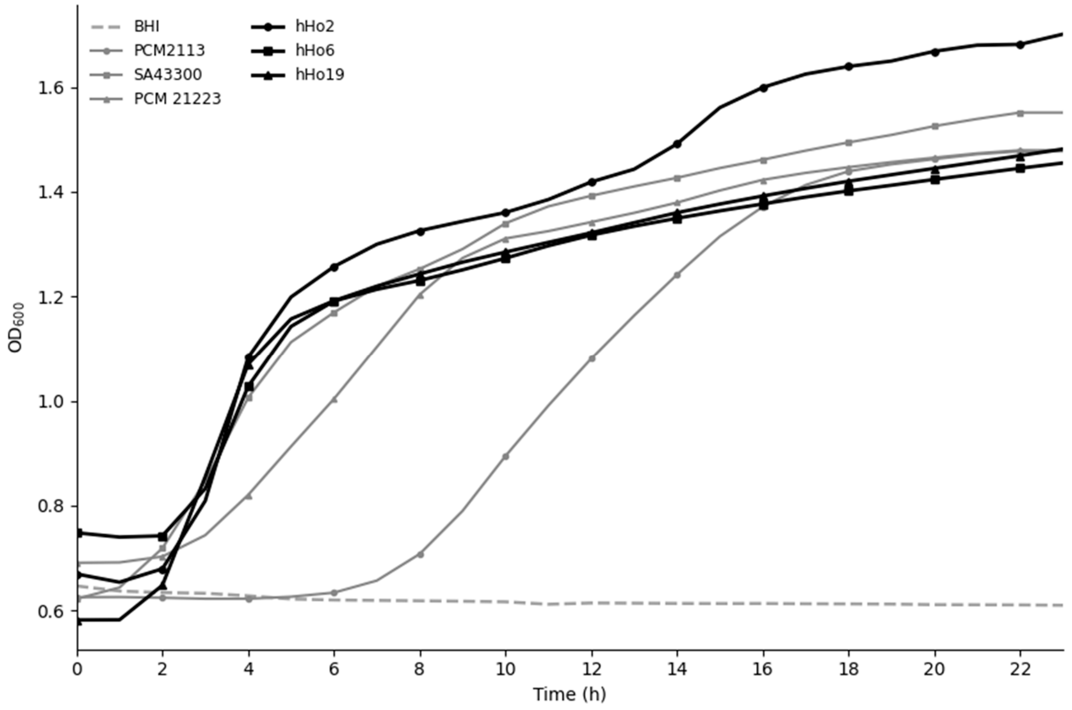

Figure S1A. Bacterial growth curves of selected hHo strains in 37 °C.

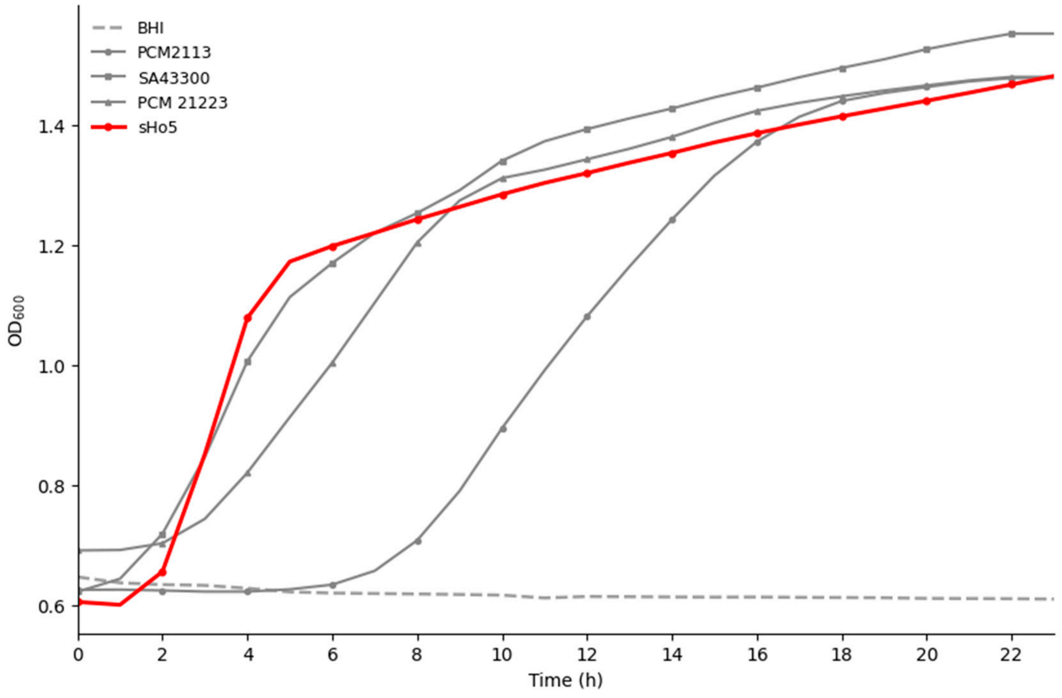

Figure S1B. Bacterial growth curves of selected sHo strains in 37 °C.

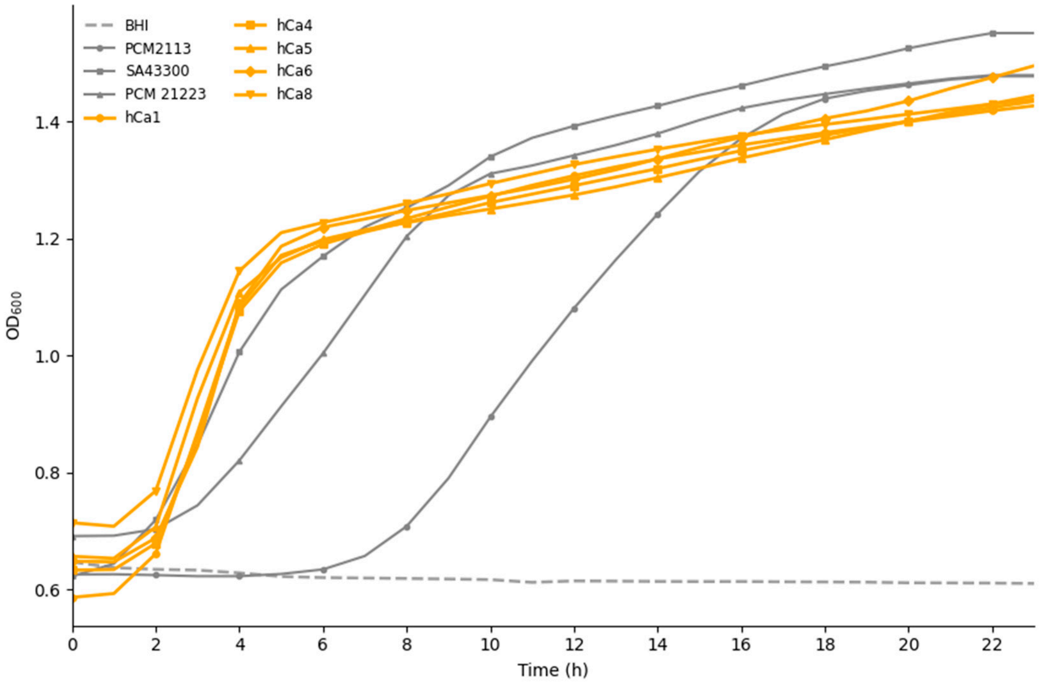

Figure S1C. Bacterial growth curves of selected hCa strains in 37 °C.

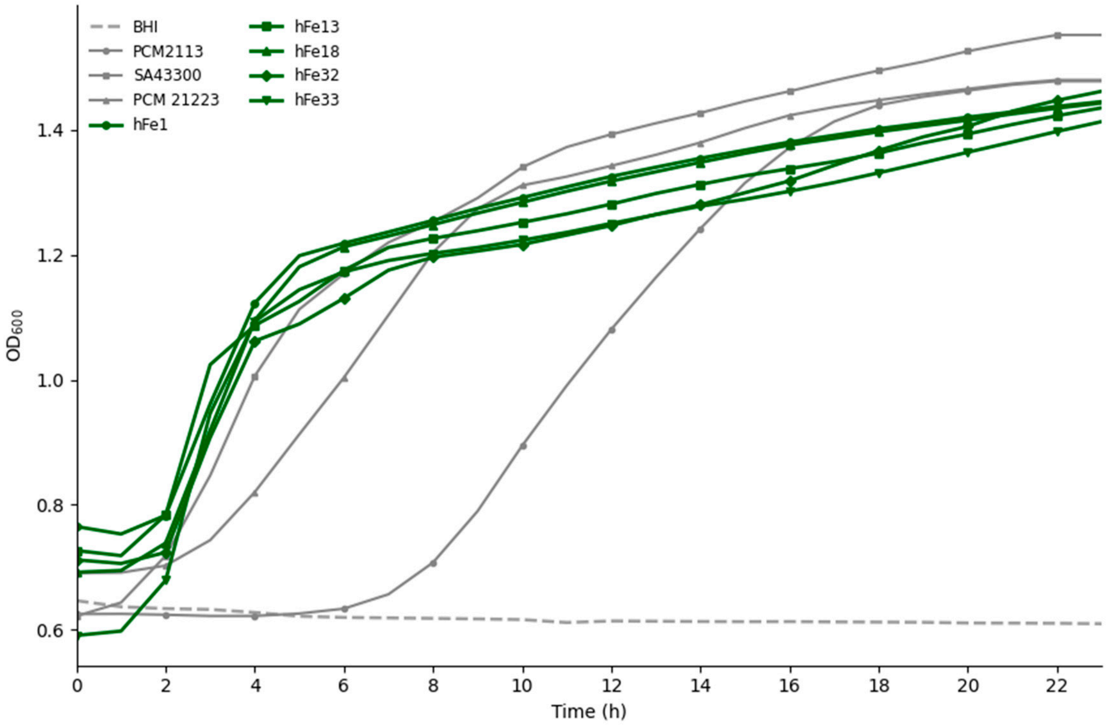

Figure S1D. Bacterial growth curves of selected hFe strains in 37 °C.

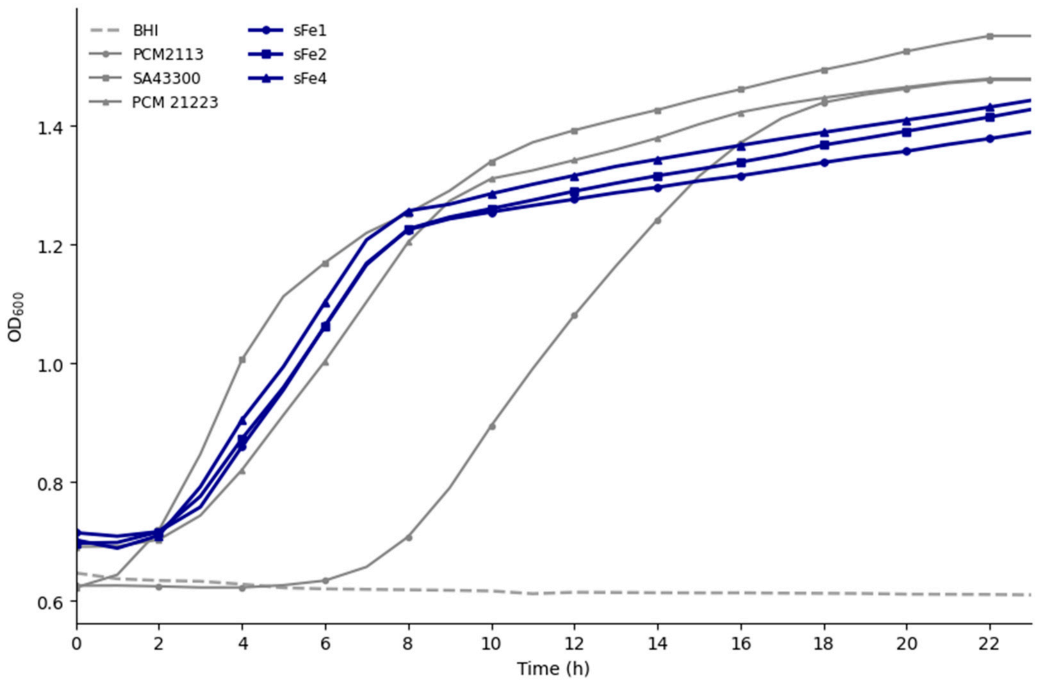

Figure S1E. Bacterial growth curves of selected sFe strains in 37 °C.

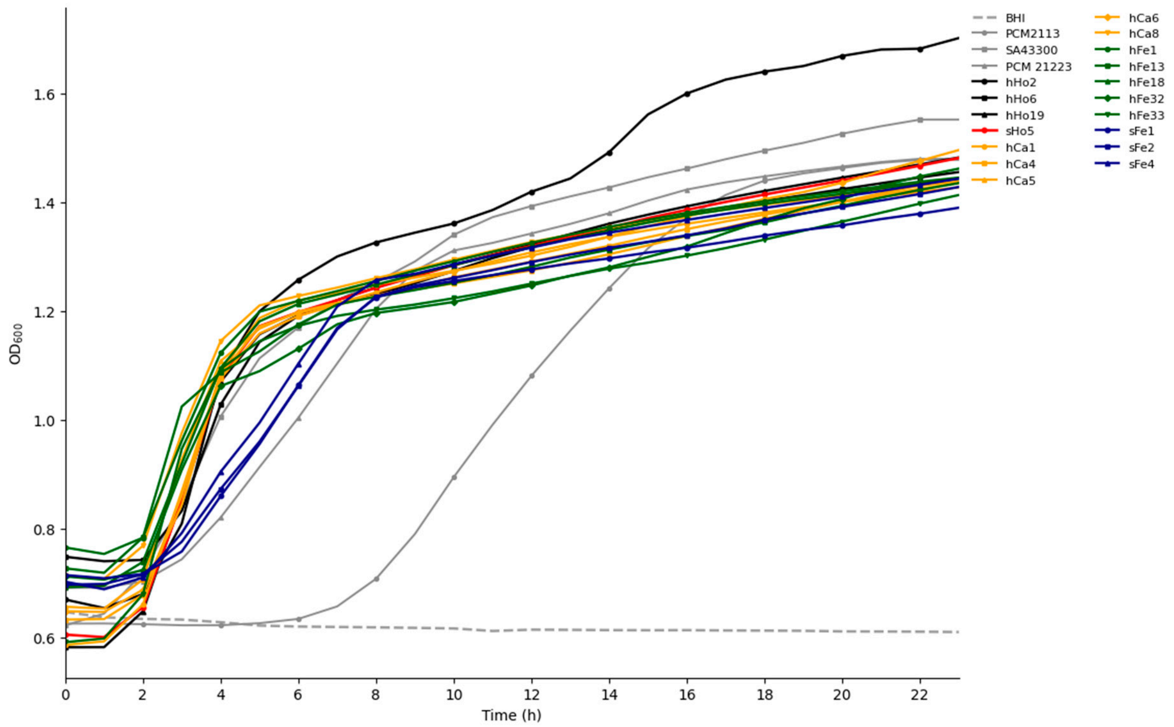

Figure S1F. Bacterial growth curves of all selected strains in 37 °C.

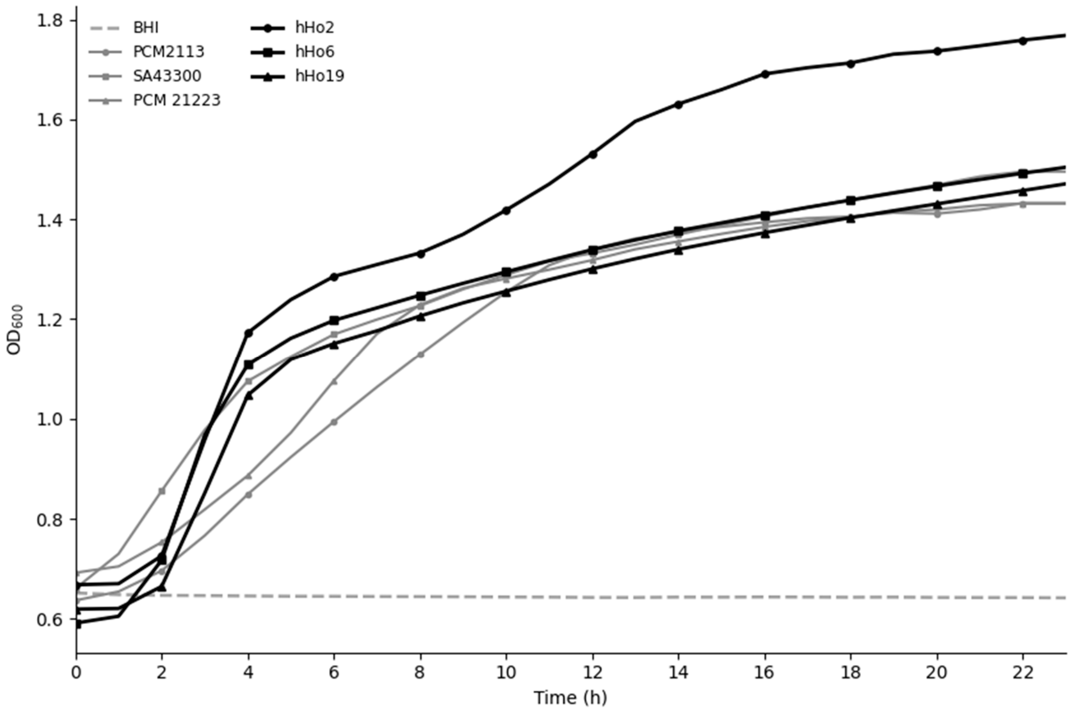

Figure S1G. Bacterial growth curves of all hHo selected strains in 38 °C.

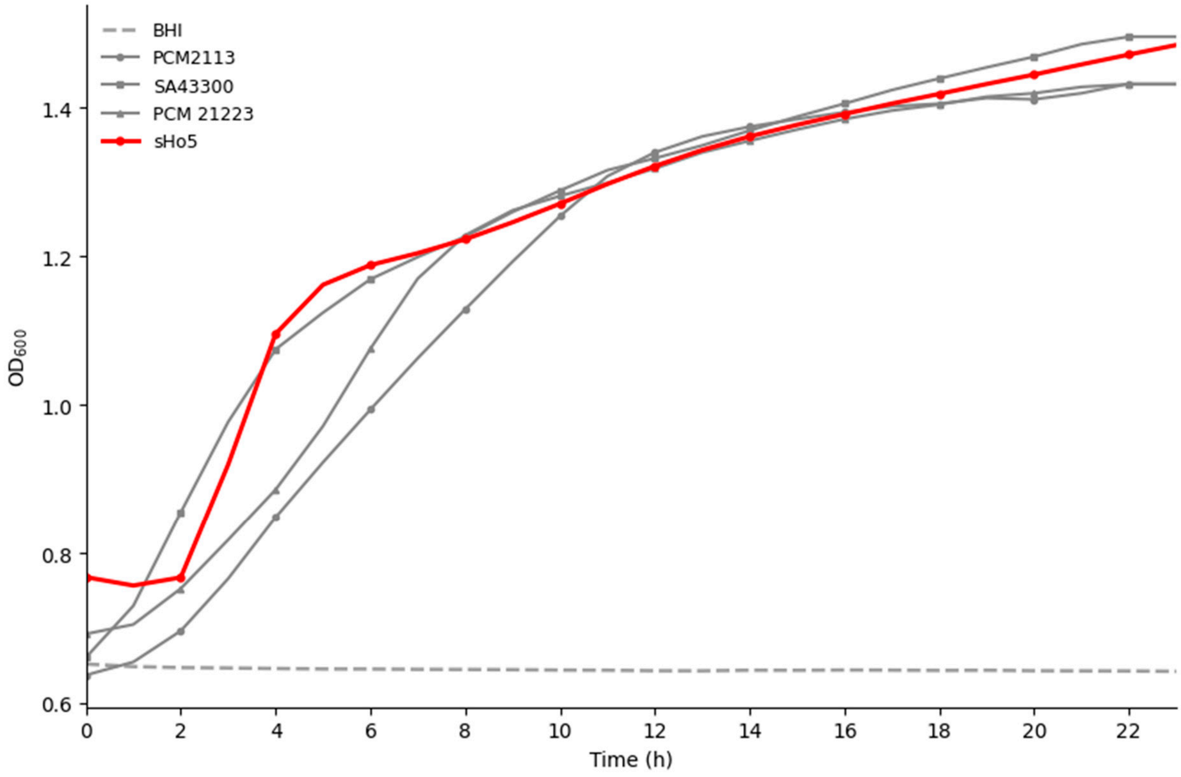

Figure S1H. Bacterial growth curves of all sHo selected strains in 38 °C.

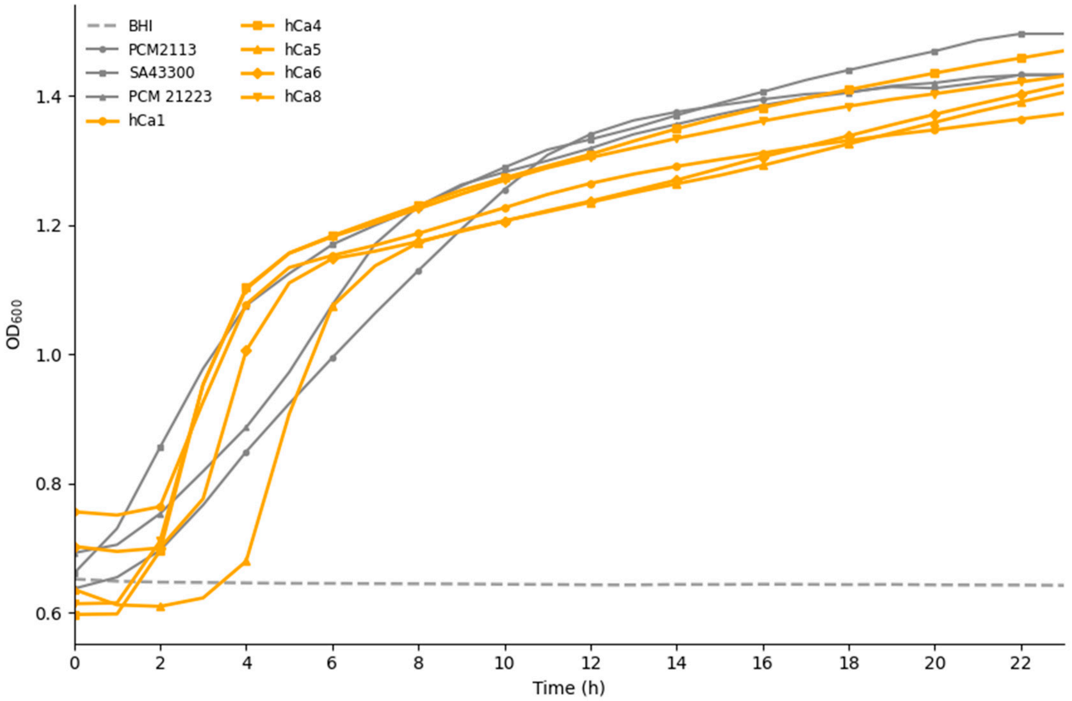

Figure S1I. Bacterial growth curves of all selected hCa strains in 38 °C.

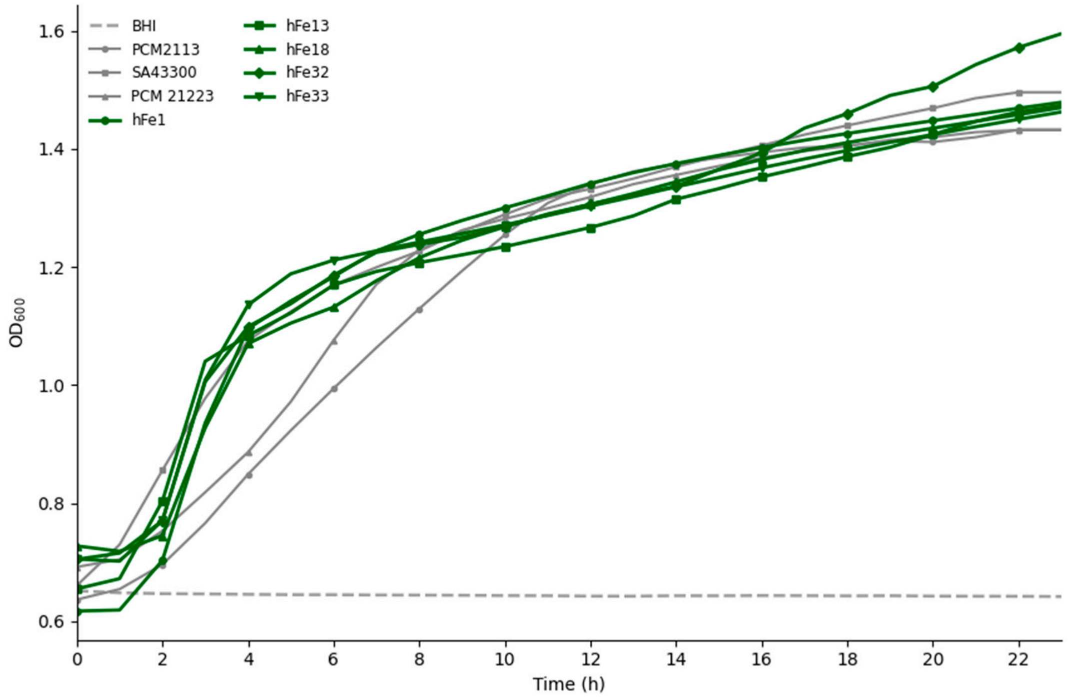

Figure S1J. Bacterial growth curves of all selected hFe strains in 38 °C.

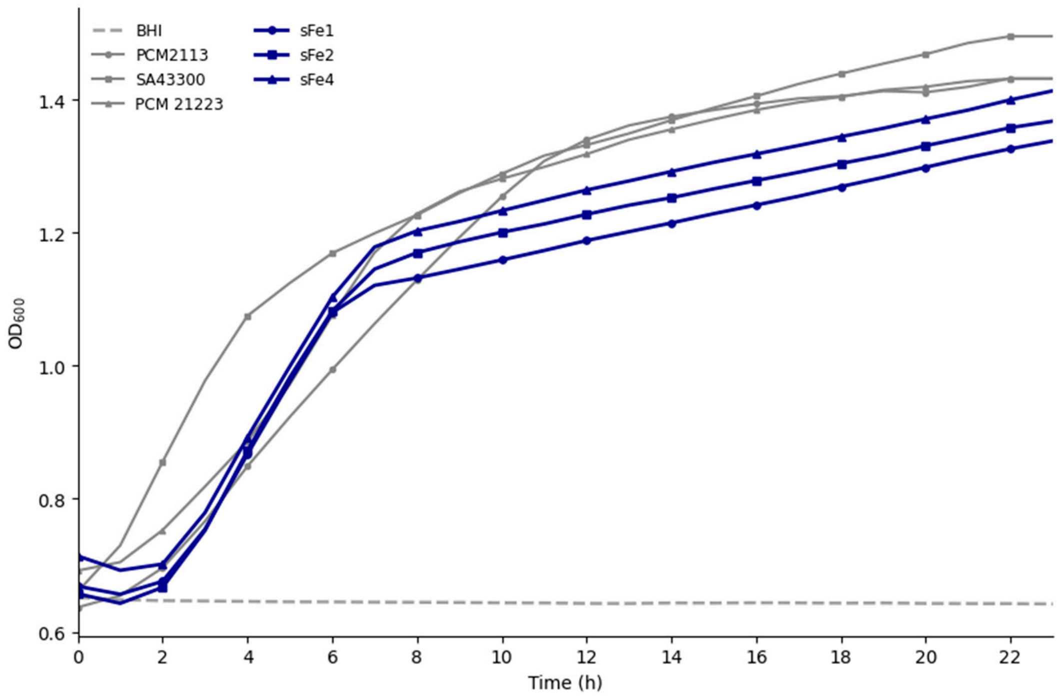

Figure S1K. Bacterial growth curves of all sFe selected strains in 38 °C.

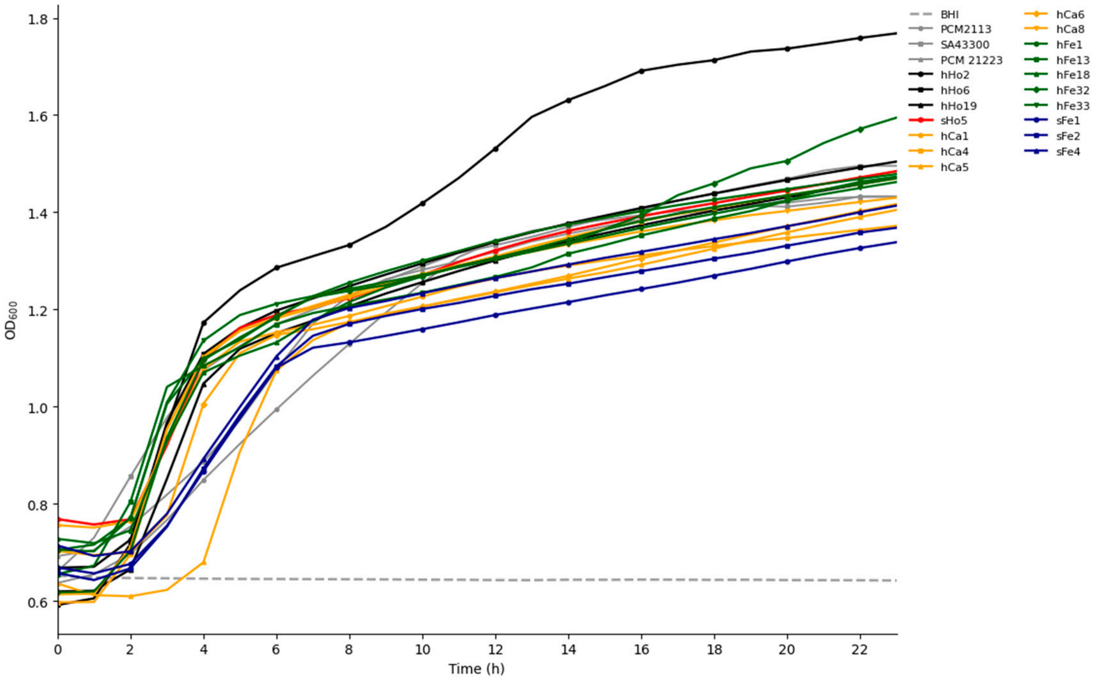

Figure S1L. Bacterial growth curves of all selected strains in 38 °C.

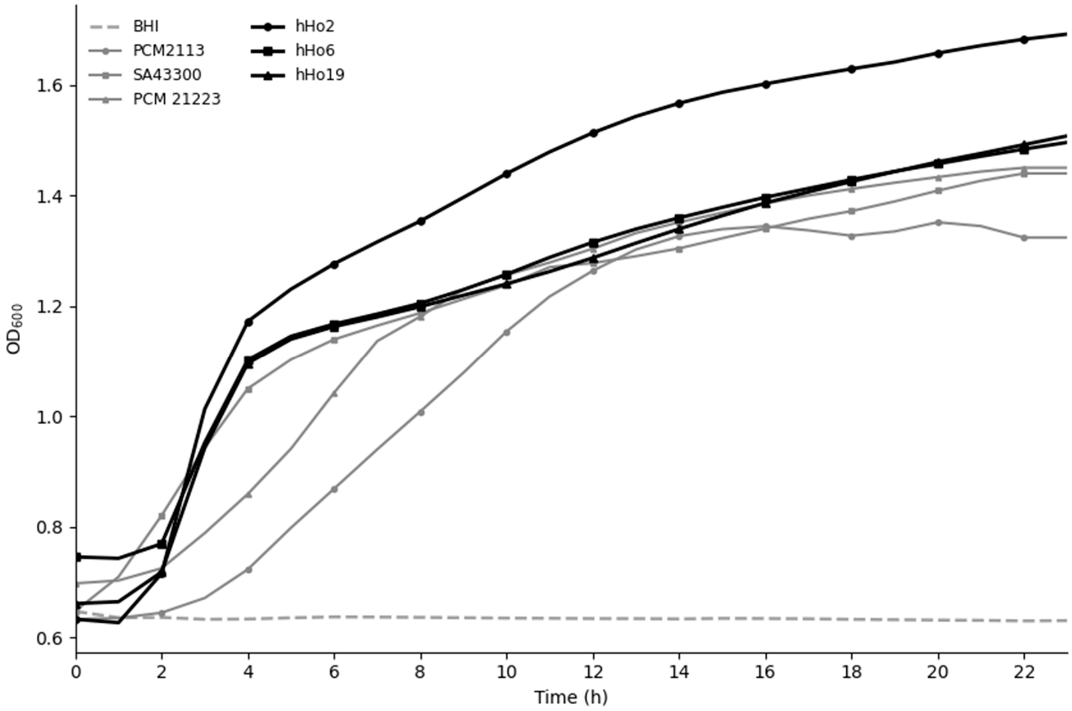

Figure S1M. Bacterial growth curves of all hHo selected strains in 39 °C.

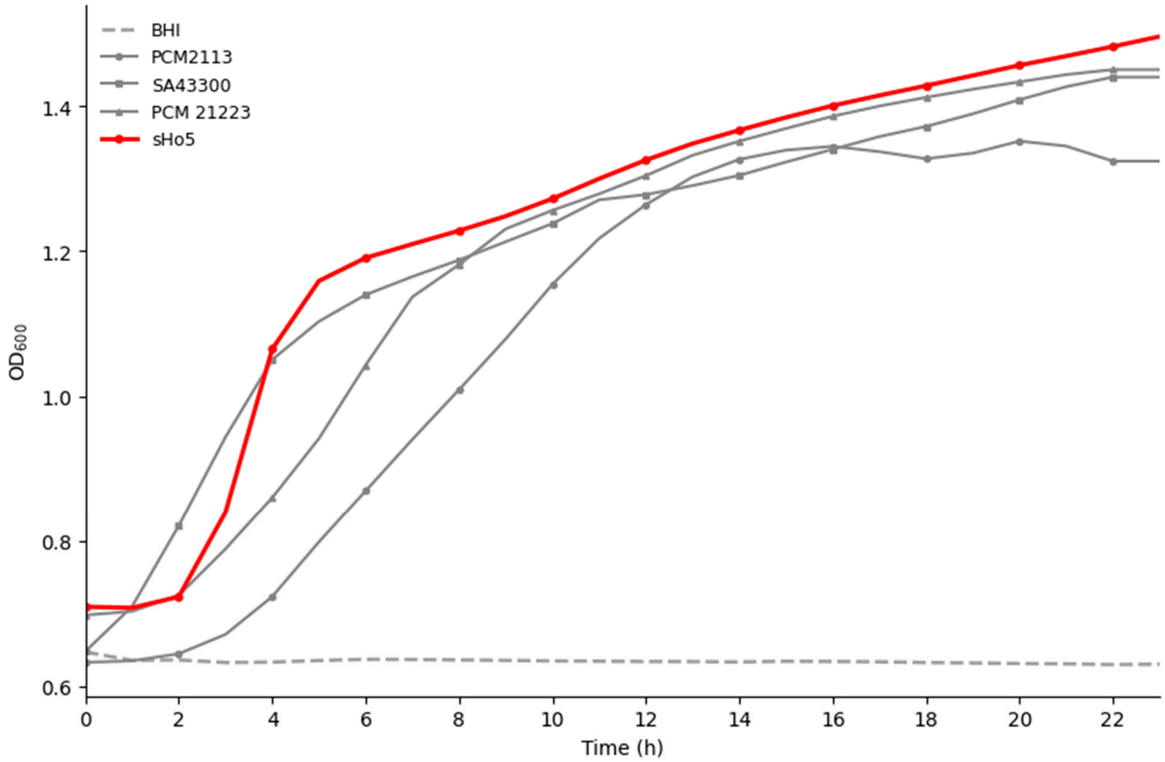

Figure S1N. Bacterial growth curves of all sHo selected strains in 39 °C.

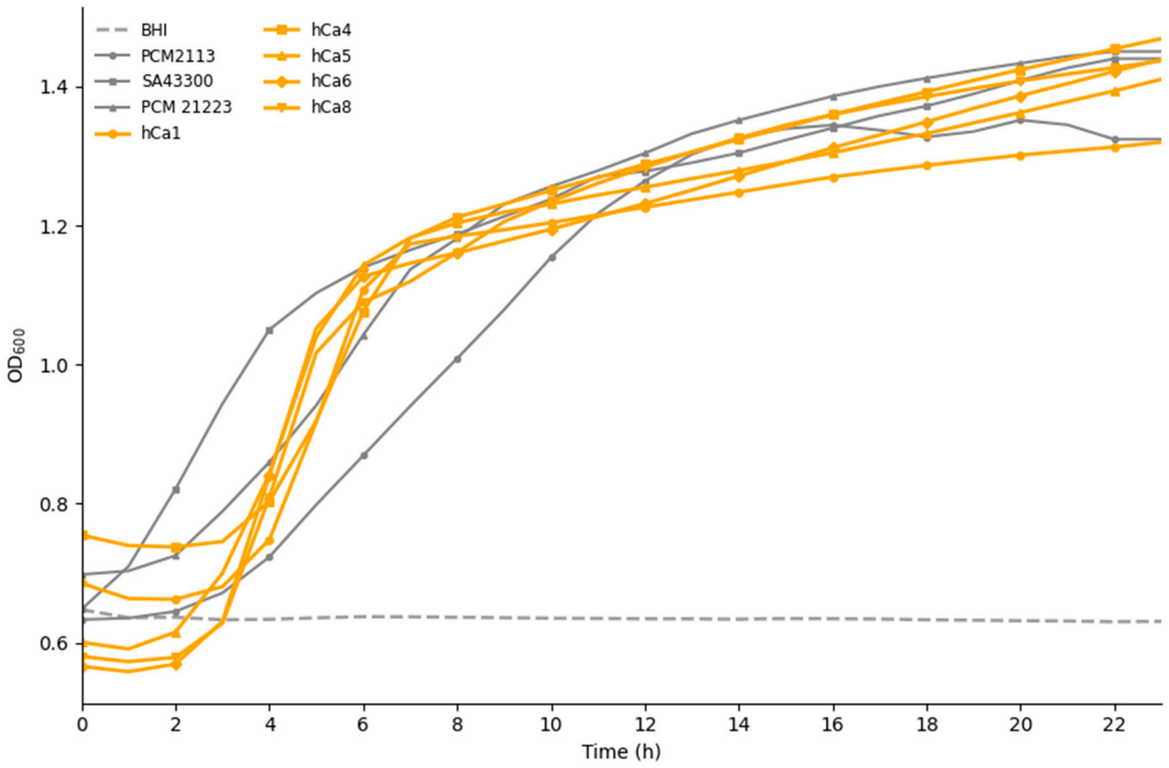

Figure S1O. Bacterial growth curves of all hCa selected strains in 39 °C.

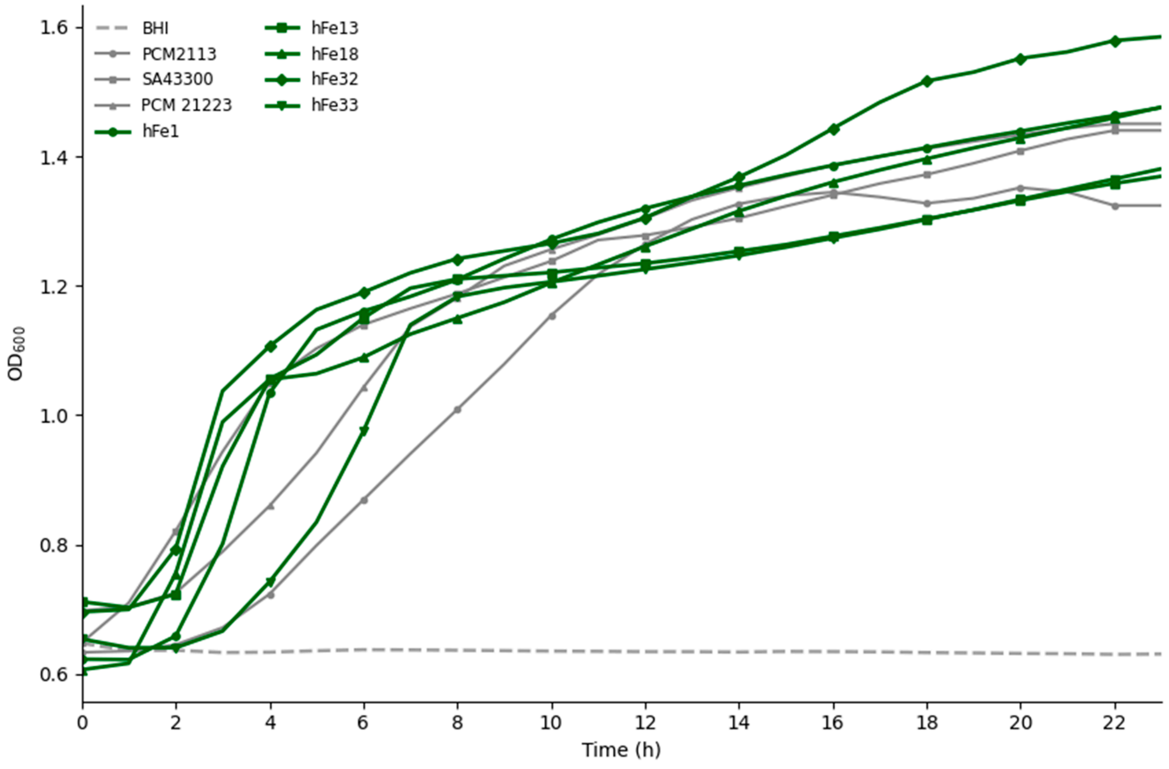

Figure S1P. Bacterial growth curves of all hFe selected strains in 39 °C.

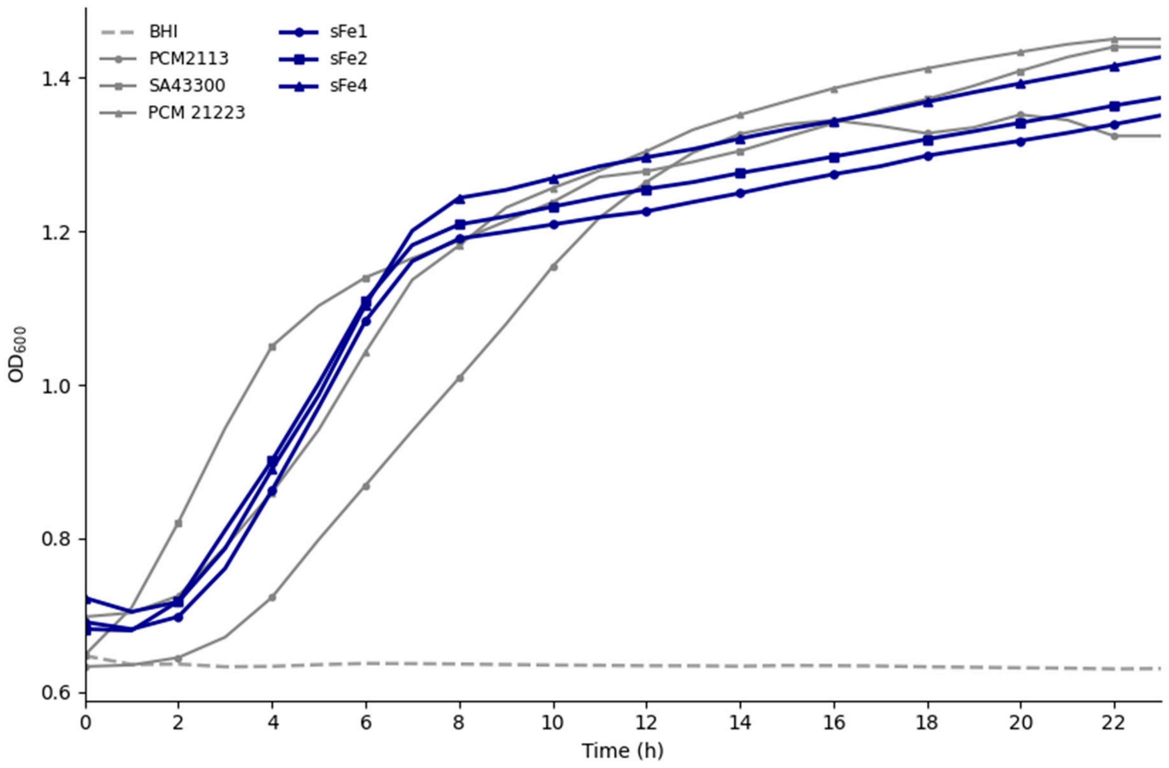

Figure S1Q. Bacterial growth curves of all sFe selected strains in 39 °C.

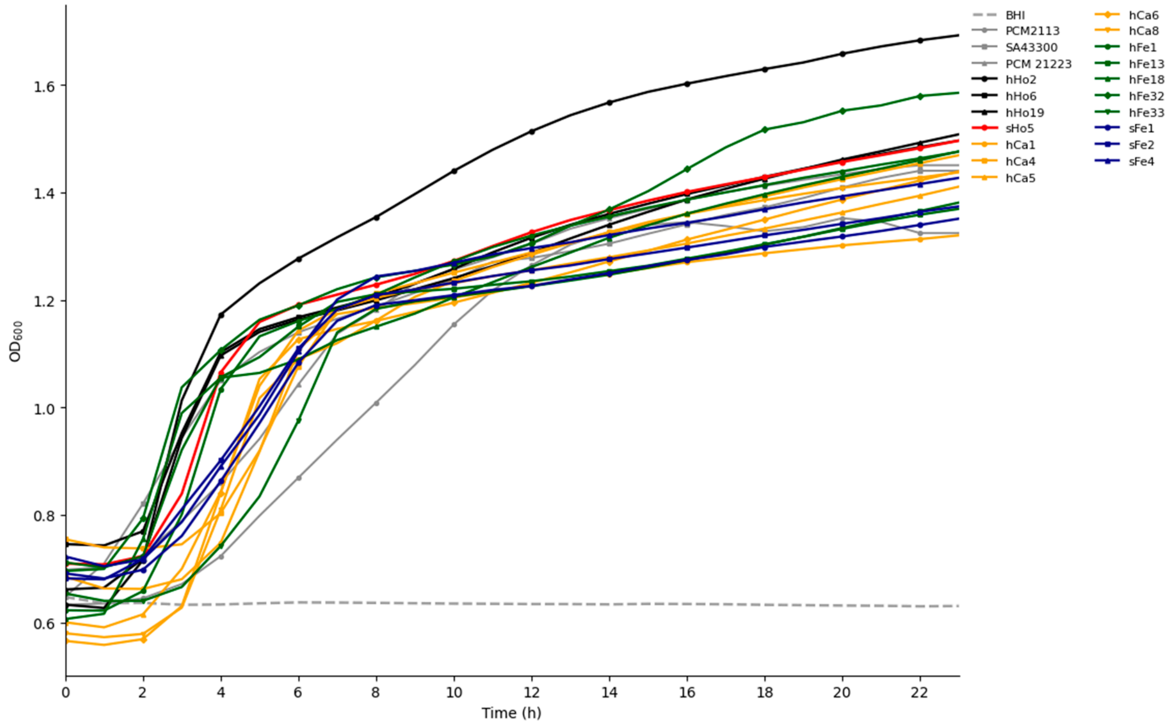

Figure S1R. Bacterial growth curves of all selected strains in 39 °C.

Note: In the growth-curve figures, the reference strain labelled as PCM 21223 corresponds to *Staphylococcus epidermidis* PCM 1869. The designation PCM 21223 resulted from a transcription error and should be read as PCM 1869.

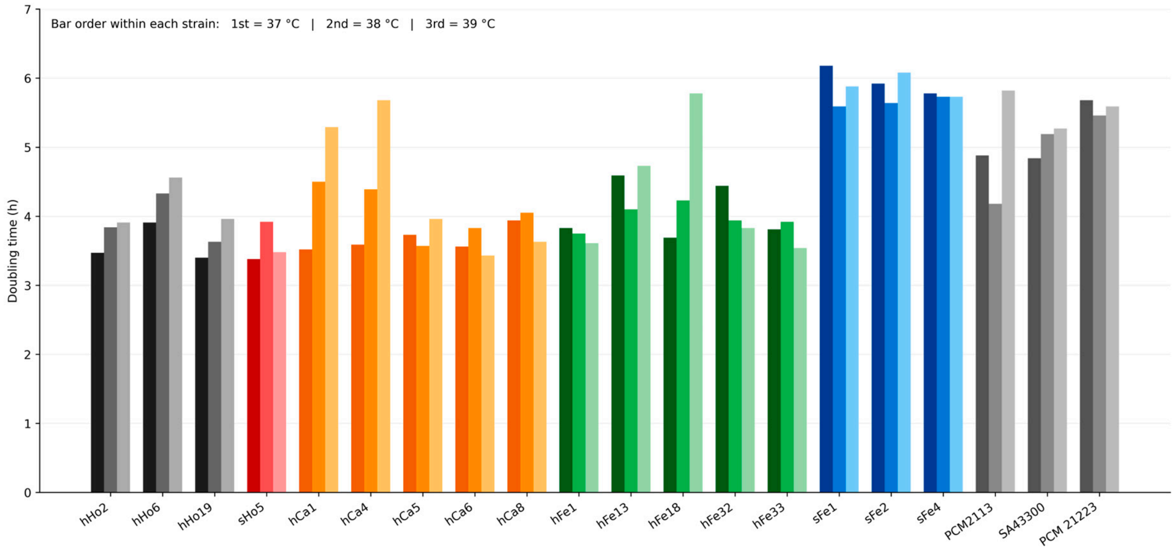

Figure S2. Comparison of doubling times (tD) of selected *S. haemolyticus* isolates and reference strains at 37 °C, 38 °C, and 39 °C. For each strain, bars from left to right represent doubling times at 37 °C, 38 °C, and 39 °C, respectively.

Table S13. Specific growth rates (μ, h^{-1}) and corresponding doubling times (tD, h) of selected *S. haemolyticus* isolates and reference strains at 37 °C, 38 °C, and 39 °C.

Isolates	μ_{37} (h^{-1})	tD 37 (h)	μ_{38} (h^{-1})	tD 38(h)	μ_{39} (h^{-1})	tD 39 (h)
**hHo2**	0.200	3.47	0.181	3.84	0.177	3.91
**hHo6**	0.177	3.91	0.160	4.33	0.152	4.56
**hHo19**	0.204	3.40	0.191	3.63	0.175	3.96
**sHo5**	0.205	3.38	0.177	3.92	0.199	3.48
**hCa1**	0.197	3.52	0.154	4.50	0.131	5.29
**hCa4**	0.193	3.59	0.158	4.39	0.122	5.68
**hCa5**	0.186	3.73	0.194	3.57	0.175	3.96
**hCa6**	0.195	3.56	0.181	3.83	0.202	3.43
**hCa8**	0.176	3.94	0.171	4.05	0.191	3.63
**hFe1**	0.181	3.83	0.185	3.75	0.192	3.61
**hFe13**	0.151	4.59	0.169	4.10	0.146	4.73
**hFe18**	0.188	3.69	0.164	4.23	0.120	5.78
**hFe32**	0.156	4.44	0.176	3.94	0.181	3.83

hFe33	0.182	3.81	0.177	3.92	0.196	3.54
sFe1	0.112	6.18	0.124	5.59	0.118	5.88
sFe2	0.117	5.92	0.123	5.64	0.114	6.08
sFe4	0.120	5.78	0.121	5.73	0.121	5.73
PCM 2113	0.142	4.88	0.166	4.18	0.119	5.82
SA43300	0.143	4.84	0.134	5.19	0.132	5.27
PCM 1869	0.122	5.68	0.127	5.46	0.124	5.59

Table S14. Biofilm production (A570) at different temperatures. Classification was based on optical density values as follows: no biofilm production (A570 ≤ 0.1856), weak biofilm production (0.1856 < A570 ≤ 0.3712), moderate biofilm production (0.3712 < A570 ≤ 0.7424), and strong biofilm production (A570 > 0.7424).

	37 °C		38 °C		39 °C	
**hHo1**	0,2619	**Weak**	0,3014	**Weak**	0,6123	**Moderate**
**hHo2**	0,3815	**Moderate**	0,4673	**Moderate**	1,0363	**Strong**
**hHo3**	0,3883	**Moderate**	0,2557	**Weak**	0,8769	**Strong**
**hHo4**	0,2276	**Weak**	0,2418	**Weak**	0,6684	**Moderate**
**hHo5**	1,3429	**Strong**	1,4729	**Strong**	1,8598	**Strong**
**hHo6**	1,9029	**Strong**	1,4878	**Strong**	3,4838	**Strong**
**hHo7**	0,3455	**Weak**	0,4229	**Moderate**	0,3901	**Moderate**
**hHo8**	0,4317	**Moderate**	0,3169	**Weak**	1,0183	**Strong**
**hHo9**	0,3260	**Weak**	0,3345	**Weak**	0,8215	**Strong**
**hHo10**	0,4208	**Moderate**	0,2474	**Weak**	0,6059	**Moderate**
**hHo11**	0,2691	**Weak**	0,219	**Weak**	0,9371	**Strong**
**hHo12**	0,3775	**Moderate**	0,2524	**Weak**	0,6442	**Moderate**
**hHo13**	0,2934	**Weak**	0,1927	**Weak**	0,52	**Moderate**
**hHo14**	0,3491	**Weak**	0,3857	**Moderate**	0,7407	**Moderate**
**hHo15**	0,3436	**Weak**	0,221	**Weak**	1,1857	**Strong**
**hHo16**	0,5904	**Moderate**	0,3368	**Weak**	1,7794	**Strong**
**hHo17**	0,2958	**Weak**	0,3821	**Moderate**	1,6009	**Strong**
**hHo18**	0,2785	**Weak**	0,227	**Weak**	0,5023	**Moderate**
**hHo19**	0,2990	**Weak**	0,5466	**Moderate**	1,0877	**Strong**
**sHo1**	0,5873	**Moderate**	0,5016	**Moderate**	1,4873	**Strong**
**sHo2**	0,4703	**Moderate**	0,4003	**Moderate**	2,0963	**Strong**
**sHo3**	0,3235	**Weak**	0,3667	**Weak**	0,7198	**Moderate**
**sHo4**	0,3224	**Weak**	0,223	**Weak**	0,7223	**Moderate**

**sHo5**	1,0213	**Strong**	0,8209	**Strong**	1,7291	**Strong**
**sHo6**	0,4318	**Moderate**	0,3817	**Moderate**	0,921	**Strong**
**hCa1**	0,4753	**Moderate**	0,3449	**Weak**	0,8539	**Strong**
**hCa2**	0,3626	**Weak**	0,586	**Moderate**	0,736	**Moderate**
**hCa3**	0,8423	**Strong**	0,6882	**Moderate**	1,4381	**Strong**
**hCa4**	0,5911	**Moderate**	0,5597	**Moderate**	2,2364	**Strong**
**hCa5**	0,3423	**Weak**	0,3775	**Moderate**	0,8128	**Strong**
**hCa6**	0,2080	**Weak**	0,2498	**Weak**	1,5291	**Strong**
**hCa7**	0,6279	**Moderate**	0,4405	**Moderate**	0,7841	**Strong**
**hCa8**	0,3761	**Moderate**	0,5186	**Moderate**	1,1624	**Strong**
**hFe1**	0,3649	**Weak**	0,3038	**Weak**	0,6116	**Moderate**
**hFe2**	0,3288	**Weak**	0,2886	**Weak**	0,7795	**Strong**
**hFe3**	0,2819	**Weak**	0,2949	**Weak**	0,5136	**Moderate**
**hFe4**	0,5240	**Moderate**	0,418	**Moderate**	0,7203	**Moderate**
**hFe5**	0,5326	**Moderate**	0,5504	**Moderate**	1,5262	**Strong**
**hFe6**	0,6048	**Moderate**	0,6591	**Moderate**	0,7577	**Strong**
**hFe7**	0,2497	**Weak**	0,2335	**Weak**	1,4073	**Strong**
**hFe8**	0,3023	**Weak**	0,3761	**Moderate**	1,0692	**Strong**
**hFe9**	0,2805	**Weak**	0,3601	**Weak**	1,7471	**Strong**
**hFe10**	1,5715	**Strong**	1,1311	**Strong**	2,1841	**Strong**
**hFe11**	0,2792	**Weak**	0,4659	**Moderate**	0,8313	**Strong**
**hFe12**	0,2136	**Weak**	0,4275	**Moderate**	0,8097	**Strong**
**hFe13**	0,7413	**Moderate**	0,4627	**Moderate**	1,1689	**Strong**
**hFe14**	0,2255	**Weak**	0,1908	**Weak**	0,7328	**Moderate**
**hFe15**	0,5677	**Moderate**	0,466	**Moderate**	0,8314	**Strong**
**hFe16**	0,2658	**Weak**	0,1973	**Weak**	0,5612	**Moderate**
**hFe17**	0,3407	**Weak**	0,3396	**Weak**	0,6998	**Moderate**
**hFe18**	0,9360	**Strong**	0,4345	**Moderate**	1,3377	**Strong**
**hFe19**	0,4311	**Moderate**	0,4506	**Moderate**	0,9177	**Strong**
**hFe20**	0,3214	**Weak**	0,2102	**Weak**	0,7908	**Strong**
**hFe21**	2,0957	**Strong**	1,277	**Strong**	3,3975	**Strong**
**hFe22**	0,3584	**Weak**	0,2589	**Weak**	0,6376	**Moderate**
**hFe23**	0,3934	**Moderate**	0,2332	**Weak**	1,2837	**Strong**
**hFe24**	1,3328	**Strong**	0,4632	**Moderate**	2,1985	**Strong**
**hFe25**	0,3414	**Weak**	0,3723	**Moderate**	1,0085	**Strong**
**hFe26**	0,3750	**Moderate**	0,1911	**Weak**	0,9964	**Strong**

**hFe27**	0,3572	**Weak**	0,6627	**Moderate**	0,6406	**Moderate**
**hFe28**	0,2491	**Weak**	0,19	**Weak**	0,7382	**Moderate**
**hFe29**	0,5047	**Moderate**	0,3225	**Weak**	1,2769	**Strong**
**hFe30**	0,3150	**Weak**	0,3579	**Weak**	1,0119	**Strong**
**hFe31**	0,5859	**Moderate**	0,6079	**Moderate**	1,115	**Strong**
**hFe32**	0,3928	**Moderate**	0,4125	**Moderate**	1,5654	**Strong**
**hFe33**	0,6841	**Moderate**	0,3191	**Weak**	1,1196	**Strong**
**hFe34**	0,3378	**Weak**	0,3216	**Weak**	0,9192	**Strong**
**hFe35**	0,3390	**Weak**	0,3262	**Weak**	0,9387	**Strong**
**hFe36**	2,6221	**Strong**	2,7382	**Strong**	2,1855	**Strong**
**sFe1**	0,4081	**Moderate**	0,2645	**Weak**	0,7022	**Moderate**
**sFe2**	2,0037	**Strong**	0,5334	**Moderate**	1,1086	**Strong**
**sFe3**	0,3669	**Weak**	0,3865	**Moderate**	0,4609	**Moderate**
**sFe4**	1,1458	**Strong**	0,5846	**Moderate**	1,7473	**Strong**
**sFe5**	0,5187	**Moderate**	0,5204	**Moderate**	1,5347	**Strong**
**sFe6**	0,6425	**Moderate**	0,4399	**Moderate**	0,683	**Moderate**
**sFe7**	0,3797	**Moderate**	0,5025	**Moderate**	0,8795	**Strong**
**sFe8**	0,4864	**Moderate**	0,5144	**Moderate**	1,1369	**Strong**
**sFe9**	0,2382	**Weak**	0,2101	**Weak**	0,6673	**Moderate**
**sFe10**	0,5553	**Moderate**	0,7749	**Strong**	1,5963	**Strong**
**sFe11**	0,5718	**Moderate**	0,5134	**Moderate**	0,9079	**Strong**
**sFe12**	0,3568	**Weak**	0,4838	**Moderate**	0,7189	**Moderate**
**sFe13**	0,3857	**Moderate**	0,3296	**Weak**	0,6845	**Moderate**
**sFe14**	0,2817	**Weak**	0,2385	**Weak**	0,6749	**Moderate**
**sFe15**	0,2847	**Weak**	0,3545	**Weak**	0,7154	**Moderate**
**sFe16**	0,3209	**Weak**	0,2821	**Weak**	0,636	**Moderate**
**sFe17**	0,3955	**Moderate**	0,443	**Moderate**	0,876	**Strong**
**sFe18**	0,3988	**Moderate**	0,3949	**Moderate**	1,2906	**Strong**
**sFe19**	0,3136	**Weak**	0,2892	**Weak**	0,7857	**Strong**
**sFe20**	0,4139	**Moderate**	0,319	**Weak**	0,7502	**Strong**
**sFe21**	0,4761	**Moderate**	0,4355	**Moderate**	0,8263	**Strong**
**sFe22**	0,5305	**Moderate**	0,6377	**Moderate**	1,9823	**Strong**
**sFe23**	0,7247	**Moderate**	0,2852	**Weak**	1,7251	**Strong**
**sFe24**	0,2605	**Weak**	0,3518	**Weak**	0,8215	**Strong**
**sFe25**	0,8858	**Strong**	0,4565	**Moderate**	1,0797	**Strong**
**sFe26**	0,4026	**Moderate**	0,5236	**Moderate**	1,5763	**Strong**

**sFe27**	0,4748	**Moderate**	0,3	**Weak**	0,8437	**Strong**
**sFe28**	0,3144	**Weak**	0,2768	**Weak**	0,8275	**Strong**
**sFe29**	0,2895	**Weak**	0,3258	**Weak**	1,2411	**Strong**
**sFe30**	0,4503	**Moderate**	0,5588	**Moderate**	1,1516	**Strong**
**sFe31**	0,3939	**Moderate**	0,6418	**Moderate**	1,0491	**Strong**
**sFe32**	0,2178	**Weak**	0,2853	**Weak**	0,5124	**Moderate**
**sFe33**	0,3377	**Weak**	0,7264	**Moderate**	2,1552	**Strong**
**sFe34**	0,2713	**Weak**	0,3309	**Weak**	0,8002	**Strong**
**sFe35**	1,0504	**Strong**	0,5255	**Moderate**	0,8811	**Strong**
**sFe36**	0,4060	**Moderate**	0,533	**Moderate**	2,1146	**Strong**
**sFe37**	0,4299	**Moderate**	0,4951	**Moderate**	0,9632	**Strong**
**sFe38**	0,4430	**Moderate**	0,4164	**Moderate**	1,8732	**Strong**
**sFe39**	0,6531	**Moderate**	1,0364	**Strong**	2,242	**Strong**
**sFe40**	0,3781	**Moderate**	0,4533	**Moderate**	0,7018	**Moderate**
**sFe41**	0,2996	**Weak**	0,3392	**Weak**	0,6564	**Moderate**
**sFe42**	0,4342	**Moderate**	0,3499	**Weak**	0,8264	**Strong**
**sFe43**	0,5297	**Moderate**	0,3607	**Weak**	1,2745	**Strong**
**sFe44**	0,2881	**Weak**	0,2086	**Weak**	1,0287	**Strong**

Table S14a. Comparison of the primary and one-isolate-per-host sensitivity analyses of quantitative biofilm production.

**Effect**	**Primary analysis, n = 113: χ^2**	**df**	**p-value**	**Sensitivity analysis, n = 84: χ^2**	**df**	**p-value**
Temperature	234.42	2	<0.001	106.26	2	<0.001
Host species	0.071	2	0.965	0.034	2	0.983
Clinical status	0.200	1	0.655	0.398	1	0.528
Temperature × host species	1.03	4	0.905	4.527	4	0.339
Temperature × clinical status	0.105	2	0.949	0.166	2	0.920

**Temperature comparison**	**Primary analysis, n = 113: estimated mean difference**	**Holm-adjusted p-value**	**Sensitivity analysis, n = 84: estimated mean difference**	**Holm-adjusted p-value**
37 °C vs 38 °C	−0.061	0.094	−0.083	0.040
37 °C vs 39 °C	−0.603	<0.001	−0.569	<0.001
38 °C vs 39 °C	−0.664	<0.001	−0.653	<0.001

Note: The primary analysis included all 113 isolates. For the one-isolate-per-host sensitivity analysis, one isolate per individual host was retained, resulting in 84 isolates representing 84 unique hosts. When more than one isolate originated from the same host, the isolate with the lowest numerical isolate identifier was retained. The same linear mixed-effects model, including the same fixed effects, interaction terms, random effect, and Holm-adjusted pairwise comparisons, was applied to both datasets.

Table S15. Detailed genotype–phenotype concordance between phenotypic antimicrobial resistance and corresponding resistance genes in *Staphylococcus haemolyticus* (n = 113). (A) contingency-table counts; (B) sensitivity, specificity, Cohen’s κ with 95% confidence intervals, and exact McNemar test results.

A.

Gene–antimicrobial	Method	TP	TN	FP	FN
blaZ–penicillin	DD	51	40	10	12
blaZ–ampicillin	DD	52	39	9	13
blaZ–AMC	DD	27	45	34	7
blaZ–oxacillin	MIC	7	47	54	5
blaZ–oxacillin	DD	20	49	41	3
mecA–oxacillin	MIC	3	85	16	9
mecA–oxacillin	DD	12	83	7	11
mecA–penicillin	DD	18	49	1	45
mecA–ampicillin	DD	17	46	2	48
mecA–AMC	DD	11	71	8	23
ermC–erythromycin	MIC	3	28	0	82
ermC–erythromycin	DD	3	47	0	63
ermC–clindamycin	MIC	2	83	1	27
ermC–clindamycin	DD	3	62	0	48
mupA–mupirocin	DD	3	110	0	0
fusB–fusidic acid	DD	2	97	10	4
aac(6′)-Ie-aph(2″)-Ia–gentamicin	DD	9	95	6	3
tetM–tetracycline	MIC	1	53	0	59
tetM–tetracycline	DD	1	63	0	49
tetK–tetracycline	MIC	9	49	4	51
tetK–tetracycline	DD	10	60	3	40
tetL–tetracycline	MIC	32	45	8	28
tetL–tetracycline	DD	30	53	10	20

B.

Gene–antimicrobial	Method	Sens. %	Spec. %	κ (95% CI)	McNemar p
blaZ–penicillin	DD	81.0	80.0	0.607 (0.460–0.754)	0.832
blaZ–ampicillin	DD	80.0	81.3	0.606 (0.459–0.753)	0.523
blaZ–AMC	DD	79.4	57.0	0.297 (0.143–0.450)	<0.001
blaZ–oxacillin	MIC	58.3	46.5	0.017 (−0.089–0.123)	<0.001
blaZ–oxacillin	DD	87.0	54.4	0.256 (0.124–0.389)	<0.001
mecA–oxacillin	MIC	25.0	84.2	0.073 (−0.130–0.276)	0.230
mecA–oxacillin	DD	52.2	92.2	0.475 (0.268–0.681)	0.481
mecA–penicillin	DD	28.6	98.0	0.244 (0.127–0.361)	<0.001
mecA–ampicillin	DD	26.2	95.8	0.195 (0.081–0.310)	<0.001
mecA–AMC	DD	32.4	89.9	0.254 (0.065–0.443)	0.011
ermC–erythromycin	MIC	3.5	100.0	0.018 (−0.003–0.039)	<0.001
ermC–erythromycin	DD	4.5	100.0	0.038 (−0.005–0.081)	<0.001
ermC–clindamycin	MIC	6.9	98.8	0.081 (−0.051–0.213)	<0.001

ermC–clindamycin	DD	5.9	100.0	0.064 (−0.007–0.135)	<0.001
mupA–mupirocin	DD	100.0	100.0	1.000†	1.000
fusB–fusidic acid	DD	33.3	90.7	0.163 (−0.100–0.426)	0.180
aac(6′)-Ie-aph(2″)-Ia–gentamicin	DD	75.0	94.1	0.622 (0.397–0.847)	0.508
tetM–tetracycline	MIC	1.7	100.0	0.016 (−0.015–0.046)	<0.001
tetM–tetracycline	DD	2.0	100.0	0.022 (−0.021–0.065)	<0.001
tetK–tetracycline	MIC	15.0	92.5	0.071 (−0.039–0.181)	<0.001
tetK–tetracycline	DD	20.0	95.2	0.165 (0.032–0.298)	<0.001
tetL–tetracycline	MIC	53.3	84.9	0.374 (0.215–0.533)	0.001
tetL–tetracycline	DD	60.0	84.1	0.451 (0.286–0.615)	0.099

Note: DD, disk diffusion; MIC, minimum inhibitory concentration; AMC, amoxicillin/clavulanic acid; TP, genotype-positive/phenotype-resistant; TN, genotype-negative/phenotype-susceptible; FP, genotype-positive/phenotype-susceptible; FN, genotype-negative/phenotype-resistant; Sens., sensitivity; Spec., specificity; κ, Cohen's kappa coefficient; CI, confidence interval. Sensitivity was calculated as TP/(TP + FN), and specificity as TN/(TN + FP). † Complete concordance; this result should be interpreted cautiously because only three isolates were *mupA*-positive. Concordance analysis was not applicable for *mecC, ermA, ermB, vanA, vanB,* and *tetO* because these genes were not detected in the analysed isolates. For oxacillin, MIC-based classification was considered the primary susceptibility phenotype, whereas disk-diffusion classification was retained only as an operational comparative phenotype.

Table S16. Antimicrobial susceptibility testing criteria used for *Staphylococcus haemolyticus*, including disc contents, interpretive thresholds, breakpoint sources, and species applicability.

**Antimicrobial agent**	**Abbreviation**	**Disk content**	**Interpretive criterion**	**Source / basis**	**Comment**
Amoxicillin/clavulanic acid	AMC	20/10 µg	S ≥20 mm; R ≤19 mm	[51]	Historical CLSI breakpoint for *Staphylococcus spp.*; not specific for *S. haemolyticus*.
Ampicillin	AMP	10 µg	S ≥29 mm; R ≤28 mm	[52]	Historical CLSI breakpoint for *Staphylococcus spp.*; not specific for *S. haemolyticus*.
Penicillin G	P	10 U	S ≥29 mm; R ≤28 mm	[49]	Breakpoint applicable to staphylococci; not specific for *S. haemolyticus*.
Oxacillin*	OX	1 µg	S ≥18 mm; R ≤17 mm	[49]	Operational disk-diffusion criterion retained for comparative phenotypic analyses; see footnote.
Mupirocin	MUP	200 µg	S ≥30 mm; I 18–29 mm; R <18 mm	[54]	*S. aureus* criterion used as a surrogate; no *S. haemolyticus*-specific breakpoint available.
Marbofloxacin	MAR	5 µg	S ≥23 mm; I 16–22 mm; R ≤16 mm	[50]	Veterinary breakpoint for *Staphylococcus spp.*; not specific for *S. haemolyticus*.

Ciprofloxacin	CIP	5 µg	S ≥21 mm; I 16–20 mm; R ≤15 mm	[49]	Breakpoint applicable to staphylococci; not specific for *S. haemolyticus*.
Linezolid	LZD	30 µg	S ≥26 mm; I 23–25 mm; R ≤22 mm	[49]	Breakpoint applicable to staphylococci; not specific for *S. haemolyticus*.
Tigecycline	TGC	15 µg	S ≥26 mm; R ≤25 mm	[55]	Published disk-diffusion criterion for *Staphylococcus spp.*; not specific for *S. haemolyticus*.
Erythromycin	E	15 µg	S ≥23 mm; I 14–22 mm; R ≤13 mm	[49]	Breakpoint applicable to staphylococci; not specific for *S. haemolyticus*.
Clindamycin	DA	2 µg	S ≥21 mm; I 15–20 mm; R ≤14 mm	[49]	Breakpoint applicable to staphylococci; not specific for *S. haemolyticus*.
Tetracycline	TET	30 µg	S ≥19 mm; I 15–18 mm; R ≤14 mm	[49]	Breakpoint applicable to staphylococci; not specific for *S. haemolyticus*.
Tobramycin	TOB	10 µg	S ≥15 mm; I 13–14 mm; R ≤12 mm	[53]	Historical breakpoint for staphylococci; no longer included in current M100; not specific for *S. haemolyticus*.
Fusidic acid	FD	10 µg	S ≥24 mm; R <24 mm	[54]	Breakpoint applicable to staphylococci; not specific for *S. haemolyticus*.

Chloramphenicol	C	30 µg	S ≥18 mm; I 13–17 mm; R ≤12 mm	[49]	Breakpoint applicable to staphylococci; not specific for *S. haemolyticus*.
Gentamicin	CN	10 µg	S ≥15 mm; I 13–14 mm; R ≤12 mm	[49]	Breakpoint applicable to staphylococci; not specific for *S. haemolyticus*.
Trimethoprim/sulfamethoxazole	SXT	1.25/23.75 µg	S ≥16 mm; I 11–15 mm; R ≤10 mm	[49]	Breakpoint applicable to staphylococci; not specific for *S. haemolyticus*.
Rifampin	RD	5 µg	S ≥20 mm; I 17–19 mm; R ≤16 mm	[49]	Breakpoint applicable to staphylococci; not specific for *S. haemolyticus*.

Note: S, susceptible; I, intermediate; R, resistant. Where no validated *S. haemolyticus*-specific breakpoint was available, interpretive criteria established for *Staphylococcus spp.* or another staphylococcal species were applied as indicated. Consequently, resistance classifications based on extrapolated or historical criteria should be interpreted cautiously and represent operational susceptibility categories rather than species-specific clinical resistance breakpoints.

* For oxacillin, the disk-diffusion criterion was retained solely as an operational phenotypic classification for descriptive and comparative analyses. Oxacillin disk diffusion is not a CLSI-supported primary susceptibility method for *S. haemolyticus*; MIC-based classification was therefore assessed independently and considered the primary susceptibility phenotype. The use of this operational disk-diffusion category does not imply validation of the method for clinical susceptibility interpretation in *S. haemolyticus*.

Table S17. Primer and probe sequences, amplification conditions, and expected amplicon sizes used for PCR-based detection of antimicrobial resistance genes

Gene	Function and method	Primer pair	Sequence 5'-3'	Amplicon size (bp)	Amplification cycle	Reference
blaZ	β-lactamase; penicillin resistance<br>Probe-based qPCR	*blaZ-F*	GCTTTAAAAGAACTTATTGAGGCTTCA	-	95 °C for 3 min; 40 cycles of 95 °C for 10 s, **TA 60 °C** for 30 s	[56]
		blaZ-R	CCACCGATYTCKTTTATAATTT			
		blaZ-Probe (6-FAM/BHQ-1)	AGTGATAATACAGCAAACAA			
mecA	PBP2a; methicillin resistance<br>Probe-based qPCR	*mecA-F*	GGCATGAGTAACGAAGAATATAATAAATTAACCG	97	95 °C for 3 min; 40 cycles of 95 °C for 10 s, **TA 55 °C** for 30 s	[57]
		mecA-R	TTGAGTTGAACCTGGTGAAGTTGT			
		mecA-Probe (HEX/BHQ-1)	CTGCTCAACAAGTTCC			
mecC	PBP2a; methicillin resistance<br>Conventional PCR	*mecC-F*	TCACCAGGTTCAAC[Y]CAAAA	356	94 °C for 5 min; 30 cycles of 94 °C for 1 min, **TA 55 °C** for 1 min, 72 °C for 2 min; 72 °C for 7 min	[61]
		mecC-R	CCTGAATC[W]GCTAATAATATTTC			
aac(6')-Ie-aph(2'')-Ia	Aminoglycoside-modifying enzyme; aminoglycoside resistance<br>Conventional PCR	*aac-F*	GAGCAATAAGGGCATACCAAAAATC	480	94 °C for 4 min; 35 cycles of 94 °C for 30 s, **TA 55 °C** for 30 s, 72 °C for 30 s; 72 °C for 7 min	[62]
		aac-R	CCGTGCATTTGTCTTAAAAAACTGG			
ermA	23S rRNA methyltransferase; MLSB resistance<br>Probe-based qPCR	*ermA-F*	TCAGTTACTGCTATAGAAATTGATGGAG	-	95 °C for 3 min; 40 cycles of 95 °C for 10 s, **TA 60 °C** for 30 s	[58]
		ermA-R	ATACAGAGTCTACACTTGGCTTAGG			
		ermA-Probe (6-FAM/BHQ-1)	AGTGACTAAAGAAGCGGTAAACCCCTCTGA			
ermB	23S rRNA methyltransferase; MLSB resistance<br>Probe-based qPCR	*ermB-F*	TTGGATATTCACCGAACACTAGGG	-	95 °C for 3 min; 40 cycles of 95 °C for 10 s, **TA 60 °C** for 30 s	[58]
		ermB-R	ATAGACAATACTTGCTCATAAGTAACGG			
		ermB-Probe (6-FAM/BHQ-1)	TGCTCTTGCACACTCAAGTCTCGATTCAGC			
ermC	23S rRNA methyltransferase; MLSB resistance<br>Probe-based qPCR	*ermC-F*	GACAATTATAAGATTAAATGAACATGATAATATC	-	95 °C for 3 min; 40 cycles of 95 °C for 10 s, **TA 60 °C** for 30 s	[58]
		ermC-R	AAACAATTTTGCGTATTATATCCGTAC			
		ermC-Probe (HEX/BHQ-1)	AAATCGGCTCAGGAAAAGGGCATTTTACCC			
tetK	Efflux pump; tetracycline resistance<br>Probe-based qPCR	*tetK-F*	CTAACCCTTCACCTAAAGCTAC	197	95 °C for 3 min; 40 cycles of 95 °C for 10 s, **TA 60 °C** for 30 s	[59]
		tetK-R	CTGTCTTGGTTCATTGATTGC			
		tetK-Probe (6-FAM/BHQ-1)	AGCTACAACCACCATAATCAGTGAAGGGAA			
tetL		*tetL-F*	GGTTTTGAACGTCTCATTACCTGAT	-	95 °C for 3 min; 40 cycles of 95 °C for 10 s, **TA 60 °C** for 30 s	[60]
		tetL-R	CCAATGGAAAAGGTTAACATAAAGG			

	Efflux pump; tetracycline resistance<br>Probe-based qPCR	*tetL-Probe (HEX/BHQ-1)*	CCACCTGCGAGTACAAACTGGGTGAAC			
tetM	Ribosomal protection protein; tetracycline resistance<br>Probe-based qPCR	*tetM-F*	GGTTTCTCTTGGATACTTAAATCAATCR	-	95 °C for 3 min; 40 cycles of 95 °C for 10 s, **TA 60 °C** for 30 s	[60]
		tetM-R	CCAACCATAYAATCCTTGTTCRC			
		tetM-Probe (HEX/BHQ-1)	ATGCAGTTATGGARGGGATACGCTATGGY			
tetO	Ribosomal protection protein; tetracycline resistance<br>Probe-based qPCR	*tetO-F*	AAGAAAACAGGAGATTCCAAAACG	-	95 °C for 3 min; 40 cycles of 95 °C for 10 s, **TA 60 °C** for 30 s	[60]
		tetO-R	CGAGTCCCCAGATTGTTTTTAGC			
		tetO-Probe (6-FAM/BHQ-1)	ACGTTATTTCCCGTTTATCACGG			
fusB	Ribosomal protection protein; fusidic acid resistance<br>Conventional PCR	*fusB-F*	CCGTCAAAGTTATTCAATCG	492	94 °C for 4 min; 35 cycles of 94 °C for 30 s, **TA 55 °C** for 30 s, 72 °C for 30 s; 72 °C for 7 min	[63]
		fusB-R	ACAATGAATGCTATCTCGACA			
vanA	Altered peptidoglycan precursors; glycopeptide resistance<br>Conventional PCR	*vanA-F*	GGGAAAACGACAATTGC	732	94 °C for 5 min; 30 cycles of 94 °C for 1 min, **TA 52 °C** for 1 min, 72 °C for 2 min; 72 °C for 7 min	[64]
		vanA-R	GTACAATGCGGCCGTTA			
vanB	Altered peptidoglycan precursors; glycopeptide resistance<br>Conventional PCR	*vanB-F*	ATGGGAAGCCGATAGTC	635	94 °C for 5 min; 30 cycles of 94 °C for 1 min, **TA 52 °C** for 1 min, 72 °C for 2 min; 72 °C for 7 min	[65]
		vanB-R	GATTTCGTTCCTCGACC			
mupA	Altered isoleucyl-tRNA synthetase; mupirocin resistance<br>Conventional PCR	*mupA-F*	TATATTATGCGATGGAAGGTTGG	458	94 °C for 4 min; 35 cycles of 94 °C for 30 s, **TA 55 °C** for 30 s, 72 °C for 30 s; 72 °C for 7 min	[66]
		mupA-R	AATAAAATCAGCTGGAAAGTGTTG			

Ta – annealing temperature; qPCR – quantitative real-time PCR. Probe fluorophores and quenchers were modified from the original assays to match the requirements of the real-time PCR instrument used in this study.
